# Supplementary material for: Role of point-of-care tests in the management of febrile children: a qualitative study of hospital-based doctors and nurses in England
Source: BMJ Open. 2021 May 10;11(5):e044510. doi: 10.1136/bmjopen-2020-044510 (PMC8112413; doi:10.1136/bmjopen-2020-044510)
Supplement: Supplementary data [file bmjopen-2020-044510supp001.pdf]

Document version: 15.0
Document date 02/07/18
IRAS ID: 248723

Topic Guide (Clinicians): Perceptions of Hospital-based Healthcare Providers in England on the Use of Rapid Diagnostic Tests (RDTs)/Point of Care Tests (POCTs) in Febrile Infants, A Qualitative Study

LONDON SCHOOL of HYGIENE &TROPICAL MEDICINE

Participant ID Number:	Gender: Male / Female
Date (DD/MM/YY):	

Patient Information Sheet & Informed Consent:

- Overview and Purpose of Study
- Aims of Interview and Expected Duration
- Who is Involved
- Why Participant's Involvement is Important, Advantages and Disadvantages
- What Will Happen to the Results of This Study
- Questions?
- Consent Form

Before I ask you further questions, here is what we will be covering in today's interview

- To explore the medical decision-making process of healthcare providers
- Focus on the availability and roles of RDTs/POCTs in the medical decision making process
- To explore advantages/disadvantages of RDTs/POCTs vs laboratory-based versions of the tests
- Implications of using RDTs/POCTs aside from the medical decision making process
- Facilitators/barriers to the wider adoption of POCTs in assessing febrile children
- To identify characteristics in future diagnostics in managing febrile children

Li E, *et al. BMJ Open* 2021; 11:e044510. doi: 10.1136/bmjopen-2020-044510

Document version: 15.0
Document date 02/07/18
IRAS ID: 248723
Warm-up and Demographic Information: May I ask you some details about you and your background as a healthcare provider?

Job Title	
For how long have you been working in the hospital?	
Year of graduation from medical/nursing school	

Now I would like to ask you some questions about your experiences and perceptions managing febrile children

Aim	Topics & Prompts	Examples/Clarifications
To explore the medical decision-making process of healthcare providers	**How would you manage this patient?**<br>A 4-month-old infant presents with fever, asymptomatic otherwise, clinical examination unremarkable. The infant was inconsolable all morning, but currently settles with mum and feeding well, 3 non-bilious, milky vomits, vitals are within normal limits. Mum says he never vomits after feeds.<br><br><u>Prompts:</u><br>• What factors influence your decision to:<br>o Discharge the patient home or admit into the hospital?<br>o Use/not use antibiotics?<br>o Use diagnostic tests? If yes, which tests? And why?	• *RDTs/POCTs are defined as diagnostic tests that can be completed at the patient's bedside and results are typically available within 15-20 minutes.*<br>• *We are focusing specifically on RDTs/POCTs used to investigate infections in children.*

Document version: 15.0
Document date 02/07/18
IRAS ID: 248723

Focusing down on the availability and role of RDTs/POCTs in the medical decision- making process	*I understand that in this hospital the RDTs/POCTs that are available are* (SHOW CARD)<br><br>**Are these RDTs/POCTs available in your A&E department for assessing febrile children?**<br><br>**Do you use any of these tests?**<br>**(if yes)**<br>• **In what cases/presentations would you use RDTs/POCTs?**<br>• **What exact purpose do they serve?**<br><br>• **Do you think these tests should be used for these purposes? Please elaborate.**<br><br>**How do RDTs/POCTs impact your initial working diagnosis?**<br>• **Do they change your initial working diagnosis?**<br>• **Are there circumstances where do they do not change your initial working diagnosis?**<br><br>**(if no)**<br>• **Why don't you use any RDTs/POCTs?**<br><br>**In some places where CRP RDTs/POCTs have been introduced, providers sometimes still prescribe antibiotics despite testing negative, what could be the reasons for this?**<br><br>**How would you rate yourself: are you a frequent user of diagnostics in general? Of RDTs/POCTs? Why is that?**<br><br>**Are there other RDTs/POCTs you would like to have available?**	• *For example: Are the test used to identify children who:*<br>○ *Need immediate care (triage)*<br>○ *Need additional confirmatory tests (screening)*<br>○ *Should be gathered (cohorted)*<br><br>• *For example, referring to the initial case scenario with a 4 month old febrile child and a rapid CRP test yielding 5 mg/L.*

Li E, *et al. BMJ Open* 2021; 11:e044510. doi: 10.1136/bmjopen-2020-044510

Document version: 15.0
Document date 02/07/18
IRAS ID: 248723

To explore the perceived advantages and disadvantages of RDTs/POCTs and lab-based versions of the tests	**What do you consider to be the advantages and disadvantages about current RDTs/POCTs compared to their laboratory-based equivalents?**<br><br>**Are the tests used instead of equivalent lab tests (to decide, for example, on hospital admission or prescription of antibiotics?)**	• *Examples of RDTs/POCTs and lab equivalent:*<br>○ *Urine dipstick vs. microscopy*<br>• *What about:*<br>○ ***Need for training (is this factor a disadvantage?)***<br>○ ***Parental acceptance***<br>○ ***Route of obtaining sample***<br>○ *Time to get results*<br>○ *Costs*<br>○ *The fact that some clinicians find useful to have a delay between ordering the test and getting results, to allow watchful observation of the clinical evolution*

Document version: 15.0
Document date 02/07/18
IRAS ID: 248723

To explore wider implications of using RDTs/POCTs, aside from the medical decision-making process	**What are the expectations of parents when they present to hospital with a febrile child?**<br><br><u>Prompts</u><br>• With regards to:<br>○ the use of diagnostic tests? (e.g. do parents ask you for these tests?)<br>○ prescription of antibiotic?<br><br>• **How do these expectations impact on your decision to use RDTs/POCTs?**<br><br><u>Prompts</u><br>(ST. MARY'S ONLY – If participant says no)<br>• **If CRP RDTs/POCTs were available to you how would parental expectations affect your decision to use the tests?**<br><br>**Do you think there is a high risk of litigation in the UK? How does it affect your use of diagnostics and RDTs/POCTs?**<br><br>**Does the use of RDTs/POCTs have an impact on your relationship with parents and children?**<br><br><u>Prompts</u><br>• If CRP RDT/POCT were available to you, would it have an impact on your relationship with parents and children? If yes, please describe it to me.	

Document version: 15.0
Document date 02/07/18
IRAS ID: 248723

	**What other factors influence you on deciding whether to use RDTs/POCTs in children?**<br><br>**How do RDTs/POCTs fit within the consultation process?**<br><br>**What is the influence of other colleagues using the tests; are there influential colleagues? Who are they?**	• ***What about the role of guidelines?***<br>• ***What about previous adverse/memorable cases/difficult cases?***<br><br>*For example:*<br>• **Is the use of RDTs/POCTs disruptive in your routine clinical practice?**<br>• *What is the impact on patient flow?*<br>• *What happens whilst waiting for results?*<br>• *Who decides to request and administer the tests (e.g. nurses, doctors)?*<br>• *Who is responsible for acting on tests results?*

Li E, *et al. BMJ Open* 2021; 11:e044510. doi: 10.1136/bmjopen-2020-044510

Document version: 15.0
Document date 02/07/18
IRAS ID: 248723

To identify facilitator(s) and barrier(s) to the wider adoption of POCTs for use in febrile children	**In your workplace, who decides on the introduction of new diagnostic tests?**<br><br>**What is or could be your role in deciding whether or not to introduce a new test?**<br><br>**What helps or hinders the adoption of additional POCTs in your work place?**<br><br><u>Prompts:</u><br>• Do you think barriers, if any, should be overcome?<br>• If yes, how could they be overcome?	• For example:<br>• <u>A facilitator</u> would be to have several devices so people do not need to queue up to use the POCT.<br>• <u>A barrier</u> would be if patients need to pay out of pocket for the test to be used<br>• What about accessibility, time pressure, accuracy of the POCTs? Staff factors? Cost factors? Hospital factors? Patient factors? Training? Physical Space? Other implications of introducing RDTs/POCTs in your workplace?
	**Some RCTs/POCTs are used more widely in other countries. Why are these used less in the UK despite being approved for use?**<br><br>**In your department, what is your perception of the clinical culture towards RDTs/POCT technology? Can you elaborate? By whom?**<br><br>**If you were given more autonomy in your practice at the A&E, do you think you would use more/less RDTs/POCTs? (Nurses)**<br><br>**Does your department have some autonomy from the hospital to decide on the introduction of RDTs/POCTS?**	• For example, CRP POCT is used more in the Netherlands.<br><br>• For example: open vs. conservative<br><br>• Autonomy: Orders for certain tests do not require a clinician to sign off on or administer.

Document version: 15.0
Document date 02/07/18
IRAS ID: 248723

To identify characteristics of future diagnostics for the management of febrile children	**Is there a need for novel diagnostic tests (POCTs or non-POCTs) that would help you in the management of febrile children?**<br><br>**For future RDTs/POCTs, what characteristics need to be improved to make you want to use them?**	• *What about:*<br>○ *Distinguishing between viral and bacterial infections*<br>○ *Predicting risk of developing severe disease*<br>○ *Differentiate between gram negative and gram positive infections*<br>○ *Identify bacterial species*<br>○ ***What is the maximum turnaround time for results you would find acceptable?***<br>○ ***What type of sample (blood, saliva, urine)?***

Closing: Is there anything else that you think is important about using diagnostic tests in the management of febrile children that we have not talked about?

- Summarize Covered Domains
- Any Other Questions/Concerns?
- Ensure participant has copies of patient information sheet and consent forms; provide any additional information as needed
- Thank interviewee for their input and participation.
