# Supplementary material for: Role of point-of-care tests in the management of febrile children: a qualitative study of hospital-based doctors and nurses in England
Source: BMJ Open. 2021 May 10;11(5):e044510. doi: 10.1136/bmjopen-2020-044510 (PMC8112413; doi:10.1136/bmjopen-2020-044510)
Supplement: Supplementary data [file bmjopen-2020-044510supp002.pdf]

Supplement 2: Sampling Matrix

**Healthcare workers**	**Hospital Site**		**Total**
	SMH, London	GNCH, Newcastle	
Nurses - Paediatric Emergency Department	2-3	2-3	4-6
Consultants - Paediatric Emergency Department	2-3	2-3	4-6
Consultants – General Paediatrics	2-3	2-3	4-6
Trainee doctors - Paediatrics*	5-6	5-6	10-12
**Total**	11-15	11-15	**22-30**

SMH: St Mary's Hospital London
GNCH: Great North Children's Hospital Newcastle
