# Supplementary material for: Role of point-of-care tests in the management of febrile children: a qualitative study of hospital-based doctors and nurses in England
Source: BMJ Open. 2021 May 10;11(5):e044510. doi: 10.1136/bmjopen-2020-044510 (PMC8112413; doi:10.1136/bmjopen-2020-044510)
Supplement: Supplementary data [file bmjopen-2020-044510supp003.pdf]

Supplement 3: Initial coding lists

Emerging theme	Codes	
	**Researcher: QL**	**Researcher: EL**
1. Trust in Clinical Judgement Over Diagnostics	RDTs not in isolation of acumen	Results to justify preliminary clinical judgements
	clinical acumen valued	Clinical examination/impression still more trusted
	assessment-dependent decision	Severity of clinical symptoms & signs
	Trust in clinical judgement	Decision to treat still dependent on clinical presentation
	Importance of examination and presentation	
	Tests and observations as clinical tools	
2. Perceived limitations of current POCTs	Worry for "grey" results	Clinical examination/impression still more trusted
	Concerns for interpreting results	CRP lag in elevation
	Time-consuming to obtain sample (i.e., urine dip)	Decision to treat still dependent on clinical presentation
	Machine need calibration	Results ambiguous/contradictory to initial impression
	Repeating test for ambiguous results	Difficult to interpret/use
	Cycle of repeat testing	"Grey areas", intermediate values
	Machine breaks down	Does not give overall diagnostic assessment of patient's condition
	Waiting for results	Forms "part of the picture" only
	Losing sample	
	Forgetting to input results	
	Concerns for falsely reassuring parents	
	'Treat the person, not the number'	
	Need for clinical picture (whole picture)	
3. Implementation of POCTs		
3a. Facilitators to introduction	Need clear guidelines for patient management	Increase acceptability for parents/child
	Need for evidence	
	Staff Buy-In	
	Need for staff education	
	Need for staff awareness	
	Need for training	

	Need training for test interpretation	
	Expanding nurses' roles	
	Nurse autonomy for RDTs	
	Need for nurse-led RDTs	
	RDTs for first point of patient contact	
	Need training to run test	
3b. Barriers to introduction	Fear of overreliance	Not actually that much faster than labs
	Overuse due to ease	Not as much information as labs
	False Positives	Results not as reliable/accurate as labs
	Fear of Overtreating	Over-reliance/use of POCTs
	Over-reassurance	False reassurances for clinicians and parents
	Fear use will undermine clinical judgement	Deterioration of clinical skills and judgement
	Concern for test quality control	Potential for overtreatment of patients
	High Staff turn-over	
	Difficult to coordinate training	
4. Current Roles of POCTs		
4a. Clinical management/Diagnostic Roles	Quick Rule-Out Tool (urine dip)	Used to monitor progress/response to treatment
	Aid antibiotic decision	Results clearly indicate potential diagnosis
	Prevent antibiotic misuse	Results to justify preliminary clinical judgements
	Reassurance for holding off antibiotics	Results support clinician's judgement
	Aid Clinicians’ Stewardship Role	
	Prevent rapid deterioration	
	Aid Justification of antibiotics (urine dip)	
4b. Hospital Logistics/Practical Roles	Speediness (in general)	Reduce patient waiting times
	Speediness of results	Allows faster clinical decision making
	Speediness of decision-making	Avoids sampling inconveniences of lab-tests
	Quicker admission and discharge time	Patient cohorting to appropriate beds/cubicles
	Alleviate A&E pressure	
	Less wait time for parents	
	Reduce observation period	
	Quick status check in wards (blood gas, potential for CRP)	
	Quick bed allocation (cohorting) (RSV)	
4c. Parent-clinician communication role	Reassurance for Parents	Improves communication between parents and doctors
	Parental expectations of diagnosis and treatment	Parents want evidence from "a test"
	Aid in explaining decision-making	Results reduce parental anxiety

	Parental anxiety may influence RDT usage	When don't parents trust a clinician's judgement
	RDTs role to decrease parental anxiety	Parents do not care what test is used
	Address parents' "fever-phobia"	
	RDT as reassurance	
	RDT use decision: a 'middle ground'	
	Decisions based on collaboration	
5. Determinants of Current POCT Usage	Parental expectations of diagnosis and treatment	Clinical decisions not influenced by fear of litigation
	Parental anxiety may influence RDT usage	Clinical decisions influenced by fear of litigation
	Decisions based on collaboration	Clinical decisions not influenced by parental expectations
	RDT use decision: a 'middle ground'	Clinical decisions influenced by parental expectations
	Colleague's Influence	Want evidence from "a test"
	Junior doctors Use More RDTs	Parental Concerns
	Decrease in frequency with seniority (doctors)	Young age of patient
	Tools for less experienced (doctors)	Unclear infectious foci
	Increase in frequency with autonomy (nurse)	
6. Characteristics of Future POCTs		
6a. Performance properties	Need to know reliability and specificity	More information from one POCT
	Importance of PPV and NPV	Results should make a difference in clinical course/management
	Need for test to change management	Should match gold-standard of lab-tests
	Need for decreasing ambiguity	Provides additional/different information from lab tests
6b. Practical use properties	Easy to act on binary results	Easy to interpret results
		Positive or negative results
		Numeric thresholds are ambiguous
		Need for analysers
		Minimal amounts of sample needed
		Painless/non-invasive for child
		Not time consuming to obtain
		Easier to get than lab-tests
