# Supplementary material for: Role of point-of-care tests in the management of febrile children: a qualitative study of hospital-based doctors and nurses in England
Source: BMJ Open. 2021 May 10;11(5):e044510. doi: 10.1136/bmjopen-2020-044510 (PMC8112413; doi:10.1136/bmjopen-2020-044510)
Supplement: Supplementary data [file bmjopen-2020-044510supp004.pdf]

Supplement 4: Example of summary matrix for the emergent theme "Trust in clinical acumen"

Participant	Initial coding	Sub-theme	Quote
ED_C2N	Assessment based decision	Patient management is based more on clinical findings than on diagnostic tests	To be honest with you, ED we really aren't going to get any diagnostic tests back. If the child is unwell, the child is going to be admitted. I might send some baseline inflammatory markers, so full blood count, CRP, Basic electrolytes, obviously blood cultures if their child is very febrile or if I'm concerned that they are shocked, urine sample. But beyond that, most of my management is going to be clinical grounds rather than treating the test result.
ED_N3N	Assessment based decision	Patient management is based more on clinical findings than on diagnostic tests	Even if the CRP was 5, that wouldn't mean you wouldn't then go on and do a septic screen. So it very much depends on the baby and how he looks and how he changes because...they change very very quickly.
ED_C1L	Assessment-based decision, RDT not in isolation	Diagnostic tests are just a complement of clinical assessment, they should not be used in isolation	So just the importance of making sure that it's still all about clinical assessment, and test are just an additional thing to that, that are really valuable and helpful.
ED_C1L	clinical acumen valued	Diagnostic tests are just a complement of clinical assessment, they should not be used in isolation	And inflammatory markers are a guide, but you know, we can get caught out where inflammatory markers can be low, and the child can still be very unwell. So it depends on the whole clinical acumen thing.
ED_C2L	Clinical acumen valued	?	I think you kind of go off clinically on what the case looks like.
ED_J2L	Clinical acumen valued	Patient management is based more on clinical findings than on diagnostic tests	...I'd prefer to be clinically led cuz if I was saying there was a number, CRP or the white count, which I may worry about sepsis. Then it could be argued that shouldn't you be worried even if those numbers are normal given that we know that the sensitive is particularly early in serious infection.
ED_N3N	Clinical acumen valued	Patient management is based more on clinical findings than on diagnostic tests	To obviously get the cultures and some things like that, and make sure we start them on sensitive to the particular infection they had. But, it would be a clinical determination on how unwell the child was and whether or not we were worried about them. It might put us a little direction on what the focus of the infection.
ED_J1N	Importance of examination and presentation	Patient management is based more on clinical findings than on diagnostic tests	I suppose there is always what you are seeing...you shouldn't be treating the numbers...you should be treating the patient so there is always a discrepancy between what you are seeing in your patient and your results. [J1N]
ED_C1L	Importance of examination and presentation	Patient management is based more on clinical findings than on diagnostic tests	Yeah so, first of all, it would be on the history and examination and what he looks like in front of me.
ED_N1L	Importance of examination and presentation	Sicker children can be identified clinically	And, it's usually, you're more aware of those children at triage, because usually the sicker children are having very abnormal obs. You can their respiratory efforts are a lot more increased. So you can usually differentiate between those children, roughly know that child...you know...this baby may need watching for 24 hours in our CDU, or it may need admission to the ward. You can usually differentiate between those two.
ED_J1N	Importance of examination and presentation	Patient management is based more on clinical findings than on diagnostic tests	Then I'd concentrate on my history and examination more than any other tests at that point.

ED_J1N	Importance of examination and presentation	Patient management is based more on clinical findings than on diagnostic tests	I think I always base the majority of my differential diagnosis on the history and examination really in a child like that.
ED_J1N	Importance of examination and presentation	Patient management is based more on clinical findings than on diagnostic tests	like I said, I think basically a lot of what I do is based on history and examination...the essential skills that doctors need to know. I trust my history and examination quite a bit.
ED_C1L	RDT not used in Isolation	Diagnostic tests are just a complement of clinical assessment, they should not be used in isolation	So I think clinical picture is still the most valuable tool and we have to make sure that people are trained in recognition of the unwell child, and use tests to augment them.
ED_C2L	RDT not used in Isolation	Diagnostic tests are just a complement of clinical assessment, they should not be used in isolation	people try to see CRP so they would use it as a kind of tracking rather than a stand alone. So, to be honest, I've not seen that many people treating anybody based on the CRP without some kind of clinical reasoning for doing so. If the patient looks unwell, you'd treat them.
ED_J1N	RDT not used in Isolation	Diagnostic tests are just a complement of clinical assessment, they should not be used in isolation	But I think if we do bloods, it is always together with history and examination just to aid your decision making. I don't think it makes your decision.
ED_C1N	RDT not used in Isolation	Diagnostic tests are just a complement of clinical assessment, they should not be used in isolation	Umm...yeah, I think that they can't be taken in isolation and I think that as long as they are used along with clinical judgement and after their assessment of the patient, taking into account all of the other factors such as their observations, their examination, the history that you've got, the parental concern, I think they are all very useful to make a clinical decision.
ED_C1N	RDT not used in Isolation	Diagnostic tests are just a complement of clinical assessment, they should not be used in isolation	Just the basic clinical skills that doctors...I think POCTs supplement what we do as doctors rather than replace it. I don't think they can be used in isolation.
ED_C1L	RDTs not used in Isolation	Diagnostic tests are just a complement of clinical assessment, they should not be used in isolation	I think that they can't be taken in isolation. I think that as long as they are used along with clinical judgement. And after their assessment of the patient. And taking into account all of the other factors such as their observations, their examination, the history that you've got, the parental concern. I think they are all very useful to make a clinical decision.
ED_C1L	RDTs not used in Isolation	Diagnostic tests are an alternative or complement of clinical assessment	So I think, you know, that's great that they are easy to do and that we get results fairly quickly but to think of the use of them in the same context where we happen to actually bleed a child...so we can use our clinical acumen as well.
ED_N2N	RDTs not used in Isolation	Diagnostic tests are just a complement of clinical assessment, they should not be used in isolation	A clinical reason to use it or if you are concerned about the patient and how they look. Then, just do the tests that are necessary so that you know you've made the right decision.
ED_C1N	RDTs not used in Isolation	Diagnostic tests are just a complement of clinical assessment, they should not be used in isolation	I don't think disruptive. Umm...as long as it's taken into context and that it's not taken in isolation.
ED_C1L	Tests and Observations (paired) as clinical tools	Clinical observation is used as a diagnostic tool	But we use that a lot. Any child with a fever without a very good focus. And even sometimes if they've got a respiratory tract focus, you might still want to make sure that we are...and if its clear. Often it's good to wait for them to wait for them to wee anyway. If there's a story of...umm....reduced fluid intake.

ED_C1L	Tests and Observations (paired) as clinical tools	Clinical observation is used as a diagnostic tool	what the point of doing the test is, explain what the results are, and say it's kind of here or there. And then, if the...again, if you are clinically worried, you would keep them for observation but maybe not treat.
ED_C1L	Tests and Observations (paired) as clinical tools	Clinical observation is used as a diagnostic tool	I can't remember exactly but maybe the CRP was 20-30. And then something else. But they were persistently tachycardic, but the urine was clear. And we do a chest x-ray and that was clear. And there was no meningitis or anything like that. And, the child didn't look septic or unwell. Just tachycardic. And in the end, we just observed them without treating them, and the tachycardic settled and then they went home. So, sometimes with pediatrics particularly, think, you just need a bit more time and such things.
ED_C1L	Tests and Observations (paired) as clinical tools	Clinical observation is used as a diagnostic tool	Regardless of how obvious the actual diagnosis is. So I think to help augment it, that would be really valuable though cuz it would help you maybe filter out some of those ambic [ambulatory] kids that you would admit but could actually could go home. You know, but then, actually, period observation is a good active treatment I think in pediatrics. I
ED_C2L	Tests and Observations (paired) as clinical tools	Clinical observation is used as a diagnostic tool	RDTs get people moving through quicker. Yeah. But I think in peds, we accept that our flow is often a bit disruptive. Cuz in pediatrics especially, there is quite a big role for a period of observation especially in febrile children.
ED_J1L	Tests and Observations (paired) as clinical tools	Clinical assessment and observation are more valuable than CRP	So I think that if I'm at the point where I want more information and I want to know what the CRP, I automatically want to observe them for a few hours, so it doesn't actually make that much of a difference to my working practice because I put less emphasis on the test than I do on the observations.
ED_J1N	Tests and Observations (paired) as clinical tools	Clinical observation is used as a diagnostic tool	So the child is usually just waiting with the parents. Sometimes it's useful because we can just observe them and see how they go for their fever or if we are waiting for anything else which is the urine sample or a fluid challenge. So sometimes, it doesn't affect the length of stay in the hospital cuz we were observing them anyway.
ED_C1N	Tests and Observations (paired) as clinical tools	Diagnostic tests are an alternative or complement of clinical assessment	So I think having useful tests and then documentation of how they have presented to you is essential. And whether that's the POCTs or observations and clinically document them in the notes the findings and what you've discussed.
ED_C2L	Trust in clinical judgement	Patient management is based more on clinical findings than on diagnostic tests	if they didn't, it would go more on how well they looked clinically.
ED_C1N	Trust in clinical judgement	Patient management is based more on clinical findings than on diagnostic tests	Where I can, I like to manage on my clinical judgement in looking at the child and my observational skills.
ED_C2L	Trust in clinical judgement	Patient management is based more on clinical findings than on diagnostic tests	But again, I don't find CRP that useful in that way, cuz I found that most people would go clinically on how the patient is like.
ED_J1N	Trust in clinical judgement	Patient management is based more on clinical findings than on diagnostic tests	I think you should base it on your clinical skills rather than investigating.

ED_C1N	Trust in clinical judgement	Patient management is based more on clinical findings than on diagnostic tests	I think it's good to use the test, but I think it doesn't replace clinical judgement and thorough clinical assessment.
ED_C1N	Trust in clinical judgement	Patient management is based more on clinical findings than on diagnostic tests	I think RDTs if you're concerned as a clinician, if even a test negative you should act on that concern.
ED_C1L	Trust in clinical judgement, RDT not used in isolation	Diagnostic tests are just a complement of clinical assessment, they should not be used in isolation	I guess, I try to base my differential diagnosis on clinical...on history and examination first. And if it's really clear, the POCTs augment what your management plan is going to do anyway. Cuz you're just going to treat based on that anyway.
ED_C1L	Trust in clinical judgement, RDT not used in isolation	Patient management is based more on clinical findings than on diagnostic tests	I guess anyway you go with your clinical picture,
CON1N	Clinical examination/impression still more trusted	Patient management is based more on clinical findings than on diagnostic tests	I think if we came back to that one, even if the CRP was low on POCT, I think I would still act because I know that, I know all the time that a lab CRP can be done and they can be low and we still get signs of an infection
SHO1L	Clinical examination/impression still more trusted	Clinical assessment and observation are more valuable than a POCT	And actually, I think the feeling is that having two hours with the patient is often more valuable than the result that you get back because you just get the time to see things for a bit longer, and just assess that baby or that child a few more times. […] I don't want to be able to see a child and send them home in 10 minutes, because I'll probably miss something. I'd much prefer if I saw a child and saw them for two hours, and pop my head in a few times and then have some blood results back that reassures me to let the child go home.
REG2N	Clinical examination/impression still more trusted	Clinical assessment and observation are more valuable than a POCT	I don't think so. Ummm, I think probably because on what I've touched on before. So I think the febrile children that you see fall into a few categories. So I think you get the children who are febrile and there is, you find a focus to their infection and based on that it's, it's normally, you're normally able to say if it's going to be more viral and kind of self-limiting, or this is more likely to be bacterial and you'd give the appropriate antibiotics depending on where the infection was. Then you get children who you can't find a focus but they look well. And if you've got a child who exams well with normal obs, then I would tend to do a period of observations, or if they look completely well, home with clear safety net advice for the parents to bring back if they became more unwell. Or I think you get the children who look sick and look septic, and in those children, you are going to treat with antibiotics. I was thinking, I couldn't think of like specific groups of children where a POCT would like really help you, help me decide on whether to give antibiotics or not.
