# Supplementary material for: Role of point-of-care tests in the management of febrile children: a qualitative study of hospital-based doctors and nurses in England
Source: BMJ Open. 2021 May 10;11(5):e044510. doi: 10.1136/bmjopen-2020-044510 (PMC8112413; doi:10.1136/bmjopen-2020-044510)
Supplement: Supplementary data [file bmjopen-2020-044510supp005.pdf]

BMJ Publishing Group Limited (BMJ) disclaims all liability and responsibility arising from any reliance placed on this supplemental material which has been supplied by the author(s)


# COREQ (COnsolidated criteria for REporting Qualitative research) Checklist

A checklist of items that should be included in reports of qualitative research. You must report the page number in your manuscript where you consider each of the items listed in this checklist. If you have not included this information, either revise your manuscript accordingly before submitting or note N/A.

| Topic | Item No. | Guide Questions/Description | Reported on Page No. |
|---|---|---|---|
| **Domain 1: Research team and reflexivity** | | | |
| *Personal characteristics* | | | |
| Interviewer/facilitator | 1 | Which author/s conducted the interview or focus group? | p3 (line 41) |
| Credentials | 2 | What were the researcher's credentials? E.g. PhD, MD | p10 (line 299- |
| Occupation | 3 | What was their occupation at the time of the study? | As above |
| Gender | 4 | Was the researcher male or female? | p3 (line 141 |
| Experience and training | 5 | What experience or training did the researcher have? | p2 (line114-11 |
| *Relationship with participants* | | | |
| Relationship established | 6 | Was a relationship established prior to study commencement? | p3 (line 128-1 |
| Participant knowledge of the interviewer | 7 | What did the participants know about the researcher? e.g. personal goals, reasons for doing the research | p3 (line128-13 |
| Interviewer characteristics | 8 | What characteristics were reported about the inter viewer/facilitator? e.g. Bias, assumptions, reasons and interests in the research topic | p10 (line 299- |
| **Domain 2: Study design** | | | |
| *Theoretical framework* | | | |
| Methodological orientation and Theory | 9 | What methodological orientation was stated to underpin the study? e.g. grounded theory, discourse analysis, ethnography, phenomenology, content analysis | p2 (lines109-1 |
| *Participant selection* | | | |
| Sampling | 10 | How were participants selected? e.g. purposive, convenience, consecutive, snowball | p2 (lines124-1 |
| Method of approach | 11 | How were participants approached? e.g. face-to-face, telephone, mail, email | p3 (lines 128- |
| Sample size | 12 | How many participants were in the study? | p4 (line 160) |
| Non-participation | 13 | How many people refused to participate or dropped out? Reasons? | p4 (line 160) |
| *Setting* | | | |
| Setting of data collection | 14 | Where was the data collected? e.g. home, clinic, workplace | p3 (lines 140- |
| Presence of non-participants | 15 | Was anyone else present besides the participants and researchers? | p3 (line 139) |
| Description of sample | 16 | What are the important characteristics of the sample? e.g. demographic data, date | p2-3 (lines120 |
| *Data collection* | | | |
| Interview guide | 17 | Were questions, prompts, guides provided by the authors? Was it pilot tested? | p2 (lines115-1 |
| Repeat interviews | 18 | Were repeat inter views carried out? If yes, how many? | N/A |
| Audio/visual recording | 19 | Did the research use audio or visual recording to collect the data? | p3 (line 140) |
| Field notes | 20 | Were field notes made during and/or after the inter view or focus group? | N/A |
| Duration | 21 | What was the duration of the inter views or focus group? | p4 (line161) |
| Data saturation | 22 | Was data saturation discussed? | p3 (lines 137- |
| Transcripts returned | 23 | Were transcripts returned to participants for comment and/or | N/A |

| Topic | Item No. | Guide Questions/Description | Reported on Page No. |
|---|---|---|---|
| | | correction? | |
| **Domain 3: analysis and findings** | | | |
| *Data analysis* | | | |
| Number of data coders | 24 | How many data coders coded the data? | p3 (lines 144- |
| Description of the coding tree | 25 | Did authors provide a description of the coding tree? | Suppl. 3 |
| Derivation of themes | 26 | Were themes identified in advance or derived from the data? | p3 (lines 144- |
| Software | 27 | What software, if applicable, was used to manage the data? | N/A |
| Participant checking | 28 | Did participants provide feedback on the findings? | N/A |
| *Reporting* | | | |
| Quotations presented | 29 | Were participant quotations presented to illustrate the themes/findings? Was each quotation identified? e.g. participant number | Boxes 1 and [illegible] |
| Data and findings consistent | 30 | Was there consistency between the data presented and the findings? | N/A |
| Clarity of major themes | 31 | Were major themes clearly presented in the findings? | Table 2 |
| Clarity of minor themes | 32 | Is there a description of diverse cases or discussion of minor themes? | p7-8 (lines 23[illegible] |

Developed from: Tong A, Sainsbury P, Craig J. Consolidated criteria for reporting qualitative research (COREQ): a 32-item checklist for interviews and focus groups. *International Journal for Quality in Health Care*. 2007. Volume 19, Number 6: pp. 349 – 357

**Once you have completed this checklist, please save a copy and upload it as part of your submission. DO NOT include this checklist as part of the main manuscript document. It must be uploaded as a separate file.**
