# Supplementary material for: Role of point-of-care tests in the management of febrile children: a qualitative study of hospital-based doctors and nurses in England
Source: BMJ Open. 2021 May 10;11(5):e044510. doi: 10.1136/bmjopen-2020-044510 (PMC8112413; doi:10.1136/bmjopen-2020-044510)
Supplement: Supplementary data [file bmjopen-2020-044510supp006.pdf]

Supplement 6: Measures in place to ensure safe and efficient use of POCTs in the two hospitals

All point of care testing is overseen by a POCT committee that supervises point of care testing across the NHS trusts to which the two hospitals included in the study belong: Newcastle upon Tyne Hospitals NHS Foundation Trust with regards Great North Children Hospital in Newcastle, and Imperial College Healthcare NHS trust with regard St Mary's Hospital in London.

Within the two trusts, point of care testing is subject to strict governance and must be performed to the same quality standards as all testing undertaken within the United Kingdom Accreditation Service (UKAS) accredited laboratory departments, and to the same standards of the MHRA (Medicines and Healthcare products Regulatory Agency), the NHS Litigation Authority (NHSLA), the International Organisation for Standardisation (ISO), the Royal College of Pathologists (RCPath), and the Clinical Pathology Accreditation (CPA) standards.

Chaired by the POCT Manager, the POCT committee:

- Leads and coordinates POCT activities for the Trust in conjunction with the Trust's key objectives
- Maintains training records of all POCT operators throughout the Trust and coordinates staff education and facilitates training for all POCT devices. All POCT operators must be accredited by the POCT committee by undertaking POCT recognised training prior to patient testing. Competence in using POCT devices is achieved via Initial Training – this constitutes face-to-face training by either attending a timetabled training session or with a recognised 'Cascade Trainer'. Refresher Training is required every subsequent two years to maintain that competency
- Coordinates the provision of External Quality Assurance (EQA) schemes where possible, review EQA performance and offer advice on the improvement of poor performance
- Provides advice and help with documentation to Managers of areas on policies, operating procedures, training and competencies
- Undertakes Risk assessments for POCT devices where appropriate
- Leads and coordinates the validation of new POCT devices before implementation
- Provides evaluation of the performance of POCT equipment
- Coordinates audits and inspections to provide Trust assurance of quality patient care and value for money
