# Supplementary material for: Role of point-of-care tests in the management of febrile children: a qualitative study of hospital-based doctors and nurses in England
Source: BMJ Open. 2021 May 10;11(5):e044510. doi: 10.1136/bmjopen-2020-044510 (PMC8112413; doi:10.1136/bmjopen-2020-044510)
Supplement: Supplementary data [file bmjopen-2020-044510supp007.pdf]

Supplement 7: POCTs availability at the study hospitals

**Hospital**	**Test**	**Vendor**
St. Mary's Hospital, London	Blood gas analyser	GEM 4000
	Blood glucose/ketones	Freestyle Precision Pro
	Urinalysis	Siemens Multistix 8 SG
	CRP*	Alere Afinion AS100
Great North Children's Hospital, Newcastle	Urine dipstick	Combur7 test
	Urine ketones	Ketodiastix Ascensia
	Urine microscopy	
	Blood glucose	Accucheck inform II
	Blood gas analyser	GEM premier 5000
	Influenza & RSV	Cepheid GeneXpert

*CRP POCTs were not available at St. Mary's Hospital at the time of study, however they previously had been available as part of a pilot
