# Supplementary material for: Role of point-of-care tests in the management of febrile children: a qualitative study of hospital-based doctors and nurses in England
Source: BMJ Open. 2021 May 10;11(5):e044510. doi: 10.1136/bmjopen-2020-044510 (PMC8112413; doi:10.1136/bmjopen-2020-044510)
Supplement: Author's manuscript [file bmjopen-2020-044510.draft_revisions.pdf]

BMJ Open is committed to open peer review. As part of this commitment we make the peer review history of every article we publish publicly available.

When an article is published we post the peer reviewers' comments and the authors' responses online. We also post the versions of the paper that were used during peer review. These are the versions that the peer review comments apply to.

The versions of the paper that follow are the versions that were submitted during the peer review process. They are not the versions of record or the final published versions. They should not be cited or distributed as the published version of this manuscript.

BMJ Open is an open access journal and the full, final, typeset and author-corrected version of record of the manuscript is available on our site with no access controls, subscription charges or pay-per-view fees (http://bmjopen.bmj.com).

If you have any questions on BMJ Open's open peer review process please email info.bmjopen@bmj.com

BMJ Open

Point-of-Care-Testing in Febrile Children Presenting to Hospitals: A Qualitative Study.

Journal:	*BMJ Open*
Manuscript ID	bmjopen-2020-044510
Article Type:	Original research
Date Submitted by the Author:	04-Sep-2020
Complete List of Authors:	Li, Edmond; Imperial College London Faculty of Medicine, Surgery & Cancer; London School of Hygiene and Tropical Medicine Faculty of Infectious and Tropical Diseases, Clinical Research Department<br>Luu, Queena; London School of Hygiene and Tropical Medicine Faculty of Infectious and Tropical Diseases, Clinical Research Department<br>Dewez, Juan ; London School of Hygiene and Tropical Medicine, Clinical Research Department<br>Emonts, Marieke; Great North Children's Hospital, Paediatric Immunology, Infectious Diseases & Allergy, Newcastle upon Tyne Hospitals NHS Foundation Trust<br>Maconochie, Ian; Imperial College Hospital NHS Healthcare Trust, Paediatric Emergency Department<br>Nijman, R; Imperial College Healthcare NHS Trust, Department of Paediatrics<br>Yeung, Shunmay; London School of Hygiene and Tropical Medicine Faculty of Infectious and Tropical Diseases, Clinical Research Department
Keywords:	QUALITATIVE RESEARCH, INFECTIOUS DISEASES, Paediatric A&E and ambulatory care < PAEDIATRICS, PAEDIATRICS, Paediatric infectious disease & immunisation < PAEDIATRICS

SCHOLARONE™
Manuscripts

BMJ

I, the Submitting Author has the right to grant and does grant on behalf of all authors of the Work (as defined in the below author licence), an exclusive licence and/or a non-exclusive licence for contributions from authors who are: i) UK Crown employees; ii) where BMJ has agreed a CC-BY licence shall apply, and/or iii) in accordance with the terms applicable for US Federal Government officers or employees acting as part of their official duties; on a worldwide, perpetual, irrevocable, royalty-free basis to BMJ Publishing Group Ltd ("BMJ") its licensees and where the relevant Journal is co-owned by BMJ to the co-owners of the Journal, to publish the Work in this journal and any other BMJ products and to exploit all rights, as set out in our licence.

The Submitting Author accepts and understands that any supply made under these terms is made by BMJ to the Submitting Author unless you are acting as an employee on behalf of your employer or a postgraduate student of an affiliated institution which is paying any applicable article publishing charge ("APC") for Open Access articles. Where the Submitting Author wishes to make the Work available on an Open Access basis (and intends to pay the relevant APC), the terms of reuse of such Open Access shall be governed by a Creative Commons licence – details of these licences and which Creative Commons licence will apply to this Work are set out in our licence referred to above.

Other than as permitted in any relevant BMJ Author's Self Archiving Policies, I confirm this Work has not been accepted for publication elsewhere, is not being considered for publication elsewhere and does not duplicate material already published. I confirm all authors consent to publication of this Work and authorise the granting of this licence.

For peer review only

Point-of-Care-Testing in Febrile Children Presenting to Hospitals: A Qualitative Study.

AUTHORS

Edmond C. Li,[1,2] Queena F. Luu,[1] Juan Emmanuel Dewez,[1] Marieke Emonts,[3,4] Ian Maconochie,[5] Ruud Nijman,[5] Shunmay Yeung[1,5]

AFFILIATION

1: Clinical Research Department, Faculty of Infectious and Tropical Diseases, London School of Hygiene & Tropical Medicine, London, United Kingdom

2: Patient Safety Translational Research Centre, Department of Surgery and Cancer, Faculty of Medicine, Imperial College London, London, United Kingdom

3: Great North Children's Hospital, Paediatric Immunology, Infectious Diseases & Allergy, Newcastle upon Tyne Hospitals NHS Foundation Trust, Newcastle upon Tyne, United Kingdom

4: Translational and Clinical Research Institute, Newcastle University, Newcastle upon Tyne, United Kingdom

5: Section of Paediatric Infectious Diseases, Faculty of Medicine, Imperial College London, London, United Kingdom

6: Department of Paediatrics, St Mary's Imperial College Hospital London, United Kingdom

CORRESPONDING AUTHOR

Shunmay Yeung, Keppel Street, London WC1E 7HT, United Kingdom

For peer review only

Email: shunmay.yeung@lshtm.ac.uk

Phone: 02079272657

ABSTRACT

Objectives

The use of rapid point-of-care tests (POCTs) in patients with suspected infections is advocated to optimise antibiotics prescribing, improve patient outcomes and the use of medical resources. However, few studies have explored healthcare workers' views about their use in febrile children. The aim of this study was to explore the perceptions of hospital-based doctors and nurses regarding the use of POCTs in England.

Design

Qualitative in-depth interviews of purposively selected doctors and nurses. Data were analysed thematically.

Setting

Two university teaching hospitals in London and Newcastle.

Participants

24 participants (paediatricians, emergency-department doctors, trainee paediatricians, and nurses).

Results

There were diverse views surrounding the use of POCTs in febrile children. POCTs were seen as advantageous mainly because of their ease of use and the rapid availability of results. This was seen to lead to faster clinical decision-making; enhanced patient flow and cohorting; better antibiotic stewardship, improved care and monitoring of patients; and improved communication with parents. The advantages of POCTS were less evident when hospital laboratories could return results within 1-2 hours. Being a able to perform blood-based POCTs from finger-prick sampling was also seen as less invasive than tests requiring venous sampling. Other factors such as clinical experience and specialty, as well as

For peer review only - http://bmjopen.bmj.com/site/about/guidelines.xhtml

the availability of guidelines recommending POCT use were also perceived as influential. However, in addition to their perceived inaccuracy, participants were concerned about POCTs' inability to resolve diagnostic uncertainty or alter clinical management. These limitations reinforced the preference of many doctors for clinical acumen over diagnostics.

Conclusion

Participants expressed mixed opinions about the utility of current POCTs in the management of febrile children. If the use of POCTs is to be scaled or new tests introduced, it will be important to address concerns regarding their limitations and how their results can or should change clinical decision-making.

For peer review only

Strengths & limitations of this study

Strengths

- A specific focus on the paediatric population presenting to hospitals
- The inclusion of two hospitals in two different settings within England.
- A multi-disciplinary and diverse group of participants; perspectives were obtained from nurses and from doctors with differing levels of clinical experience and training.

Limitations

- The interviews took place at two university teaching hospitals and did not explore the views of clinicians in the community or at district general hospitals.

FUNDING DECLARATION

JED, ME, and SY are supported by PERFORM, a consortium funded by the European Union's Horizon 2020 programme, under grant agreement No. 668303. RGN was supported by NIHR Academic clinical fellowship (CF- 2015-21-016) and lectureship (CL-2018-21-007) award programme. The funding bodies

For peer review only - http://bmjopen.bmj.com/site/about/guidelines.xhtml

did not take part in the design of the study and data collection, and did not take part in the data analysis and interpretation of results. The funding bodies did not take part in the writing of the manuscript.

COMPETING INTERESTS

We declare no competing interests. EL and QL are public health specialists interested in health services research. JED, SY, RGN, ME, and IM are members of a research consortium (see below) aiming to identify, investigate, and validate new biomarkers for the management of infections.

DATA SHARING

The datasets generated and/or analysed during the current study will be made available from the corresponding author on reasonable request.

WORD COUNT: 2846

For peer review only - http://bmjopen.bmj.com/site/about/guidelines.xhtml

For peer review only

INTRODUCTION

Febrile illness is one of the most common reasons for children to present to hospital, and managing it can often be challenging.[1,2] The majority of febrile children will have self-limiting infections which can be safely managed at home, often without antibiotics.[3-5] However, a few will have potentially severe infections and identifying these children from the others can be difficult, as clinical symptoms and signs are often unspecific in children.[3] Consequently, they may be prescribed antibiotics that are not needed, subjected to invasive tests, and admitted for monitoring whilst awaiting microbiology results.[4,5] As well as the impact that these practices have on children and parents in terms of pain, distress, costs, and inconvenience,[6] this may contribute to antimicrobial resistance (AMR).[7]

The World Health Organization advocates the use of rapid point-of-care tests (POCTs) to reduce the use of antibiotics.[8] POCTs could aid clinical decision-making, limit the use of other invasive tests, and optimise the use of medical resources in general.[9] However, achieving these impacts would depend on several factors, including whether POCTs are taken up by healthcare workers and how they influence clinical decisions.[10] This cannot be assumed. For example, when malaria POCTs were introduced, many clinicians continued to prescribe antimalarials despite patients testing negative.[11]

POCTs currently available for suspected infection include urine dipstick; rapid tests for Group A Streptococcal throat infections (“rapid strep test”); rapid respiratory virus tests for Respiratory Syncytial Virus (RSV) or Influenza; and blood-based tests including C-reactive protein (CRP) and blood gas analysers.

Current research exploring the views of clinicians on the use of POCTs for managing patients with infections have predominantly focused on general practitioners (GPs) treating adult patients.[12-19] To the best of our knowledge, only one study pertains to the perceptions of hospital-based healthcare workers regarding the use of POCTs in children.[20]

For peer review only - http://bmjopen.bmj.com/site/about/guidelines.xhtml

The aim of this study was to explore the experiences and attitudes of hospital-based doctors and nurses regarding the use of POCTs in children with acute infections, and to identify factors that influence their use.

METHODS

Qualitative in-depth interviews with doctors and nurses were conducted to better understand their experiences and attitudes regarding POCT use in children with suspected infection.[21] Ontologically, our approach adhered to subtle realism, and epistemologically to interpretivism,[22,23] because we believed that although reality (i.e., the use of diagnostics) exists independently of our beliefs, the interpretation of participants' perceptions and how they make sense of their experiences were needed to ascertain what influences POCT usage.

The research team comprised of two public health professionals and five paediatricians with experience in qualitative methods. Two topic guides were developed, one for doctors and one for nurses, based on a review of the literature (Supplement 1). These were piloted with two paediatricians and two nurses at St Mary's Hospital in London. The pilot interviews were not included in the analysis. The topic guides were also adapted during data collection after regular debriefings between EL, QL, and JED to add or modify questions to explore new areas of inquiry.[24]

The participants comprised of doctors and nurses. Purposive maximum variation sampling was used, focusing on hospital-based healthcare workers with varying years of experience who make decisions about whether or not to use POCTs on febrile children. Moreover, participants worked in two socioeconomically different regions: half worked at the Great North Children's Hospital in Newcastle, and the other half at St Mary's Hospital in London. Potential participants were selected and approached by members of the research team who work in those hospitals (SY, RN, IM, ME) based on a sampling

For peer review only - http://bmjopen.bmj.com/site/about/guidelines.xhtml

matrix (Supplement 2). Those who agreed to be interviewed were followed-up by the interviewers (QL and EL) who provided a participant information sheet, obtained informed consent, and arranged the interview date. The two interviewers did not know participants beforehand. The study aimed to include 22-30 participants, a sample size which is comparable to other qualitative studies investigating healthcare workers' perceptions, and was expected to allow for data saturation.[12,13,18,25] One-on-one interviews took place between June and August 2018, and were audio-recorded.

The interview records were transcribed verbatim by EL and QL and back-checked against the recording for accuracy. The transcripts were anonymised (only participants' job titles were documented) and then analysed thematically.[24] The analysis was both deductive (i.e., partly based on pre-existing knowledge) and inductive (i.e., additional concepts were identified from the dataset). EL and QL generated two initial lists of coding from the dataset through line-by-line coding (Supplement 3). QL, EL, and JED then agreed on an analytical framework comprising the most relevant codes and used the framework to re-code the entire dataset. Matrices were used to display data related to initial themes (Supplement 4). Iterative exploration of the data identified patterns within the dataset continued until no new themes were identified. Further refinement was carried out with the rest of the research team to ensure themes were internally coherent and distinctive to produce clear descriptions and explanations of each theme.

Ethical approval was obtained from the London School of Hygiene & Tropical Medicine Ethics Committee (Ref:15040-15088). Patients and members of the public were not involved in the design of this study.

RESULTS

A total of 24 out of 27 invited healthcare workers were interviewed (Table 1); two nurses and one trainee doctor did not reply to the invitation. Participants' experience of using POCTs varied at the two hospitals. Although all participants reported routine use of urinary dipsticks, Respiratory Syncytial Virus

For peer review only

(RSV) rapid tests, and blood gas tests, some participants from St Mary's Hospital recalled also having access to CRP and rapid strep tests in past studies. (Please see Supplement 7 and 8 for description of POCT availability and oversight at the two hospitals). Most participants fully engaged with the interviews and readily shared their views on the topics of interest with little direction from the interviewers. There were a range of reasons given for and against using POCT. These have been grouped into nine themes (Table 2): six pertaining to factors encouraging the use of POCTs (Box 1) and three for factors discouraging the use of these tests (Box 2) in the management of febrile children.

Factors encouraging the use of rapid POCTs

Most participants were keen on using POCTs due to the inherent advantages they presented (Box 1).

One of the main cited advantages of POCTs was their ease of use and speed with which they can provide results when compared to hospital laboratory-based diagnostics. This contributed to improving patient flow in busy wards. However, a few did question the magnitude of this impact as some participants noted that these advantages were largely context dependent. In circumstances where laboratories were able to return results within one to two hours, some participants reported that the rapidity of POCTs was outweighed by the better perceived accuracy of laboratory-based tests; the possibility to monitor the patient's clinical evolution; or the flexibility to see other patients in parallel whilst awaiting laboratory results.

CRP POCTs and urine dipsticks were reported to support quicker decision-making, such as whether or not to prescribe antibiotics or to perform additional diagnostic tests. The speed of getting results for a rapid RSV test was valued for quickly cohorting patients to limit the spread of infections in the hospital. Obtaining results rapidly allowed clinicians to communicate results earlier, quickly justify their clinical

For peer review only - http://bmjopen.bmj.com/site/about/guidelines.xhtml

decisions, and reduce overall parental anxieties, which was felt to improve the caregivers' experience of the care provided to their child.

The ability to use rapid POCTs without the help of other doctors or nurses, and to get results independently from the laboratory, was important for many participants. Additionally, blood-based POCTs was that they could be performed on blood from a finger prick rather than venepuncture which was seen as more invasive and difficult. These attributes allowed for easier repeat testing for monitoring purposes, and provided participants with a greater sense of direct control over the care of their patients. Blood gas POCTs were a notable example of this. Aside from advantages inherent to POCTs, other factors influenced the use of POCTs. For doctors, their level of experience appeared to influence their use of diagnostics. There was a prevailing perception that less experienced clinicians used more POCTs as the tests serve as learning tools, as well as a "safety net" if their clinical acumen proved inadequate. Moreover, participants also alluded that diagnostics use, including POCTs, varied according to their perceptions of the risk for severe infections, which itself was dependent on the clinician's specialty training and the population seeking care. Some participants believed that general practitioners (GPs) used less diagnostics than emergency department paediatricians, who in turn use less diagnostics than paediatric infectious diseases specialists. This was thought to be because the patient population seen by the three types of clinicians differed, with GPs seeing proportionally fewer children with severe infections.

Finally, clinical guidelines were also seen as important. Participants reported that guidelines including recommendations regarding when to use and how to interpret POCT results in children would be highly influential in their decision to use POCTs. This was because guidelines were perceived as both providing reassurance of best practices and protection against malpractice.

For peer review only

For peer review only - http://bmjopen.bmj.com/site/about/guidelines.xhtml

Factors discouraging the use of rapid POCTs

Several participants were concerned about the accuracy of the tests (Box 2). It was commonly perceived that there were discrepancies in results when compared to laboratory tests and/or in relation to the patient's clinical presentation. This undermined clinicians' trust in POCTs, and often resulted in subsequent confirmatory laboratory tests to be performed prior to making a clinical decision. Many also cited the lack of strong evidence supporting the accuracy of POCTs for paediatric use, as several participants noted that existing POCTs were predominantly validated for adult use.

Another notable reason causing hesitancy amongst many of the interviewees about using some POCTs was their perceived inability to adequately address diagnostic uncertainty. Some participants mentioned that with current POCTs that provide numerical results (e.g. CRP levels) instead of binary results ("positive/negative"), the tests were unable to provide a definitive clinical answer when results were within an "intermediate" range. Other participants felt that current POCTs are not able to reliably predict the risk of clinical deterioration in initially well-appearing febrile children. Being able to ascertain which children were likely to deteriorate and require hospitalisation was perceived as an important feature of diagnostics for managing fever in children. Another limitation also commonly described was the inability of some POCTs to provide a more comprehensive scope of diagnostic information compared to laboratory tests. For example, RSV POCTs were able to show the presence of this pathogen only, while laboratory-based panels could provide data on several pathogens from a single sample, which was perceived to be more useful. Altogether, this failure to resolve diagnostic uncertainty hampers the ability for healthcare providers to alter their clinical decision-making. In addition to this shortcoming, even when certain POCTs are able to address diagnostic uncertainty, their results may not prove consequential enough to warrant a change in the patient's overall clinical management. A commonly referenced example of this is the Group A Streptococcus rapid test. While the test may be sensitive and specific, some participants felt that knowing the causal pathogen of tonsillitis had little influence on

For peer review only - http://bmjopen.bmj.com/site/about/guidelines.xhtml

For peer review only

their decision to prescribe antibiotics. However, other POCTs such as urine dipsticks, greatly influenced clinical management for many participants.

The overall limitations of POCTs reinforced the widely held view that healthcare workers preferred and prioritised their clinical acumen and skills (history, examination, observation of patients) over diagnostics in guiding their clinical management of febrile children.

Comparison of views between types of participants

There were no major discrepancies of views amongst different types of participants with regards to the themes summarised in Table 2, except for the following:

- The potential contribution of POCT usage in improving antibiotic stewardship was strongly emphasized by consultants and supported by nurses. Trainee doctors mentioned it as well, but with less importance.
- Consultants felt that trainees use more POCTs. This sentiment was also shared by nurses, while trainees rarely mentioned this.
- Consultants were more concerned with the difficulty of interpreting intermediate CRP results. Trainees focussed more on how CRP results can appear normal for a child at the beginning of a serious infection.
- Trainees were more eager than consultants for more clinical information to be available per POCT.
- Consultants stressed the importance of relying on their clinical acumen, more so than trainees.
- Nurses mentioned their willingness to perform more POCTs independently of doctors in the future. Consultants and trainees rarely commented on who should be performing the tests, and when they did so were not against nurses using POCTs independently.

For peer review only - http://bmjopen.bmj.com/site/about/guidelines.xhtml

DISCUSSION

This study provides insight into what frontline hospital-based healthcare workers think of POCTs in relation to managing children with a suspected infection, a topic on which little has previously been published. A range of reasons for and against the use of POCTs was identified. POCTs were seen as advantageous mainly because of their ease of use and the rapid availability of results. This led to faster clinical decision-making, enhanced patient flow, and improved clinical care resulting from better antibiotic stewardship, better cohorting and monitoring of patients, and improved communication with parents. However, these advantages were less evident when laboratories were able to return results within a few hours. Participants also reported that being less invasive, POCTs performed on finger-prick blood helped to improve their acceptability by clinicians, parents, and children. Other factors such as clinical experience and specialty, as well as the availability of guidelines recommending POCT use were also perceived as influential. Participants also reported a number of limitations of POCTs, particularly regarding their perceived lack of accuracy and failure to solve diagnostic uncertainty. The shortcomings of POCTs meant that they were seen as having a limited role in guiding or changing overall clinical management, and reinforced the overarching view that clinicians should trust their clinical acumen more than diagnostics.

The only other study that we were aware of , which specifically explored the views of paediatricians and nurses about rapid POCTs usage in children, found similar results: participants valued their ease and speed of use, which enabled early treatment, cohorting, decreasing antibiotic prescription, and better communication with parents [20}.. Some participants were also concerned about the accuracy of the tests and about the limited changes in clinical management enabled by rapid POCTs

For peer review only

For peer review only - http://bmjopen.bmj.com/site/about/guidelines.xhtml

Some studies exploring the views of GPs reported similar advantages and concerns, especially regarding the accuracy of tests [12,14,20,25,26,27]. However, there were conflicting results in terms of the perceived invasiveness of POCTs: two studies found that GPs would not use blood-based POCTs because they felt finger pricking was too invasive in children [12,25], while another study reported that GPs, who had the opportunity to try the tests, perceived the pain to be short-lived and recounted that children were actually interested by the use of rapid tests during consultations [28]. Other GPs felt it was easy to persuade parents that antibiotics were not needed compared to adult patients, limiting the need to use rapid POCTs for this purpose [25]. Some GPs reportedly also feared that the use of POCTs could actually increase antibiotic prescription [27,28]. Finally, in contrast to our finding that for some paediatricians the perceived limitations of POCTs reinforced their preference of relying on their clinical acumen over diagnostics, some of studies with GPs reported that there were concerns that the perceived advantages of POCTs could lead to the tests being over enthusiastically adopted and used, which could potentially undermine clinical acumen [13,14,27].

While the majority of respondents highlighted the inherent speed advantages of current POCTs, there is also a notable recognition of the performance shortcomings and contextual factors (e.g. organisational aspects, practice norms) which need to be considered in understanding their current and potential role.. Simply increasing POCTs availability in hospitals may not improve the workflow or the overall quality of care. The inability to predict risks of clinical deterioration, the perceived poor diagnostic accuracy, and limited scope of diagnostic information per test, seemed to relegate existing POCTs to a largely supportive rather than diagnostically indicative tool in the overall clinical decision-making process. Only when these performance gaps are bridged would POCTs be used with greater regularity and confidence. Moreover, the clinical culture in England emphasising clinical examination and acumen over diagnostics suggests that POCTs were unlikely to be overly relied upon even if they were made more readily

For peer review only - http://bmjopen.bmj.com/site/about/guidelines.xhtml

available. Rather, the availability of robust, paediatric-specific evidence and clear guidelines, would likely be more persuasive . Of note, at the time of the study, neither rapid POCT CRP nor rapid strep tests were mentioned in the guidelines of either hospital; urine dipstick and blood gas analysis were specified guidelines for clinical care; and RSV POCT for cohorting of admissions during the winter months.

For healthcare policymakers and tests developers, these findings indicate both existing and potential roles which can be filled by POCTs in hospitals and the barriers to the use of the tests. Taking into account the views expressed in this study will hopefully contribute to ensuring that the development and implementation of future tests is better aligned to clinicians' needs.

We purposively sampled a diverse range of healthcare providers across two hospitals servicing differing patient populations in England. To ensure a holistic, multi-disciplinary understanding of the views pertaining to POCT use when treating paediatric patients, both nurses and doctors were interviewed. We achieved a balanced sample of participants with varying levels of clinical experience. However, the scope of this study was restricted to two teaching hospitals in England with a history of engaging in research for novel diagnostic tests. This may have thus skewed the participants' views due to their greater exposure and access to innovative diagnostics. We did not interview other sub-specialty paediatricians such as paediatric infectious disease specialists. Moreover, the restriction of interviews to these two hospitals means that we did not capture the views of other important groups of clinicians who are frequented by children and their parents: GPs and paediatricians in district general hospitals. Finally, it was not always possible to decipher whether the participants were referring to specific POCTs or POCTs in general.

Further qualitative research with other key stakeholders, such as clinicians in other sub-specialties and those based in district general hospitals or in the community; and parents and children themselves,

For peer review only

For peer review only - http://bmjopen.bmj.com/site/about/guidelines.xhtml

would be very informative. As the use of rapid POCT seems to vary greatly in different countries it would also be very interesting to carry out similar studies in other countries. Trying to elucidate in which patients and where within the care pathway, specific POCTs would have most impact, would be useful. Finally, this study was carried out in the pre-COVID19 era. Since then the diagnostics landscape has changed dramatically and it would be very interesting to carry out a similar study now, as the views about rapid POCTs are likely to be quite different.

Conclusion

Despite POCTs being increasingly recommended to optimise the use of antibiotics and medical resources in clinical practice, hospital-based doctors and nurses expressed mixed opinions about the utility of existing POCTs in the management of children with suspected infections. Whilst the many potential advantages of POCTs were well recognised, concerns including about their accuracy and failure to solve diagnostic uncertainty or alter clinical management, limited their perceived utility. It will be important to address these concerns in the efforts to scale up the use of current tests and in the development of new tests.

AUTHOR CONTRIBUTIONS

Edmond C. Li (EL), Queena F. Luu (QL) and Juan Emmanuel Dewez (JED) contributed equally to this paper. Shunmay Yeung (SY) conceived of the study and had overall responsibility. All authors input into the design of the study and the study materials. SY, RGN, ME, and IM identified participants. EL and QL conducted and transcribed the interviews. JED, EL, and QL analysed the data and wrote the manuscript. JED, EL and SY made substantial revisions. All authors provided input into drafts of the manuscript and agree on the contents of the final version.

For peer review only

For peer review only - http://bmjopen.bmj.com/site/about/guidelines.xhtml

339 **REFERENCES**

1. Sands R, Shanmugavadivel D, Stephenson T, Wood D. Medical problems presenting to paediatric emergency departments: 10 years on. Emergency medicine journal : EMJ. 2012 May;29(5):379-82.
2. Alpern ER, Stanley RM, Gorelick MH, Donaldson A, Knight S, Teach SJ, et al. Epidemiology of a pediatric emergency medicine research network: the PECARN Core Data Project. Pediatric emergency care. 2006 Oct;22(10):689-99.
3. Van den Bruel A, Haj-Hassan T, Thompson M, Buntinx F, Mant D. Diagnostic value of clinical features at presentation to identify serious infection in children in developed countries: a systematic review. Lancet (London, England). 2010;375(9717):834-45.
4. De S, Williams GJ, Hayen A, Macaskill P, McCaskill M, Isaacs D, et al. Accuracy of the "traffic light" clinical decision rule for serious bacterial infections in young children with fever: a retrospective cohort study. BMJ (Clinical research ed). 2013;346:f866.
5. Nijman RG, Vergouwe Y, Thompson M, van Veen M, van Meurs AHJ, van der Lei J, et al. Clinical prediction model to aid emergency doctors managing febrile children at risk of serious bacterial infections: diagnostic study. BMJ : British Medical Journal. 2013;346.
6. Leigh S, Grant A, Murray N, Faragher B, Desai H, Dolan S, et al. The cost of diagnostic uncertainty: a prospective economic analysis of febrile children attending an NHS emergency department. BMC medicine. 2019;17(1):48.
7. European Centre for Disease Control, European Food Safety A, European Medicines A. ECDC/EFSA/EMA second joint report on the integrated analysis of the consumption of antimicrobial agents and occurrence of antimicrobial resistance in bacteria from humans and food-producing animals. EFSA Journal. 2017;15(7):e04872-n/a.
8. WHO. World Health Organization. Global action plan on antimicrobial resistance 2015. Available from http://www.who.int/antimicrobial-resistance/global-action-plan/en. Accessed 20/12/17.
9. Larsson A, Greig-Pylypczuk R, Huisman A. The state of point-of-care testing: a European perspective. Upsala journal of medical sciences. 2015;120(1):1-10.
10. Horvath AR, Lord SJ, StJohn A, Sandberg S, Cobbaert CM, Lorenz S, et al. From biomarkers to medical tests: the changing landscape of test evaluation. Clinica chimica acta; international journal of clinical chemistry. 2014;427:49-57.
11. Burchett HE, Leurent B, Baiden F, Baltzell K, Bjorkman A, Bruxvoort K, et al. Improving prescribing practices with rapid diagnostic tests (RDTs): synthesis of 10 studies to explore reasons for variation in malaria RDT uptake and adherence. BMJ Open. 2017;7(3):e012973.
12. Butler CC, Simpson S, Wood F. General practitioners' perceptions of introducing near-patient testing for common infections into routine primary care: a qualitative study. Scandinavian journal of primary health care. 2008;26(1):17-21.

For peer review only - http://bmjopen.bmj.com/site/about/guidelines.xhtml

13. Cals JW, Chappin FH, Hopstaken RM, van Leeuwen ME, Hood K, Butler CC, et al. C-reactive protein point-of-care testing for lower respiratory tract infections: a qualitative evaluation of experiences by GPs. Family practice. 2010;27(2):212-8.

14. Wood F, Brookes-Howell L, Hood K, Cooper L, Verheij T, Goossens H, et al. A multi-country qualitative study of clinicians' and patients' views on point of care tests for lower respiratory tract infection. Family practice. 2011;28(6):661-9.

15. Pulcini C, Pauvif L, Paraponaris A, Verger P, Ventelou B. Perceptions and attitudes of French general practitioners towards rapid antigen diagnostic tests in acute pharyngitis using a randomized case vignette study. Journal of Antimicrobial Chemotherapy. 2012;67(6):1540-6.

16. Leydon GM, McDermott L, Moore M, Williamson I, Hobbs FD, Lambton T, et al. A qualitative study of GP, NP and patient views about the use of rapid streptococcal antigen detection tests (RADTs) in primary care: 'swamped with sore throats?'. BMJ open. 2013;3(4).

17. Hardy V, Thompson M, Alto W, Keppel GA, Hornecker J, Linares A, et al. Exploring the barriers and facilitators to use of point of care tests in family medicine clinics in the United States. BMC family practice. 2016;17(1):149.

18. Hardy V, Thompson M, Keppel GA, Alto W, Dirac MA, Neher J, et al. Qualitative study of primary care clinicians' views on point-of-care testing for C-reactive protein for acute respiratory tract infections in family medicine. BMJ open. 2017;7(1):e012503.

19. Eley CV, Sharma A, Lecky DM, Lee H, McNulty CAM. Qualitative study to explore the views of general practice staff on the use of point-of-care C reactive protein testing for the management of lower respiratory tract infections in routine general practice in England. BMJ open. 2018;8(10):e023925.

20. Bustinduy AL, Jeyaratnam D, Douthwaite S, Tonkin-Crine S, Shaw R, Hyrapetian L, et al. Point-of-care tests for infectious diseases: barriers to implementation across three London teaching hospitals. Acta paediatrica (Oslo, Norway : 1992). 2017.

21. Flick U. An introduction to qualitative research. 5th ed. SAGE Publications; 2014.

22. Ritchie J, Lewis J, Nicholls CMN, Ormston R. Qualitative Research Practice: A Guide for Social Science Students and Researchers: SAGE Publications; 2013.

23. Durrand MAC, Tracy. (2014) Principles of social research . 2nd ed. Maidenhead: Open University Press.

24. Braun V, Clarke V. Using thematic analysis in psychology. Qualitative Research in Psychology. 2006;3(2):77-101.

25. Schot MJ, Broekhuizen BD, Cals JW, Brussee E, de Wit NJ, Verheij TJ, et al. C-reactive protein point-of-care testing in children with cough: qualitative study of GPs' perceptions. BJGP Open. 2018;1(4):bjgpopen17X101193.

For peer review only - http://bmjopen.bmj.com/site/about/guidelines.xhtml

26. Jones CH, Glogowska M, Locock L, Lasserson DS. Embedding new technologies in practice - a normalization process theory study of point of care testing. BMC health services research. 2016;16(1):591.

27. Turner PJ, Van den Bruel A, Jones CHD, Plüddemann A, Heneghan C, Thompson MJ, et al. Point-of-care testing in UK primary care: a survey to establish clinical needs. Family practice. 2016;33(4):388-94.

28. Van den Bruel A, Jones C, Thompson M, Mant D. C-reactive protein point-of-care testing in acutely ill children: a mixed methods study in primary care. Archives of disease in childhood. 2016 Apr;101(4):382-5.

340

For peer review only

For peer review only - http://bmjopen.bmj.com/site/about/guidelines.xhtml

TABLES

Table 1. Characteristics and numbers of participants

**Healthcare workers**	**Hospital Site**		**Total**
	SMH, London	GNCH, Newcastle	
Nurses – Paediatric Emergency Department	1	3	4
Consultants – Paediatric Emergency Department	3	2	5
Consultants – General Paediatrics	2	1	3
Trainee doctors – Senior House Officers	4	3	7
Trainee doctors – Registrars	2	3	5
**Total**	12	12	**24**

SMH: St Mary's Hospital London
GNCH: Great North Children's Hospital Newcastle

Table 2. Summary of themes

**FACTORS ENCOURAGING USE OF POCTS**<br>*Advantages*<br>• Ease of use and rapid availability of test results (leading to faster decision making and enhanced patient flow)<br>• Improved clinical care (resulting from targeted antibiotic treatment, facilitated patient cohorting, closer monitoring, and improved communication with caregivers)<br>• Minimal test invasiveness<br>*Other factors*<br>• Training, experience, and medical specialty<br>• Guidelines
**FACTORS DISCOURAGING USE OF POCTS**<br>• Concerns over test accuracy<br>• Failure to resolve diagnostic uncertainty and alter clinical management<br>• Preferential reliance on clinical skills and acumen

For peer review only

343

Box 1. Factors encouraging the use of rapid POCTs

I. ADVANTAGES

Ease of use and rapid availability of test results

"If it's something the nurses could do really easily and quickly then it would help again, the flow and performance through the department. So let's say it takes 20 minutes to wee [make urinate] a child and it takes a specialist, a nurse, and the doctor...the nurse holding and etc, and you've got a really busy queue, and if you've got a really easy finger prick test that a nurse can do." [ED Paeds Consultant 1]

"Because it [POCTs in general] would just make things a lot quicker. Decision making a lot quicker. Being seen by doctors a lot quicker." [Nurse 3]

"So, you still might sit on the fence and want to admit and observe but it helps you just maybe make that decision a little bit quicker." [ED Paeds Consultant 1]

"So speed is probably the main advantage really and by virtue of being less people involved, it's a bit more accessible. You can also do them yourself, so you don't necessarily have to wait for other people to do the test." [Trainee 8]

Improved clinical management of febrile children

1. *Improved antibiotic stewardship*

"So that [urine dipstick] might change your choice of...not only whether you would give them antibiotics, but also what antibiotics you would give." [Trainee 9]

"I think that [POCTS in general] would help us with our overprescribing but also help reassure us that we are finding the ones that do need antibiotics and getting them to them [the children]." [ED Paeds Consultant 2]

"...I think bloods is probably one of the bigger ones...it just gives a much better indication as to whether or not we would treat with antibiotics or what...like...what kind of route you would go down..." [Nurse 3]

2. *Deciding cohorting and disposition of patient*

"The RSV test, we primarily…, are used for children who are being admitted. So we don't use it as a diagnostic test per se for children in the ED. But they use it for cohorting children if they are admitted to the ward." [ED Paeds Consultant 5]

"You know that kid where you are a little bit unsure, then the CRP come back a 5. And you were kinda thinking that it was probably ok, then it would influence a bit. Whereas the CRP came back at 60 or 100, then you be like, right they definitely need to come in (be

For peer review only - http://bmjopen.bmj.com/site/about/guidelines.xhtml

hospitalised) and that sort of stuff." [ED Paeds Consultant 1]

"With the urine [dipstick], I think we're determining whether they need other tests really and whether we need to screen further or look for other sources of infection." [ED Paeds Consultant 2]

3. *Closer monitoring for clinical changes*

"It's the repeat tests that I think that's the bigger advantage that they allow more easy repeat so you can assess the response to your treatment […] I think that is the single biggest advantage 'cause it gives you the quantification of the effect of your treatment." [ED Paeds Consultant 4]

"CRP (…), I think it is useful for monitoring if you've got a patient that is not following the usual course. Like if you got a drop in CRP and a raise again. I think it's very useful" [ED Paeds Consultant 2]

4. *Improved communication with caregivers*

"Parents find it quite stressful on how long like something bloods can take. They often come in and ask like how long it's going to be until my bloods are back. At least with the urine and the blood gas, you can kind of tell them fairly instantly. Like put their mind at rest and things." [Nurse 1]

"I think parents put great faith in technology. If you have a CRP that is negative, then it's negative. And if it's positive, then it's positive. So I think it's much more black and white way. And so, I think...it can be very convincing for them. So I think it can be helpful in that way." [Trainee 9]

"I think if somebody [parents] wanted antibiotics and I was sure they didn't [need] and the [rapid] CRP was there, I would be tempted, even though I thought know that it's not necessary evidence based to do a POCT, and say your CRP is only 6, you don't need antibiotics. [ED Paeds Consultant 5]

<u>Minimal test invasiveness</u>

"I might use that 'cause it's easy to do whereas I wouldn't want to do a proper venous blood test on a child because it's a painful procedure. A child would tolerate a little finger-prick, not like it, but they will tolerate it. Whereas a proper blood test involves lots of screaming, time, big needle, waiting around for hours." [Gen Paeds Consultant 2]

"Just thinking about, managing a sweaty toddler whose heart can be tachycardic who I think is probably going to be ok, and I'm struggling to get bloods, but if I could do a finger prick and I can get results in ten minutes, if it was a CRP of 100, I could take my time that we've got access and reassure the parents of why that was whereas if it was ten, I'd say well alright, we don't have to do bloods, we're just going to keep you here for a few hours and see how you are...so I think that would be helpful." [ED Paeds Consultant1]

For peer review only - http://bmjopen.bmj.com/site/about/guidelines.xhtml

II. OTHER FACTORS

Guidelines

"And so, I think we are very influenced by guidelines because we feel securities with guidelines. And so, to do a POCT, I would do it [if it is in the guidelines] because if I don't do it and it goes wrong, how will I defend myself. And essentially in fever, I already feel vulnerable." [Trainee 9]

"So I think with a POCT around fever, then you have to have a guideline about how you are gonna use the results. Because especially things like CRP, everyone is going to interpret CRP differently. (…) And so it would have to be a part of a guideline on how to interpret the results." [Trainee 9]

"If the guideline says to do...if there was a well-respected guideline in place for a particularly clinical scenario that says you should use this test, then of course, I would expect we would have to have a very good reason of why we aren't doing that test." [ED Paeds Consultant 4]

Training, experience, and specialty

"When you are a junior doctor you do a bit of more investigations and then you build a bit more of a profile and you get a feel for whether children are more unwell or not. [As a senior,] you probably tend to use less investigations." [ED Paeds Consultant 1]

"We would have a tendency in the A&E to do more investigations than a GP would...it's all about what you are used...what population that you see. So obviously, if you are an ID [infectious diseases] consultant, then you're seeing all the weird and wonderful so all the other stuff is filtered out by parents and GP and then by A&E and so the population that they see is different." [ED Paeds Consultant 1]

"If I had a point of care available for other things, I would use it. I would use a CRP, an FBC, I mean I would use everything if I can get it instantly from a finger-prick size sample. I know there is a feeling that probably when you get more senior, that you would sometimes you don't want the information from a blood test but at my level, I would..." [Trainee_5]

Box 2. Factors discouraging the use of rapid POCT

Concerns over test accuracy

For peer review only

For peer review only - http://bmjopen.bmj.com/site/about/guidelines.xhtml

"It may still be a viral infection and you have a borderline CRP and it may mean you have to go on and do further tests." [ED Paeds Consultant 2]

"There is a perception that they [POCTs] are less reliable, so often, there's a follow up with the formal lab equivalent…if you are shown a strong evidence base for accuracy of each POCT test, that would be critical for adoption." [Trainee 11]

"… if there was one test that could tell me if it wasn't bacterial infection, then yup. Fantastic. Something better than CRP. Yeah, it'd definitely be useful for that. If there was a viral diagnostic test that could tell me every single virus. Absolutely. Yeah. Definitely. I think if there were better, more evidence-based tests than yes I think we will definitely use them more.." [ED Paeds Consultant 5]

"I guess you'll have to do some research into that in terms of how to validate that…because sometimes I feel like a lot of the research…are actually done on…adults rather than children." [Trainee 7]

Failure to resolve diagnostic uncertainty and alter clinical management

"I don't think any of the POCT necessarily give you a diagnosis unless you've got urine dipstick that is positive for urine infection. Umm, so I don't think they really change your diagnosis as such." [Trainee 2]

"So I think that if I'm at the point where I want more information and I want to know what the CRP, I automatically want to observe them for a few hours, so it doesn't actually make that much of a difference to my working practice because I put less emphasis on the test than I do on the observations. ." [Trainee 9]

"I want something that's a binary. Cause I think what you might find with a lot of tests, it is a lot of grey. And it will give you an answer that, it could be a virus, it could be a bacteria; we're not really that sure. In which case it's useless as another blood test. Quicker, but otherwise no better. Cause that's what our CRP, our white cells show, and we are still left in the situation.." [Gen Paeds Consultant 2]

"I would be far more in favour of a test that could predict the likelihood of significant deterioration rather than one that could tell me whether something is viral or bacterial.." [ED Paeds Consultant 4]

"So, if you take an NPA [nasopharyngeal aspirate], which is how we test for viral respiratory panels here, you testing for 10 viruses. If you only take the RSV rapid test, you are only testing for RSV. And you still need to get a sample from the child. If you are getting a sample then why not just get the whole thing?" [Trainee 5]

"I guess the thing that I would want to know is that are they likely to change my management here and how…in a way that is going to be clinically significant. So, for example, a throat swab, if I'm going to prescribe antibiotics and the throat swab confirmed that then that is great, but that hasn't really changed anything." [ED Paeds Consultant 4]

For peer review only - http://bmjopen.bmj.com/site/about/guidelines.xhtml

Preferential reliance on clinical acumen

"Just the basic clinical skills that doctors...I think POCTs supplement what we do as doctors rather than replace it. I don't think they can be used in isolation." [ED Paeds Consultant 2]

"If I wasn't worried, I would trust my judgement because sometimes if you do further test to reassure a parent and it's not needed, you are putting a child through invasive tests, but you are potentially going to find something that might not be helpful in the bigger picture…Where I can, I like to manage on my clinical judgement in looking at the child and my observational skills." [ED Paeds Consultant 2]

"Like I said, I think basically a lot of what I do is based on history and examination...the essential skills that doctors need to know. I trust my history and examination quite a bit." [Trainee 10]

For peer review only

For peer review only - http://bmjopen.bmj.com/site/about/guidelines.xhtml

Supplementary materials

Supplement 1: Doctor’s topic guide

Supplement 2: Sampling matrix

Supplement 3: Initial coding lists

Supplement 4: Example of summary matrix

Supplement 5: Study protocol

Supplement 6: SRQR qualitative studies reporting checklist

Supplement 7: POCTs available at project site hospitals

Supplement 8: Measures in place to ensure safe and efficient use of POCTs in the two hospitals

For peer review only

Document version: 15.0
Document date 02/07/18
IRAS ID: 248723

Topic Guide (Clinicians): Perceptions of Hospital-based Healthcare Providers in England on the Use of Rapid Diagnostic Tests (RDTs)/Point of Care Tests (POCTs) in Febrile Infants, A Qualitative Study

Participant ID Number:	Gender: Male / Female
Date (DD/MM/YY):	

Patient Information Sheet & Informed Consent:

- Overview and Purpose of Study
- Aims of Interview and Expected Duration
- Who is Involved
- Why Participant's Involvement is Important, Advantages and Disadvantages
- What Will Happen to the Results of This Study
- Questions?
- Consent Form

Before I ask you further questions, here is what we will be covering in today's interview

- To explore the medical decision-making process of healthcare providers
- Focus on the availability and roles of RDTs/POCTs in the medical decision making process
- To explore advantages/disadvantages of RDTs/POCTs vs laboratory-based versions of the tests
- Implications of using RDTs/POCTs aside from the medical decision making process
- Facilitators/barriers to the wider adoption of POCTs in assessing febrile children
- To identify characteristics in future diagnostics in managing febrile children

For peer review only - http://bmjopen.bmj.com/site/about/guidelines.xhtml

Document version: 15.0
Document date 02/07/18
IRAS ID: 248723

Warm-up and Demographic Information: May I ask you some details about you and your background as a healthcare provider?

Job Title	
For how long have you been working in the hospital?	
Year of graduation from medical/nursing school	

Now I would like to ask you some questions about your experiences and perceptions managing febrile children

Aim	Topics & Prompts	Examples/Clarifications
To explore the medical decision-making process of healthcare providers	**How would you manage this patient?**<br>A 4-month-old infant presents with fever, asymptomatic otherwise, clinical examination unremarkable. The infant was inconsolable all morning, but currently settles with mum and feeding well, 3 non-bilious, milky vomits, vitals are within normal limits. Mum says he never vomits after feeds.<br><br>Prompts:<br>• What factors influence your decision to:<br>○ Discharge the patient home or admit into the hospital?<br>○ Use/not use antibiotics?<br>○ Use diagnostic tests? If yes, which tests? And why?	• *RDTs/POCTs are defined as diagnostic tests that can be completed at the patient's bedside and results are typically available within 15-20 minutes.*<br>• *We are focusing specifically on RDTs/POCTs used to investigate infections in children.*

For peer review only - http://bmjopen.bmj.com/site/about/guidelines.xhtml

Document version: 15.0
Document date 02/07/18
IRAS ID: 248723

Focusing down on the availability and role of RDTs/POCTs in the medical decision- making process	*I understand that in this hospital the RDTs/POCTs that are available are* (SHOW CARD)<br><br>**Are these RDTs/POCTs available in your A&E department for assessing febrile children?**<br><br>**Do you use any of these tests?**<br>**(if yes)**<br>• **In what cases/presentations would you use RDTs/POCTs?**<br>• **What exact purpose do they serve?**<br><br>• **Do you think these tests should be used for these purposes? Please elaborate.**<br><br>**How do RDTs/POCTs impact your initial working diagnosis?**<br>• **Do they change your initial working diagnosis?**<br>• **Are there circumstances where do they do not change your initial working diagnosis?**<br><br>**(if no)**<br>• **Why don't you use any RDTs/POCTs?**<br><br>**In some places where CRP RDTs/POCTs have been introduced, providers sometimes still prescribe antibiotics despite testing negative, what could be the reasons for this?**<br><br>**How would you rate yourself: are you a frequent user of diagnostics in general? Of RDTs/POCTs? Why is that?**<br><br>**Are there other RDTs/POCTs you would like to have available?**	• *For example: Are the test used to identify children who:*<br>○ *Need immediate care (triage)*<br>○ *Need additional confirmatory tests (screening)*<br>○ *Should be gathered (cohorted)*<br><br>• *For example, referring to the initial case scenario with a 4 month old febrile child and a rapid CRP test yielding 5 mg/L.*

For peer review only

Document version: 15.0
Document date 02/07/18
IRAS ID: 248723

To explore the perceived advantages and disadvantages of RDTs/POCTs and lab-based versions of the tests	**What do you consider to be the advantages and disadvantages about current RDTs/POCTs compared to their laboratory-based equivalents?**<br><br>**Are the tests used instead of equivalent lab tests (to decide, for example, on hospital admission or prescription of antibiotics?)**	• *Examples of RDTs/POCTs and lab equivalent:*<br>○ *Urine dipstick vs. microscopy*<br>• *What about:*<br>○ ***Need for training (is this factor a disadvantage?)***<br>○ ***Parental acceptance***<br>○ ***Route of obtaining sample***<br>○ *Time to get results*<br>○ *Costs*<br>○ *The fact that some clinicians find useful to have a delay between ordering the test and getting results, to allow watchful observation of the clinical evolution*

For peer review only

Document version: 15.0
Document date 02/07/18
IRAS ID: 248723

To explore wider implications of using RDTs/POCTs, aside from the medical decision-making process	**What are the expectations of parents when they present to hospital with a febrile child?**<br><br><u>Prompts</u><br>• With regards to:<br>○ the use of diagnostic tests? (e.g. do parents ask you for these tests?)<br>○ prescription of antibiotic?<br><br>• **How do these expectations impact on your decision to use RDTs/POCTs?**<br><br><u>Prompts</u><br>(ST. MARY'S ONLY – If participant says no)<br>• **If CRP RDTs/POCTs were available to you how would parental expectations affect your decision to use the tests?**<br><br>**Do you think there is a high risk of litigation in the UK? How does it affect your use of diagnostics and RDTs/POCTs?**<br><br>**Does the use of RDTs/POCTs have an impact on your relationship with parents and children?**<br><br><u>Prompts</u><br>• If CRP RDT/POCT were available to you, would it have an impact on your relationship with parents and children? If yes, please describe it to me.	

For peer review only - http://bmjopen.bmj.com/site/about/guidelines.xhtml

Document version: 15.0
Document date 02/07/18
IRAS ID: 248723

	**What other factors influence you on deciding whether to use RDTs/POCTs in children?**<br><br>**How do RDTs/POCTs fit within the consultation process?**<br><br>**What is the influence of other colleagues using the tests; are there influential colleagues? Who are they?**	• ***What about the role of guidelines?***<br>• ***What about previous adverse/memorable cases/difficult cases?***<br><br>*For example:*<br>• **Is the use of RDTs/POCTs disruptive in your routine clinical practice?**<br>• *What is the impact on patient flow?*<br>• *What happens whilst waiting for results?*<br>• *Who decides to request and administer the tests (e.g. nurses, doctors)?*<br>• *Who is responsible for acting on tests results?*

For peer review only

For peer review only - http://bmjopen.bmj.com/site/about/guidelines.xhtml

Document version: 15.0
Document date 02/07/18
IRAS ID: 248723

To identify facilitator(s) and barrier(s) to the wider adoption of POCTs for use in febrile children	**In your workplace, who decides on the introduction of new diagnostic tests?**<br><br>**What is or could be your role in deciding whether or not to introduce a new test?**<br><br>**What helps or hinders the adoption of additional POCTs in your work place?**<br><br>Prompts:<br>• Do you think barriers, if any, should be overcome?<br>• If yes, how could they be overcome?	• For example:<br>◦ A facilitator would be to have several devices so people do not need to queue up to use the POCT.<br>◦ A barrier would be if patients need to pay out of pocket for the test to be used<br>• What about accessibility, time pressure, accuracy of the POCTs? Staff factors? Cost factors? Hospital factors? Patient factors? Training? Physical Space? Other implications of introducing RDTs/POCTs in your workplace?
	**Some RCTs/POCTs are used more widely in other countries. Why are these used less in the UK despite being approved for use?**<br><br>**In your department, what is your perception of the clinical culture towards RDTs/POCT technology? Can you elaborate? By whom?**<br><br>**If you were given more autonomy in your practice at the A&E, do you think you would use more/less RDTs/POCTs? (Nurses)**<br><br>**Does your department have some autonomy from the hospital to decide on the introduction of RDTs/POCTS?**	• For example, CRP POCT is used more in the Netherlands.<br><br>• For example: open vs. conservative<br><br>• Autonomy: Orders for certain tests do not require a clinician to sign off on or administer.

For peer review only - http://bmjopen.bmj.com/site/about/guidelines.xhtml

Document version: 15.0
Document date 02/07/18
IRAS ID: 248723

To identify characteristics of future diagnostics for the management of febrile children	**Is there a need for novel diagnostic tests (POCTs or non-POCTs) that would help you in the management of febrile children?**<br><br>**For future RDTs/POCTs, what characteristics need to be improved to make you want to use them?**	• *What about:*<br>○ *Distinguishing between viral and bacterial infections*<br>○ *Predicting risk of developing severe disease*<br>○ *Differentiate between gram negative and gram positive infections*<br>○ *Identify bacterial species*<br>○ ***What is the maximum turnaround time for results you would find acceptable?***<br>○ ***What type of sample (blood, saliva, urine)?***

Closing: Is there anything else that you think is important about using diagnostic tests in the management of febrile children that we have not talked about?

- Summarize Covered Domains
- Any Other Questions/Concerns?
- Ensure participant has copies of patient information sheet and consent forms; provide any additional information as needed
- Thank interviewee for their input and participation.

For peer review only

Supplement 2: Sampling Matrix

**Healthcare workers**	**Hospital Site**		**Total**
	SMH, London	GNCH, Newcastle	
Nurses - Paediatric Emergency Department	2-3	2-3	4-6
Consultants - Paediatric Emergency Department	2-3	2-3	4-6
Consultants – General Paediatrics	2-3	2-3	4-6
Trainee doctors - Paediatrics*	5-6	5-6	10-12
**Total**	11-15	11-15	**22-30**

SMH: St Mary's Hospital London
GNCH: Great North Children's Hospital Newcastle

For peer review only

Supplement 3: Initial coding lists

Emerging theme	Codes	
	**Researcher: QL**	**Researcher: EL**
1. Trust in Clinical Judgement Over Diagnostics	RDTs not in isolation of acumen	Results to justify preliminary clinical judgements
	clinical acumen valued	Clinical examination/impression still more trusted
	assessment-dependent decision	Severity of clinical symptoms & signs
	Trust in clinical judgement	Decision to treat still dependent on clinical presentation
	Importance of examination and presentation	
	Tests and observations as clinical tools	
2. Perceived limitations of current POCTs	Worry for "grey" results	Clinical examination/impression still more trusted
	Concerns for interpreting results	CRP lag in elevation
	Time-consuming to obtain sample (i.e., urine dip)	Decision to treat still dependent on clinical presentation
	Machine need calibration	Results ambiguous/contradictory to initial impression
	Repeating test for ambiguous results	Difficult to interpret/use
	Cycle of repeat testing	"Grey areas", intermediate values
	Machine breaks down	Does not give overall diagnostic assessment of patient's condition
	Waiting for results	Forms "part of the picture" only
	Losing sample	
	Forgetting to input results	
	Concerns for falsely reassuring parents	
	'Treat the person, not the number'	
	Need for clinical picture (whole picture)	
3. Implementation of POCTs		
3a. Facilitators to introduction	Need clear guidelines for patient management	Increase acceptability for parents/child
	Need for evidence	
	Staff Buy-In	
	Need for staff education	
	Need for staff awareness	
	Need for training	

For peer review only - http://bmjopen.bmj.com/site/about/guidelines.xhtml

	Need training for test interpretation	
	Expanding nurses' roles	
	Nurse autonomy for RDTs	
	Need for nurse-led RDTs	
	RDTs for first point of patient contact	
	Need training to run test	
3b. Barriers to introduction	Fear of overreliance	Not actually that much faster than labs
	Overuse due to ease	Not as much information as labs
	False Positives	Results not as reliable/accurate as labs
	Fear of Overtreating	Over-reliance/use of POCTs
	Over-reassurance	False reassurances for clinicians and parents
	Fear use will undermine clinical judgement	Deterioration of clinical skills and judgement
	Concern for test quality control	Potential for overtreatment of patients
	High Staff turn-over	
	Difficult to coordinate training	
4. Current Roles of POCTs		
4a. Clinical management/Diagnostic Roles	Quick Rule-Out Tool (urine dip)	Used to monitor progress/response to treatment
	Aid antibiotic decision	Results clearly indicate potential diagnosis
	Prevent antibiotic misuse	Results to justify preliminary clinical judgements
	Reassurance for holding off antibiotics	Results support clinician's judgement
	Aid Clinicians' Stewardship Role	
	Prevent rapid deterioration	
	Aid Justification of antibiotics (urine dip)	
4b. Hospital Logistics/Practical Roles	Speediness (in general)	Reduce patient waiting times
	Speediness of results	Allows faster clinical decision making
	Speediness of decision-making	Avoids sampling inconveniences of lab-tests
	Quicker admission and discharge time	Patient cohorting to appropriate beds/cubicles
	Alleviate A&E pressure	
	Less wait time for parents	
	Reduce observation period	
	Quick status check in wards (blood gas, potential for CRP)	
	Quick bed allocation (cohorting) (RSV)	
4c. Parent-clinician communication role	Reassurance for Parents	Improves communication between parents and doctors
	Parental expectations of diagnosis and treatment	Parents want evidence from "a test"
	Aid in explaining decision-making	Results reduce parental anxiety

For peer review only

	Parental anxiety may influence RDT usage	When don't parents trust a clinician's judgement
	RDTs role to decrease parental anxiety	Parents do not care what test is used
	Address parents' "fever-phobia"	
	RDT as reassurance	
	RDT use decision: a 'middle ground'	
	Decisions based on collaboration	
5. Determinants of Current POCT Usage	Parental expectations of diagnosis and treatment	Clinical decisions not influenced by fear of litigation
	Parental anxiety may influence RDT usage	Clinical decisions influenced by fear of litigation
	Decisions based on collaboration	Clinical decisions not influenced by parental expectations
	RDT use decision: a 'middle ground'	Clinical decisions influenced by parental expectations
	Colleague's Influence	Want evidence from "a test"
	Junior doctors Use More RDTs	Parental Concerns
	Decrease in frequency with seniority (doctors)	Young age of patient
	Tools for less experienced (doctors)	Unclear infectious foci
	Increase in frequency with autonomy (nurse)	
6. Characteristics of Future POCTs		
6a. Performance properties	Need to know reliability and specificity	More information from one POCT
	Importance of PPV and NPV	Results should make a difference in clinical course/management
	Need for test to change management	Should match gold-standard of lab-tests
	Need for decreasing ambiguity	Provides additional/different information from lab tests
6b. Practical use properties	Easy to act on binary results	Easy to interpret results
		Positive or negative results
		Numeric thresholds are ambiguous
		Need for analysers
		Minimal amounts of sample needed
		Painless/non-invasive for child
		Not time consuming to obtain
		Easier to get than lab-tests

For peer review only

Supplement 4: Example of summary matrix for the emergent theme "Trust in clinical acumen"

Participant	Initial coding	Sub-theme	Quote
ED_C2N	Assessment based decision	Patient management is based more on clinical findings than on diagnostic tests	To be honest with you, ED we really aren't going to get any diagnostic tests back. If the child is unwell, the child is going to be admitted. I might send some baseline inflammatory markers, so full blood count, CRP, Basic electrolytes, obviously blood cultures if their child is very febrile or if I'm concerned that they are shocked, urine sample. But beyond that, most of my management is going to be clinical grounds rather than treating the test result.
ED_N3N	Assessment based decision	Patient management is based more on clinical findings than on diagnostic tests	Even if the CRP was 5, that wouldn't mean you wouldn't then go on and do a septic screen. So it very much depends on the baby and how he looks and how he changes because...they change very very quickly.
ED_C1L	Assessment-based decision, RDT not in isolation	Diagnostic tests are just a complement of clinical assessment, they should not be used in isolation	So just the importance of making sure that it's still all about clinical assessment, and test are just an additional thing to that, that are really valuable and helpful.
ED_C1L	clinical acumen valued	Diagnostic tests are just a complement of clinical assessment, they should not be used in isolation	And inflammatory markers are a guide, but you know, we can get caught out where inflammatory markers can be low, and the child can still be very unwell. So it depends on the whole clinical acumen thing.
ED_C2L	Clinical acumen valued	?	I think you kind of go off clinically on what the case looks like.
ED_J2L	Clinical acumen valued	Patient management is based more on clinical findings than on diagnostic tests	...I'd prefer to be clinically led cuz if I was saying there was a number, CRP or the white count, which I may worry about sepsis. Then it could be argued that shouldn't you be worried even if those numbers are normal given that we know that the sensitive is particularly early in serious infection.
ED_N3N	Clinical acumen valued	Patient management is based more on clinical findings than on diagnostic tests	To obviously get the cultures and some things like that, and make sure we start them on sensitive to the particular infection they had. But, it would be a clinical determination on how unwell the child was and whether or not we were worried about them. It might put us a little direction on what the focus of the infection.
ED_J1N	Importance of examination and presentation	Patient management is based more on clinical findings than on diagnostic tests	I suppose there is always what you are seeing...you shouldn't be treating the numbers...you should be treating the patient so there is always a discrepancy between what you are seeing in your patient and your results. [J1N]
ED_C1L	Importance of examination and presentation	Patient management is based more on clinical findings than on diagnostic tests	Yeah so, first of all, it would be on the history and examination and what he looks like in front of me.
ED_N1L	Importance of examination and presentation	Sicker children can be identified clinically	And, it's usually, you're more aware of those children at triage, because usually the sicker children are having very abnormal obs. You can their respiratory efforts are a lot more increased. So you can usually differentiate between those children, roughly know that child...you know...this baby may need watching for 24 hours in our CDU, or it may need admission to the ward. You can usually differentiate between those two.
ED_J1N	Importance of examination and presentation	Patient management is based more on clinical findings than on diagnostic tests	Then I'd concentrate on my history and examination more than any other tests at that point.

ED_J1N	Importance of examination and presentation	Patient management is based more on clinical findings than on diagnostic tests	I think I always base the majority of my differential diagnosis on the history and examination really in a child like that.
ED_J1N	Importance of examination and presentation	Patient management is based more on clinical findings than on diagnostic tests	like I said, I think basically a lot of what I do is based on history and examination...the essential skills that doctors need to know. I trust my history and examination quite a bit.
ED_C1L	RDT not used in Isolation	Diagnostic tests are just a complement of clinical assessment, they should not be used in isolation	So I think clinical picture is still the most valuable tool and we have to make sure that people are trained in recognition of the unwell child, and use tests to augment them.
ED_C2L	RDT not used in Isolation	Diagnostic tests are just a complement of clinical assessment, they should not be used in isolation	people try to see CRP so they would use it as a kind of tracking rather than a stand alone. So, to be honest, I've not seen that many people treating anybody based on the CRP without some kind of clinical reasoning for doing so. If the patient looks unwell, you'd treat them.
ED_J1N	RDT not used in Isolation	Diagnostic tests are just a complement of clinical assessment, they should not be used in isolation	But I think if we do bloods, it is always together with history and examination just to aid your decision making. I don't think it makes your decision.
ED_C1N	RDT not used in Isolation	Diagnostic tests are just a complement of clinical assessment, they should not be used in isolation	Umm...yeah, I think that they can't be taken in isolation and I think that as long as they are used along with clinical judgement and after their assessment of the patient, taking into account all of the other factors such as their observations, their examination, the history that you've got, the parental concern, I think they are all very useful to make a clinical decision.
ED_C1N	RDT not used in Isolation	Diagnostic tests are just a complement of clinical assessment, they should not be used in isolation	Just the basic clinical skills that doctors...I think POCTs supplement what we do as doctors rather than replace it. I don't think they can be used in isolation.
ED_C1L	RDTs not used in Isolation	Diagnostic tests are just a complement of clinical assessment, they should not be used in isolation	I think that they can't be taken in isolation. I think that as long as they are used along with clinical judgement. And after their assessment of the patient. And taking into account all of the other factors such as their observations, their examination, the history that you've got, the parental concern. I think they are all very useful to make a clinical decision.
ED_C1L	RDTs not used in Isolation	Diagnostic tests are an alternative or complement of clinical assessment	So I think, you know, that's great that they are easy to do and that we get results fairly quickly but to think of the use of them in the same context where we happen to actually bleed a child...so we can use our clinical acumen as well.
ED_N2N	RDTs not used in Isolation	Diagnostic tests are just a complement of clinical assessment, they should not be used in isolation	A clinical reason to use it or if you are concerned about the patient and how they look. Then, just do the tests that are necessary so that you know you've made the right decision.
ED_C1N	RDTs not used in Isolation	Diagnostic tests are just a complement of clinical assessment, they should not be used in isolation	I don't think disruptive. Umm...as long as it's taken into context and that it's not taken in isolation.
ED_C1L	Tests and Observations (paired) as clinical tools	Clinical observation is used as a diagnostic tool	But we use that a lot. Any child with a fever without a very good focus. And even sometimes if they've got a respiratory tract focus, you might still want to make sure that we are...and if its clear. Often it's good to wait for them to wait for them to wee anyway. If there's a story of...umm....reduced fluid intake.

For peer review only

ED_C1L	Tests and Observations (paired) as clinical tools	Clinical observation is used as a diagnostic tool	what the point of doing the test is, explain what the results are, and say it's kind of here or there. And then, if the...again, if you are clinically worried, you would keep them for observation but maybe not treat.
ED_C1L	Tests and Observations (paired) as clinical tools	Clinical observation is used as a diagnostic tool	I can't remember exactly but maybe the CRP was 20-30. And then something else. But they were persistently tachycardic, but the urine was clear. And we do a chest x-ray and that was clear. And there was no meningitis or anything like that. And, the child didn't look septic or unwell. Just tachycardic. And in the end, we just observed them without treating them, and the tachycardic settled and then they went home. So, sometimes with pediatrics particularly, think, you just need a bit more time and such things.
ED_C1L	Tests and Observations (paired) as clinical tools	Clinical observation is used as a diagnostic tool	Regardless of how obvious the actual diagnosis is. So I think to help augment it, that would be really valuable though cuz it would help you maybe filter out some of those ambic [ambulatory] kids that you would admit but could actually could go home. You know, but then, actually, period observation is a good active treatment I think in pediatrics. I
ED_C2L	Tests and Observations (paired) as clinical tools	Clinical observation is used as a diagnostic tool	RDTs get people moving through quicker. Yeah. But I think in peds, we accept that our flow is often a bit disruptive. Cuz in pediatrics especially, there is quite a big role for a period of observation especially in febrile children.
ED_J1L	Tests and Observations (paired) as clinical tools	Clinical assessment and observation are more valuable than CRP	So I think that if I'm at the point where I want more information and I want to know what the CRP, I automatically want to observe them for a few hours, so it doesn't actually make that much of a difference to my working practice because I put less emphasis on the test than I do on the observations.
ED_J1N	Tests and Observations (paired) as clinical tools	Clinical observation is used as a diagnostic tool	So the child is usually just waiting with the parents. Sometimes it's useful because we can just observe them and see how they go for their fever or if we are waiting for anything else which is the urine sample or a fluid challenge. So sometimes, it doesn't affect the length of stay in the hospital cuz we were observing them anyway.
ED_C1N	Tests and Observations (paired) as clinical tools	Diagnostic tests are an alternative or complement of clinical assessment	So I think having useful tests and then documentation of how they have presented to you is essential. And whether that's the POCTs or observations and clinically document them in the notes the findings and what you've discussed.
ED_C2L	Trust in clinical judgement	Patient management is based more on clinical findings than on diagnostic tests	if they didn't, it would go more on how well they looked clinically.
ED_C1N	Trust in clinical judgement	Patient management is based more on clinical findings than on diagnostic tests	Where I can, I like to manage on my clinical judgement in looking at the child and my observational skills.
ED_C2L	Trust in clinical judgement	Patient management is based more on clinical findings than on diagnostic tests	But again, I don't find CRP that useful in that way, cuz I found that most people would go clinically on how the patient is like.
ED_J1N	Trust in clinical judgement	Patient management is based more on clinical findings than on diagnostic tests	I think you should base it on your clinical skills rather than investigating.

For peer review only

ED_C1N	Trust in clinical judgement	Patient management is based more on clinical findings than on diagnostic tests	I think it's good to use the test, but I think it doesn't replace clinical judgement and thorough clinical assessment.
ED_C1N	Trust in clinical judgement	Patient management is based more on clinical findings than on diagnostic tests	I think RDTs if you're concerned as a clinician, if even a test negative you should act on that concern.
ED_C1L	Trust in clinical judgement, RDT not used in isolation	Diagnostic tests are just a complement of clinical assessment, they should not be used in isolation	I guess, I try to base my differential diagnosis on clinical...on history and examination first. And if it's really clear, the POCTs augment what your management plan is going to do anyway. Cuz you're just going to treat based on that anyway.
ED_C1L	Trust in clinical judgement, RDT not used in isolation	Patient management is based more on clinical findings than on diagnostic tests	I guess anyway you go with your clinical picture,
CON1N	Clinical examination/impression still more trusted	Patient management is based more on clinical findings than on diagnostic tests	I think if we came back to that one, even if the CRP was low on POCT, I think I would still act because I know that, I know all the time that a lab CRP can be done and they can be low and we still get signs of an infection
SHO1L	Clinical examination/impression still more trusted	Clinical assessment and observation are more valuable than a POCT	And actually, I think the feeling is that having two hours with the patient is often more valuable than the result that you get back because you just get the time to see things for a bit longer, and just assess that baby or that child a few more times. […] I don't want to be able to see a child and send them home in 10 minutes, because I'll probably miss something. I'd much prefer if I saw a child and saw them for two hours, and pop my head in a few times and then have some blood results back that reassures me to let the child go home.
REG2N	Clinical examination/impression still more trusted	Clinical assessment and observation are more valuable than a POCT	I don't think so. Ummm, I think probably because on what I've touched on before. So I think the febrile children that you see fall into a few categories. So I think you get the children who are febrile and there is, you find a focus to their infection and based on that it's, it's normally, you're normally able to say if it's going to be more viral and kind of self-limiting, or this is more likely to be bacterial and you'd give the appropriate antibiotics depending on where the infection was. Then you get children who you can't find a focus but they look well. And if you've got a child who exams well with normal obs, then I would tend to do a period of observations, or if they look completely well, home with clear safety net advice for the parents to bring back if they became more unwell. Or I think you get the children who look sick and look septic, and in those children, you are going to treat with antibiotics. I was thinking, I couldn't think of like specific groups of children where a POCT would like really help you, help me decide on whether to give antibiotics or not.

Protocol version number: 6.0
Protocol version date: 24/05/18
IRAS number: 248723

For peer review only

Study Protocol: Perceptions of Hospital-based Healthcare Providers in England on the Use of Point of Care Tests (POCTs) in Febrile Children, A Qualitative Study

Research project carried out as part of an MSc in Public Health

This protocol has regard for the HRA guidance and order of content

For peer review only - http://bmjopen.bmj.com/site/about/guidelines.xhtml

Protocol version number: 6.0
Protocol version date: 24/05/18
IRAS number: 248723

Full Title: Perceptions of Hospital-based Healthcare Providers in England on the Use of Point of Care Tests (POCTs) in Febrile Children, A Qualitative Study

Investigators:

Principal Investigator and supervisor	Dr Shunmay Yeung, London School of Hygiene and Tropical Medicine, UK.
MSc candidates	Edmond Li, London School of Hygiene and Tropical Medicine, UK.<br><br>Queena Luu, London School of Hygiene and Tropical Medicine, UK.
Co-supervisor	Dr Juan Emmanuel Dewez, London School of Hygiene and Tropical Medicine, UK.<br><br>Email: Manuel.Dewez@lshtm.ac.uk
Co-investigators	Dr Marieke Emonts<br><br>Newcastle upon Tyne Hospitals Foundation NHS Trust, and Institute of Cellular Medicine, Newcastle University, Newcastle upon Tyne, UK.<br><br>Dr Ian Maconochie,<br><br>St Mary's Hospital, Imperial College Healthcare NHS Trust, London, UK.<br><br>Dr Ruud Nijman,<br><br>St Mary's Hospital, Imperial College Healthcare NHS Trust, London, UK.
Sponsor	London School of Hygiene and Tropical Medicine

For peer review only

Protocol version number: 6.0
Protocol version date: 24/05/18
IRAS number: 248723

Study summary

Study Title	Perceptions of Hospital-based Healthcare Providers in England on the Use of Point of Care Tests (POCTs) in Febrile Children, A Qualitative Study
Short title	Healthcare Providers Perceptions on POC Testing in Febrile Children
Study Design	Qualitative in-depth interviews
Study Participants	Emergency department doctors and nurse, hospital paediatricians from two English hospitals
Planned Size of Sample (if applicable)	Around 30 overall (15 per site)
Planned Study Period	June- 2018
Research Question/Aim(s)	The aim of this project is to explore the perceptions of hospital-based healthcare providers about the use of POCTs in febrile children in England.

For peer review only

Protocol version number: 6.0
Protocol version date: 24/05/18
IRAS number: 248723

Table of Contents

1 Background 6

2 Rationale 7

3 Theoretical Framework 8

4 Research Aim 8

5 Objectives 8

6 Outcomes 8

7 Study Design, Methods, Data Collection and Analysis 9
- **7.1 Study Design 9**
- **7.2 Data Collection 9**
- **7.3 Data Analysis 9**

8 Study Setting 9

9 Eligibility Criterion 10
- **9.1 Inclusion Criterion 10**
- **9.2 Exclusion Criterion 10**

10 Sample Size 10
- **10.1 Sampling Technique 10**
- **10.2 Recruitment 10**
  - 10.2.1 Sample Identification 10

11 Consent 11

12 Ethical and Regulatory Considerations 11
- **12.1 Assessment and Management of Risk 11**

13 Research Ethics Committee (REC) and Other Regulatory Review & Reports 12

14 Peer Review 12

15 Data Protection and Participant Confidentiality 12

16 Access to the Final Study Dataset 12

17 Dissemination Policy 13

18 Authorship Eligibility Guidelines 13

19 References 14

20 Appendix 15

For peer review only

For peer review only - http://bmjopen.bmj.com/site/about/guidelines.xhtml

Table of Appendices

Appendix 1: Sampling Matrix 15

ppendix 2: Gantt Chart of Study Timeline 16

For peer review only

Protocol version number: 6.0
Protocol version date: 24/05/18
IRAS number: 248723

1 Background

Correctly diagnosing and managing a febrile child presenting to hospital, can often prove a challenging task even for seasoned clinicians. Deriving whether the aetiology is bacterial or viral in nature, and whether the patient will deteriorate in the next 24-48 hours can be challenging despite thorough clinical examination and laboratory tests. Clinical examination can be hindered by the lack of specific signs, while laboratory-based diagnostics, such as blood cultures may take 48 hours or more before results are available (Craig *et al.*, 2010; Van den Bruel *et al.*, 2010).

This diagnostic uncertainty typically prompts clinicians to pre-emptively prescribe antibiotics for fear of missing severe but treatable bacterial infections (Lucas *et al.*, 2015). The resulting overuse of antibiotics is likely to be one of the factors contributing to anti-microbial resistance (AMR) (World Health Organization (WHO), 2015). Moreover, clinicians often admit febrile children (particularly young infants) to rule out severe infections. During the hospitalisation, children are clinically monitored to detect any potential deterioration, and undergo several diagnostic tests (Massin *et al.*, 2006). This includes blood tests, X-rays, blood cultures, or cultures from normally sterile biological fluids, such as cerebrospinal fluid. The latter implies invasive procedures, such as lumbar puncture. In parallel, children are prescribed broad-spectrum antibiotics to ensure potential severe bacterial infections are not left untreated. It is estimated that up to 20% of paediatric hospitalisations are to rule out severe infections in febrile children (Massin *et al.*, 2006). Most of them will eventually not have evidence of severe infection, but it takes up to 48 hours for the results of some of the tests to become available and confirm the trivial nature of fever. This approach is an important burden to health services in terms of resource use. It is also an important source of anxiety and discomfort for children and their families as the child is exposed to the risk of nosocomial infections while hospitalised, and to the invasiveness of certain tests.

Currently, the WHO Global Action Plan on AMR recommends using “effective, rapid, low-cost diagnostic tools for guiding optimal use of antibiotics in human and animal medicine, and that such tools should be easily integrated into clinical practices” to address developing AMR (World Health Organization (WHO), 2015). Point of care tests (POCTs) belong to this classification of investigative diagnostics. They can be easily performed in the patient’s immediate surroundings and produce results within minutes to aid clinical decision-making and guide patient management. The diagnostic uses of POCTs can range from identifying specific pathogens such as Group A streptococcus (Stewart *et al.*, 2014) to trying distinguishing between bacterial and viral sources of infection by the host’s non-specific inflammatory response to infection by measuring C-reactive protein (CRP) or procolcitonin (PCT) (Van Den Bruel *et al.*, 2011). This latter category of POCT is especially useful in febrile patients without a clear source of infection.

However, patient management decisions do not always correspond directly to the availability and use of POCTs. For example, *Burchett et. al* demonstrated that while POCTs to diagnose malaria are available, clinicians often still prescribe anti-malarial medications regardless of negative results (Burchett *et al.*, 2017). There are a number of reasons for this, including mistrust of tests results, a reluctance to make the substantial shift from reliance on clinical judgement and perceptions about patients and carers expectations. Hence, understanding how

For peer review only - http://bmjopen.bmj.com/site/about/guidelines.xhtml

Protocol version number: 6.0
Protocol version date: 24/05/18
IRAS number: 248723

clinicians perceive POCTs, in addition to how POCTs are used, is critical to promote their appropriate integration into routine clinical practice.

Existing studies regarding POCT use have been conducted primarily in the outpatient/General Practice (GP) setting or for adult patients (Jones *et al.*, 2013; Hardy *et al.*, 2017). Thus, it is unclear whether the results derived are translatable to the paediatric context. In addition to this, though study results showed GPs were largely positive regarding POCT usage; this was not without concerns. These concerns included POCTs' diagnostic reliability and accuracy, the potential for over-reliance in guiding treatment, and cost-effectiveness compared to traditional laboratory diagnostics. *Schot et. al's* study showed that while GPs in the Netherlands were enthusiastic about POCTs in adult practice, they were reluctant to do the same in children due to perceiving them to be a vulnerable patient group and subsequently prescribed antibiotics regardless (Schot *et al.*, 2017). *Van de Bruel et. al* demonstrated that GPs in the UK were happy to use CRP POCTs in children. However, this study was limited by the small sample size as well as its focus primarily in the outpatient setting. Hence, the findings may not be applicable to the in-patient/hospitalist setting.

In terms of the perceptions of hospital-based paediatricians about the use of POCTs, *Bustinduy et. al* conducted an exploratory study in three hospitals in London to explore the feasibility of integrating POCTs in the clinical management of two paediatric wards (Bustinduy *et al.*, 2017). The study showed that paediatricians had contrasting views: they appreciated the rapidity of getting results which helped in bed management and cohorting patients, and in the potential decrease in antibiotics use and length of stay. However, they were unsure about the accuracy of POCTs, and reported the need for additional training, space to host the material and costs as potential barriers to implementation. This study shows the multifaceted dimensions of POCTs use in clinical practice. However, given its exploratory nature, the study does not provide an in depth understanding of these issues. To the best of our knowledge, there are no other studies that explored the views of hospital-based healthcare providers.

The aim of this project is hence, to explore in depth the perceptions of hospital-based healthcare providers in the UK about the use of POCTs in febrile children, through qualitative interviews.

2 Rationale

Better implementation of current and future POCTs into routine clinical practice could result in better antimicrobial stewardship, more efficient allocation of medical resources, and reduce diagnostic uncertainty/misdiagnosis of severe bacterial infections (SBI) in febrile children.

However, the potential impact will depend on how they are taken up by clinicians in different clinical settings. Overall, there is lack of evidence pertaining to the perceptions of hospital-based healthcare providers using POCTs for assessing febrile children in the hospital setting. Hence, a qualitative study using in-depth interviews to explore how hospital-based healthcare providers perceive the actual or potential use of POCTs in febrile children will be useful in

For peer review only - http://bmjopen.bmj.com/site/about/guidelines.xhtml

Protocol version number: 6.0
Protocol version date: 24/05/18
IRAS number: 248723

informing the introduction of current and future POCTs for the management of children in England.

3 Theoretical Framework

This study has an applied rather than a theory-generating focus (i.e. oriented towards informing policy recommendations and implementation rather than creating new theories). From an ontological perspective, this study is close to the "critical realism" school of thought, which believes that reality exists independently of those who live in it, but acknowledges that reality is complex and multifaceted, and thus recommends interpreting the perceptions of individuals and how they make sense of their experiences of a given reality (e.g. the provision of medical care to febrile children) to develop an in-depth understanding of it (Ritchie *et al.*, n.d.).

From an epistemological point of view, the study will use both inductive and deductive approaches to generate knowledge. A deductive approach based on current knowledge about the use of POCTs in adults will be used to design the study materials (e.g. topic guides). However, during fieldwork and the iterative analysis process, an inductive approach will be combined to the deductive approach, to ensure ideas and themes that were not initially identified are also captured. Finally, during the final stages of analysis, we will integrate the findings of the study in the context of existing knowledge about the topic.

4 Research Aim

The aim of this project is to explore the perceptions of hospital-based healthcare providers (i.e. paediatricians, emergency department physicians, and nurses) about the use of POCTs in febrile children in England.

5 Objectives

1. To explore the role(s) POCTs play or may play in the clinical decision-making process of hospital-based healthcare providers.
2. To explore the role(s) POCTs play or may play aside from the clinical decision-making process (e.g. on the patient-physician relationship)
3. To explore the perceived advantages and disadvantages of POCTs and lab-based versions of the tests
4. To identify ideal characteristics of future POCTs for the management of febrile children
5. To identify facilitator(s) and barrier(s) to the wider adoption of POCTs by hospital-based healthcare providers.

6 Outcomes

The potential broad outcomes for this study include themes and concepts that are important to hospital-based healthcare providers that will allow an in-depth understanding of the utilization of POCTs for diagnosing febrile children in practice.

For peer review only - http://bmjopen.bmj.com/site/about/guidelines.xhtml

Protocol version number: 6.0
Protocol version date: 24/05/18
IRAS number: 248723

7 Study Design, Methods, Data Collection and Analysis

7.1 Study Design

Qualitative methodology will be used in this study. This method is appropriate for the given aims of increasing understanding as little is known about hospital-based physicians' and nurses' perception on current and potential use of rapid diagnostic tests in clinical-decision making for febrile children. The use of qualitative methods allows for a greater understanding of the subject matter from individuals with a specialist role (Ritchie *et al.*, n.d.).

7.2 Data Collection

In-depth interviews will be conducted on a one to one basis between the primary researchers (the MSc students) and the interviewee (hospital-based healthcare provider). The interview will be guided by a topic guide designed to explore the different aspects of POCT use in clinical practice as described in the study objectives. The topic guide will be developed by the primary researchers with the support of supervisors and based on previous research published in the medical literature. These interviews will last approximately 45 minutes in duration and will be audio recorded with an encrypted recording device for transcription afterwards.

7.3 Data Analysis

Each interview recording will be transcribed and analysed by the interviewer (i.e., the MSc student).

Thematic analysis will be done iteratively to identify topics which will be progressively integrated into higher order key themes through the following process: familiarization with the data, the construction of an initial thematic framework, the indexation and coding of the data using the initial framework, the gathering of the indexed and coded data into initial themes, and the revision for coherence of the initial themes. These steps will be followed by a process of describing and refining the initial themes into final themes, and will end with the explanation/interpretation of the data (Bazeley, 2013; Ritchie *et al.*, n.d.).

The timeline of the study is available in appendix (Appendix 2).

8 Study Setting

The study is to be completed at two hospitals: one in Newcastle (Great North Children's Hospital), and one in London (St. Mary's Paddington Hospital).

Great North Children's Hospital provides care to a mixed rural and urban population, while St. Mary's Paddington Hospital provides care to an inner-city urban population. These differences may have an impact on the patient-provider relationship and thus on the use of diagnostic tests.

For peer review only - http://bmjopen.bmj.com/site/about/guidelines.xhtml

Protocol version number: 6.0
Protocol version date: 24/05/18
IRAS number: 248723

9 Eligibility Criterion

As this study is interested in the perceptions and attitudes of in-hospital healthcare providers (i.e., paediatricians, emergency department physicians, and nurses) from the two different hospital mentioned above, they will be included.

9.1 Inclusion Criterion

- Hospital-based healthcare providers, specifically:
  - Paediatricians (i.e., generalist and sub-specialist[1]) and emergency department physicians
  - Emergency department based paediatric site practitioner nurses
- Participants working in the Great Northern Children's Hospital, Newcastle, or St. Mary's Hospital, London, England, UK.
- Participants will have different levels of expertise and seniority (trainees and consultants)

9.2 Exclusion Criterion

- Healthcare providers who are not based in the Great Northern Children's Hospital, Newcastle, or St. Mary's Hospital, London.
- Healthcare providers who do not asses acutely ill children in their routine practice
- Medical and nursing students

10 Sample Size

The sample size for this study is approximately 30 healthcare providers (Appendix 1). The researchers expect to be able to conduct this number of interviews during the time allocated for the project. This sample size is expected to be enough to reach data saturation (i.e., the point of data collection where there are no new themes emerging from the collected data).

Small numbers are commonly used for qualitative research studies with semi-structured, in-depth interviews (Ritchie *et al.*, n.d.; Flick, n.d.) because the information yielded are in-depth and rich in detail which need to be balanced against resource and time constraints (Ritchie *et al.*, n.d.). This sample size is also reasonable when compared to the sample sizes other studies with a similar topic of focus (Van Den Bruel *et al.*, 2016; Schot *et al.*, 2017; Jones *et al.*, 2013; Hardy *et al.*, 2017).

10.1 Sampling Technique

Purposive (criterion-based) sampling will be utilized to select physicians and nurses from two NHS hospitals mentioned above. Purposive sample will be utilized to capture a diversity of healthcare provider perspectives with stratification by the hospital location (Great Northern Children's Hospital, Newcastle, or St. Mary's Hospital, London, England, UK.) and for physicians, the level of clinical experience (specialist registrar or consultant) to gain an understanding of different viewpoints within the context of NHS hospitals.

[1] For example, paediatricians specialised in Infectious diseases, oncology, and intensive care.

For peer review only - http://bmjopen.bmj.com/site/about/guidelines.xhtml

Protocol version number: 6.0
Protocol version date: 24/05/18
IRAS number: 248723

10.2 Recruitment

10.2.1 Sample Identification

Supervisor(s) and local co-investigators will use the sampling matrix (Appendix 1) to identify potential participants. Feasible recruitment strategies for each study location will be discussed and reviewed with local co-investigators prior to implementation.

One suggestion may be the following: Supervisor(s) will contact the heads of the departments (General paediatric department and emergency department) in each hospital via email to introduce the study and seek assistance in identifying participants that meet the inclusion criteria. Potential participants will be contacted via e-mail by the heads of the departments and provided with a participant information sheet (PIS), which describes the study aim (see below). Once potential participants confirm their interest in the study, an interview date, time, and location will be agreed upon.

11 Consent

The researchers (i.e., the MSc students) will be responsible for obtaining written informed consent in-person prior to interview. First, the researcher will read a participant information sheet to verbally explain the aims and the nature of study. During this time, potential participants will be given the opportunity to ask questions regarding the study and their role. Potential participants will be given a copy of the information sheet for their own records and reference. Afterwards, the potential participant will be supplied with a written consent form. The form will ask about consenting to partaking in the study, for the interview to be audio-recorded, for direct quotes to be used in report, and for allowing the data gathered to be used in the current project and in future research. Potential participants will be given as much time as needed to think about partaking to the study. If the participant consents, they will sign and date two copies of the consent form. The researcher will also sign and date the two copies. The participant and researcher will each keep a copy of the consent form. If the participant consents to partaking in the study but not to be audio-recorded, the research will take written notes of the discussion. If the participant consents to partaking in the study but not allowing the data to be used in future research, this is noted on the informed consent form.

12 Ethical and Regulatory Considerations

12.1 Assessment and Management of Risk

There should be no physical risks to the participants. There may be a very small risk of emotional discomfort due to the sharing of past difficult experiences and perceptions regarding clinical decision-making as well as use of point of care testing in the hospital setting. If there is emotional discomfort, the interview will be stopped, and their data will not be used in the analysis.

Moreover, participants might feel that the responses to the interview will be used to judge their clinical work performance. The researchers will clarify that the purpose of the interview is not to assess their quality of work as physicians and nurses, but rather, to understand their perceptions surrounding clinical decision-making and rapid diagnostic testing.

For peer review only

Protocol version number: 6.0
Protocol version date: 24/05/18
IRAS number: 248723

The inconvenience of taking time away from their routine work, in order to take part in the interview process, can also be a source of discomfort. Thus, prior to the interview, the researcher and supervisor(s) will communicate with participants to set up an interview day, time, and location to ensure that it is convenient for the participants. Additionally, a thorough preparation (e.g., availability of forms and the readiness of encrypted recording devices) will be ensured before each interview.

Potential participants will be told that participation is voluntary and can be withdrawal at any time. Additionally, the information gathered will be confidential (i.e., the information from the interview will be anonymized prior to data analysis).

13 Research Ethics Committee (REC) and Other Regulatory Review & Reports

Ethics approval is being sought from the London School of Hygiene and Tropical Medicine Ethics Review Board. Health Research Authority approval will be sought prior to the commencement of the study. NHS Ethics Board approval will not be necessary as the research does not involve patients or their carers, nor require access to clinical data.

14 Peer Review

This study will be reviewed by senior project supervisors who are experienced in qualitative paediatrics research. Throughout the study, they will be providing necessary feedback to the primary researcher. The project supervisors are currently also conducting research into understanding POCT use in children across different European countries. They are preparing quantitative surveys and in-depth case studies to investigate how POCTs are being used by healthcare providers to manage febrile children in different European countries. Their research, in addition to this study, is part of the PERFORM research consortium. This consortium consists of twelve European universities aimed at developing novel POCTs to help differentiate between bacterial and viral infection in febrile children (www. perform2020.eu).

15 Data Protection and Participant Confidentiality

Interviews will be conducted in a private meeting rooms in the two hospitals to ensure confidentiality of the information discussed.

Coded identifiers (i.e., assignment with unique ID codes) will be used in the transcripts in place of participant's names. ID codes will consist of two letters related to the study site, two letters the type of participant, and two numbers to the participant. The master list of the ID codes will be encrypted and stored separately. The researchers and supervisors will not use the list during data analysis. If any potential identifying factors are mentioned by the participant (e.g., names of people or venues), these will not be transcribed.. Participants will have the option, should they choose, to not have their words quoted in the reports.

All recorded and transcribed data will be stored securely in the London School of Hygiene and Tropical Medicine (LSHTM) server. Audio recordings will be immediately transferred to

For peer review only - http://bmjopen.bmj.com/site/about/guidelines.xhtml

encrypted laptops and uploaded to the LSHTM server using an encrypted connection. Data will be encrypted with an encryption software (VeraCrypt or FileVault). Audio-records will be deleted once they have been transcribed. Data will be shared between investigators (see page 2 for list of investigators) via encrypted emails.

All handwritten notes taken during the interview by the researchers will be stored securely in a locked cupboard and disposed at the end of the study.

16 Access to the Final Study Dataset

Only the researchers (MSc students) and investigators will have access to the data stored on the LSHTM encrypted servers. The data will be stored for up to ten years as per LSHTM data management protocols. The data will be available for future research upon request to the overall project supervisor, Dr. Shunmay Yeung.

17 Dissemination Policy

The report generated from the data obtained will be part of the MSc research students' final dissertation. All relevant data used in the final PERFORM report will also be available to the general public via the consortium's website http://www.perform2020.eu.

18 Authorship Eligibility Guidelines

The results of the study will be submitted to a peer-reviewed medical journal. The International Committee of Medical Journal's (ICMJE) authorship criteria will be used to grant authorship:

- Substantial contributions to the conception or design of the work; or the acquisition, analysis, or interpretation of data for the work; AND
- Drafting the work or revising it critically for important intellectual content; AND
- Final approval of the version to be published; AND
- Agreement to be accountable for all aspects of the work in ensuring that questions related to the accuracy or integrity of any part of the work are appropriately investigated and resolved.

For peer review only - http://bmjopen.bmj.com/site/about/guidelines.xhtml

Protocol version number: 6.0
Protocol version date: 24/05/18
IRAS number: 248723

19 References

Bazeley, P. (2013) *Qualitative data analysis : Practical strategies*.

Van den Bruel, A., Haj-Hassan, T., Thompson, M.J., Buntinx, F., et al. (2010) Diagnostic value of clinical features at presentation to identify serious infection in children in developed countries: a systematic review. *The Lancet*. [Online] 375 (9717), 834–845. Available from: doi:10.1016/S0140-6736(09)62000-6.

Van Den Bruel, A., Jones, C.H.D., Thompson, M.J. & Mant, D. (2016) C-reactive protein point-of-care testing in acutely ill children: A mixed methods study in primary care. *Archives of Disease in Childhood*. [Online] Available from: doi:10.1136/archdischild-2015-309228.

Van Den Bruel, A., Thompson, M.J., Haj-Hassan, T., Stevens, R., et al. (2011) Diagnostic value of laboratory tests in identifying serious infections in febrile children: systematic review. *BMJ*. [Online] Available from: doi:10.1136/bmj.d3082.

Burchett, H.E.D., Leurent, B., Baiden, F., Baltzell, K., et al. (2017) Improving prescribing practices with rapid diagnostic tests (RDTs): Synthesis of 10 studies to explore reasons for variation in malaria RDT uptake and adherence. *BMJ Open*. [Online] 7 (3). Available from: doi:10.1136/bmjopen-2016-012973.

Bustinduy, A.L., Jeyaratnam, D., Douthwaite, S., Tonkin-Crine, S., et al. (2017) Point-of-care tests for infectious diseases: barriers to implementation across three London teaching hospitals. *Acta Paediatrica, International Journal of Paediatrics*. [Online] Available from: doi:10.1111/apa.13867.

Craig, J.C., Williams, G.J., Jones, M., Codarini, M., et al. (2010) The accuracy of clinical symptoms and signs for the diagnosis of serious bacterial infection in young febrile children: prospective cohort study of 15 781 febrile illnesses. *BMJ*. [Online] Available from: doi:10.1136/bmj.c1594.

Flick, U. (n.d.) *An introduction to qualitative research*.

Hardy, V., Thompson, M.J., Keppel, G.A., Alto, W., et al. (2017) Qualitative study of PC clinicians on POCT CRP for ARTI in US Adults. *BMJ Open*. [Online] Available from: doi:10.1136/bmjopen-2016- 012503 ▸.

Jones, C.H.D., Howick, J., Roberts, N.W., Price, C.P., et al. (2013) Primary care clinicians' attitudes towards point-of-care blood testing: A systematic review of qualitative studies. *BMC Family Practice*. [Online] Available from: doi:10.1186/1471-2296-14-117.

Lucas, P.J., Cabral, C., Hay, A.D. & Horwood, J. (2015) A systematic review of parent and clinician views and perceptions that influence prescribing decisions in relation to acute childhood infections in primary care. *Scandinavian Journal of Primary Health Care*. [Online] 33 (1), 11–20. Available from: doi:10.3109/02813432.2015.1001942.

Massin, M.M., Montesanti, J., Gérard, P. & Lepage, P. (2006) SPECTRUM AND FREQUENCY OF ILLNESS PRESENTING TO A PEDIATRIC EMERGENCY DEPARTMENT. *Acta Clinica Belgica*. [Online] 61 (4), 161–165. Available from: doi:10.1179/acb.2006.027 [Accessed: 10 May 2018].

Ritchie, J., Lewis, J., McNaughton Nicholls, C. & Ormston, R. (n.d.) *Qualitative research practice : a guide for social science students and researchers*.

Schot, M.J., Broekhuizen, B.D., Cals, J.W.L., Brussee, E., et al. (2017) C-reactive protein point-of-care testing in children with cough: qualitative study of GPs' perceptions How this fits in. *BJGP Open*. [Online] Available from: doi:10.3399/bjgpopen17X101193.

Stewart, E.H., Davis, B., Clemans-Taylor, B.L., Littenberg, B., et al. (2014) Rapid Antigen Group A Streptococcus Test to Diagnose Pharyngitis: A Systematic Review and Meta-Analysis. *PLoS ONE*. [Online] Available from: doi:10.1371/journal.pone.0111727.

World Health Organization (WHO) (2015) *Global Action Plan On Antimicrobial Resistance*. [Online]. Available from: http://www.wpro.who.int/entity/drug_resistance/resources/global_action_plan_eng.pdf.

For peer review only - http://bmjopen.bmj.com/site/about/guidelines.xhtml

Protocol version number: 6.0
Protocol version date: 24/05/18
IRAS number: 248723

20 Appendix

Appendix 1: Sampling Matrix

Participant type	London	Newcastle	**Total**
Paediatric Site Practitioner Nurses	2 Nurses	2 Nurses	4
Speciality Trainees	3 ED trainees<br>3 Paediatrics trainees	3 ED trainees<br>3 Paediatrics trainees	12
Consultants	4 ED Physicians<br>4 Paediatricians	4 ED Physicians<br>4 Paediatricians	16
**Total**	16	16	32

For peer review only

Protocol version number: 6.0
Protocol version date: 24/05/18
IRAS number: 248723

For peer review only

For peer review only - http://bmjopen.bmj.com/site/about/guidelines.xhtml

SRQR REPORTING STATEMENT FOR ORIGINAL QUALITATIVE RESEARCH

Title and abstract

**Title** - Concise description of the nature and topic of the study Identifying the study as qualitative or indicating the approach (e.g., ethnography, grounded theory) or data collection methods (e.g., interview, focus group) is recommended	Pg 1/Lines 1-2
**Abstract** - Summary of key elements of the study using the abstract format of the intended publication; typically includes background, purpose, methods, results, and conclusions	Pg 2-3/Lines 21-47

Introduction

**Problem formulation** - Description and significance of the problem/phenomenon studied; review of relevant theory and empirical work; problem statement	Pg 6-7/Lines 91-115
**Purpose or research question** - Purpose of the study and specific objectives or questions	Pg 7/Lines 113-115

Methods

**Qualitative approach and research paradigm** - Qualitative approach (e.g., ethnography, grounded theory, case study, phenomenology, narrative research) and guiding theory if appropriate; identifying the research paradigm (e.g., postpositivist, constructivist/ interpretivist) is also recommended; rationale**	Pg 7/Lines 118-123
**Researcher characteristics and reflexivity** - Researchers' characteristics that may influence the research, including personal attributes, qualifications/experience, relationship with participants, assumptions, and/or presuppositions; potential or actual interaction between researchers' characteristics and the research questions, approach, methods, results, and/or transferability	Pg 4/Lines 71-73, Pg 7/Lines 124-125
**Context** - Setting/site and salient contextual factors; rationale**	Pg 7/Lines 132-134
**Sampling strategy** - How and why research participants, documents, or events were selected; criteria for deciding when no further sampling was necessary (e.g., sampling saturation); rationale**	Pg 7-8/Lines 134-141
**Ethical issues pertaining to human subjects** - Documentation of approval by an appropriate ethics review board and participant consent, or explanation for lack thereof; other confidentiality and data security issues	Pg 8/Lines 153-154
**Data collection methods** - Types of data collected; details of data collection procedures including (as appropriate) start and stop dates of data collection and analysis, iterative process, triangulation of sources/methods, and modification of procedures in response to evolving study findings; rationale**	Pg 7-8/Lines 132-142

For peer review only

**Data collection instruments and technologies** - Description of instruments (e.g., interview guides, questionnaires) and devices (e.g., audio recorders) used for data collection; if/how the instrument(s) changed over the course of the study	Pg 8/Lines 141-144
**Units of study** - Number and relevant characteristics of participants, documents, or events included in the study; level of participation (could be reported in results)	Pg 19/Table 1
**Data processing** - Methods for processing data prior to and during analysis, including transcription, data entry, data management and security, verification of data integrity, data coding, and anonymization/de-identification of excerpts	Pg 8/Lines 143-152
**Data analysis** - Process by which inferences, themes, etc., were identified and developed, including the researchers involved in data analysis; usually references a specific paradigm or approach; rationale**	Pg 8/Lines 143-152
**Techniques to enhance trustworthiness** - Techniques to enhance trustworthiness and credibility of data analysis (e.g., member checking, audit trail, triangulation); rationale**	Pg 8/Lines 146-152

Results/findings

**Synthesis and interpretation** - Main findings (e.g., interpretations, inferences, and themes); might include development of a theory or model, or integration with prior research or theory	Pg 8-12/Lines 156-228
**Links to empirical data** - Evidence (e.g., quotes, field notes, text excerpts, photographs) to substantiate analytic findings	Pg 19-24/Box 1

Discussion

**Integration with prior work, implications, transferability, and contribution(s) to the field -** Short summary of main findings; explanation of how findings and conclusions connect to, support, elaborate on, or challenge conclusions of earlier scholarship; discussion of scope of application/generalizability; identification of unique contribution(s) to scholarship in a discipline or field	Pg 12-14/Lines 230-278
**Limitations** - Trustworthiness and limitations of findings	Pg 14/Lines 280-292

Other

**Conflicts of interest** - Potential sources of influence or perceived influence on study conduct and conclusions; how these were managed	Pg 4/Lines 70-73
**Funding** - Sources of funding and other support; role of funders in data collection, interpretation, and reporting	Pg 3-4/Lines 63-68

For peer review only

Supplement 7: POCTs availability at the study hospitals

**Hospital**	**Test**	**Vendor**
St. Mary's Hospital, London	Blood gas analyser	GEM 4000
	Blood glucose/ketones	Freestyle Precision Pro
	Urinalysis	Siemens Multistix 8 SG
	CRP*	Alere Afinion AS100
Great North Children's Hospital, Newcastle	Urine dipstick	Combur7 test
	Urine ketones	Ketodiastix Ascensia
	Urine microscopy	
	Blood glucose	Accucheck inform II
	Blood gas analyser	GEM premier 5000
	Influenza & RSV	Cepheid GeneXpert

*CRP POCTs were not available at St. Mary's Hospital at the time of study, however they previously had been available as part of a pilot

For peer review only

Supplement 8: Measures in place to ensure safe and efficient use of POCTs in the two hospitals

All point of care testing is overseen by a POCT committee that supervises point of care testing across the NHS trusts to which the two hospitals included in the study belong: Newcastle upon Tyne Hospitals NHS Foundation Trust with regards Great North Children Hospital in Newcastle, and Imperial College Healthcare NHS trust with regard St Mary's Hospital in London.

Within the two trusts, point of care testing is subject to strict governance and must be performed to the same quality standards as all testing undertaken within the United Kingdom Accreditation Service (UKAS) accredited laboratory departments, and to the same standards of the MHRA (Medicines and Healthcare products Regulatory Agency), the NHS Litigation Authority (NHSLA), the International Organisation for Standardisation (ISO), the Royal College of Pathologists (RCPath), and the Clinical Pathology Accreditation (CPA) standards.

Chaired by the POCT Manager, the POCT committee:

- Leads and coordinates POCT activities for the Trust in conjunction with the Trust's key objectives
- Maintains training records of all POCT operators throughout the Trust and coordinates staff education and facilitates training for all POCT devices. All POCT operators must be accredited by the POCT committee by undertaking POCT recognised training prior to patient testing. Competence in using POCT devices is achieved via Initial Training – this constitutes face-to-face training by either attending a timetabled training session or with a recognised 'Cascade Trainer'. Refresher Training is required every subsequent two years to maintain that competency
- Coordinates the provision of External Quality Assurance (EQA) schemes where possible, review EQA performance and offer advice on the improvement of poor performance
- Provides advice and help with documentation to Managers of areas on policies, operating procedures, training and competencies
- Undertakes Risk assessments for POCT devices where appropriate
- Leads and coordinates the validation of new POCT devices before implementation
- Provides evaluation of the performance of POCT equipment
- Coordinates audits and inspections to provide Trust assurance of quality patient care and value for money

For peer review only

BMJ Open

The role of Point-of-Care-Tests in the management of febrile children: A qualitative study of hospital-based doctors and nurses in England

Journal:	*BMJ Open*
Manuscript ID	bmjopen-2020-044510.R1
Article Type:	Original research
Date Submitted by the Author:	15-Feb-2021
Complete List of Authors:	Dewez, Juan ; London School of Hygiene and Tropical Medicine, Clinical Research Department<br>Li, Edmond; Imperial College London Faculty of Medicine, Surgery & Cancer; London School of Hygiene and Tropical Medicine Faculty of Infectious and Tropical Diseases, Clinical Research Department<br>Luu, Queena; London School of Hygiene and Tropical Medicine Faculty of Infectious and Tropical Diseases, Clinical Research Department<br>Emonts, Marieke; Great North Children's Hospital, Paediatric Immunology, Infectious Diseases & Allergy<br>Maconochie, Ian; Imperial College Healthcare NHS Trust, St Mary's Hospital, Paediatric Emergency Department<br>Nijman, R; Imperial College Healthcare NHS Trust, Paediatric Emergency Medicine; Imperial College London Faculty of Medicine, Department of Infectious Disease<br>Yeung, Shunmay; London School of Hygiene and Tropical Medicine Faculty of Infectious and Tropical Diseases, Clinical Research Department; Imperial College Healthcare NHS Trust, St Mary's Hospital, Department of Paediatrics
**Primary Subject Heading**:	Paediatrics
Secondary Subject Heading:	Emergency medicine, Infectious diseases, Diagnostics
Keywords:	QUALITATIVE RESEARCH, INFECTIOUS DISEASES, Paediatric A&E and ambulatory care < PAEDIATRICS, PAEDIATRICS, Paediatric infectious disease & immunisation < PAEDIATRICS, Diagnostic microbiology < INFECTIOUS DISEASES

SCHOLARONE™
Manuscripts

I, the Submitting Author has the right to grant and does grant on behalf of all authors of the Work (as defined in the below author licence), an exclusive licence and/or a non-exclusive licence for contributions from authors who are: i) UK Crown employees; ii) where BMJ has agreed a CC-BY licence shall apply, and/or iii) in accordance with the terms applicable for US Federal Government officers or employees acting as part of their official duties; on a worldwide, perpetual, irrevocable, royalty-free basis to BMJ Publishing Group Ltd ("BMJ") its licensees and where the relevant Journal is co-owned by BMJ to the co-owners of the Journal, to publish the Work in this journal and any other BMJ products and to exploit all rights, as set out in our licence.

The Submitting Author accepts and understands that any supply made under these terms is made by BMJ to the Submitting Author unless you are acting as an employee on behalf of your employer or a postgraduate student of an affiliated institution which is paying any applicable article publishing charge ("APC") for Open Access articles. Where the Submitting Author wishes to make the Work available on an Open Access basis (and intends to pay the relevant APC), the terms of reuse of such Open Access shall be governed by a Creative Commons licence – details of these licences and which Creative Commons licence will apply to this Work are set out in our licence referred to above.

Other than as permitted in any relevant BMJ Author's Self Archiving Policies, I confirm this Work has not been accepted for publication elsewhere, is not being considered for publication elsewhere and does not duplicate material already published. I confirm all authors consent to publication of this Work and authorise the granting of this licence.

For peer review only

The role of Point-of-Care-Tests in the management of febrile children: A qualitative study of hospital-based doctors and nurses in England

AUTHORS

Juan E. Dewez,[1*] Edmond C. Li,[1,2*] Queena F. Luu,[1*] Marieke Emonts,[3,4] Ian Maconochie,[5] Ruud Nijman,[5] Shunmay Yeung[1,6]

[*]Contributed equally

AFFILIATION

1: Clinical Research Department, Faculty of Infectious and Tropical Diseases, London School of Hygiene & Tropical Medicine, London, United Kingdom

2: Patient Safety Translational Research Centre, Department of Surgery and Cancer, Faculty of Medicine, Imperial College London, London, United Kingdom

3: Great North Children's Hospital, Paediatric Immunology, Infectious Diseases & Allergy, Newcastle upon Tyne Hospitals NHS Foundation Trust, Newcastle upon Tyne, United Kingdom

4: Translational and Clinical Research Institute, Newcastle University, Newcastle upon Tyne, United Kingdom

5: Section of Paediatric Infectious Diseases, Faculty of Medicine, Imperial College London, London, United Kingdom

6: Department of Paediatric Infectious Disease, St Mary's Imperial College Hospital London, United Kingdom

For peer review only

For peer review only - http://bmjopen.bmj.com/site/about/guidelines.xhtml

CORRESPONDING AUTHOR

Shunmay Yeung, Keppel Street, London WC1E 7HT, United Kingdom

Email: shunmay.yeung@lshtm.ac.uk

Phone: +44 2079272657

ABSTRACT

Objectives

The use of rapid point-of-care tests (POCTs) has been advocated for improving patient management and outcomes and for optimising antibiotic prescribing. However, few studies have explored healthcare workers' views about their use in febrile children. The aim of this study was to explore the perceptions of hospital-based doctors and nurses regarding the use of POCTs in England.

Study design

Qualitative in-depth interviews with purposively selected hospital doctors and nurses. Data were analysed thematically.

Setting

Two university teaching hospitals in London and Newcastle.

Participants

24 participants (paediatricians, emergency-department doctors, trainee paediatricians, and nurses).

Results

There were diverse views about the use of POCTs in febrile children. The reported advantages included their ease of use and the rapid availability of results. They were seen to contribute to faster clinical decision-making; the targeting of antibiotic use; improvements in patient care, flow and monitoring; cohorting (ie the physical clustering of hospitalised patients with the same infection to limit spread); and enhancing communication with parents. These advantages were less evident when the turnaround for

For peer review only

For peer review only - http://bmjopen.bmj.com/site/about/guidelines.xhtml

results of laboratory tests was 1-2 hours. Factors such as clinical experience and specialty, as well as the availability of guidelines recommending POCT use, were also perceived as influential However, in addition to their perceived inaccuracy, participants were concerned about POCTs not resolving diagnostic uncertainty or altering clinical management, leading to a commonly expressed preference for relying on clinical skills rather than test results solely.

Conclusion

In this study conducted in two university teaching hospitals in England, participants expressed mixed opinions about the utility of current POCTs in the management of febrile children. Understanding the current clinical decision-making process, and the specific needs and preferences of clinicians in different settings will be critical in ensuring the optimal design and deployment of current and future tests.

Strengths & limitations of this study

Strengths

- A specific focus on the paediatric population.
- The inclusion of two hospitals in two different settings.
- A multi-disciplinary and diverse group of participants including nurses and doctors, with different levels of clinical experience and training.

Limitations

- The study took place in university teaching hospitals and did not explore the views of clinicians in other settings.
- The study explored the views of participants on several POCTs used in febrile children, limiting the amount of detail obtained about specific POCTs.

FUNDING DECLARATION

For peer review only - http://bmjopen.bmj.com/site/about/guidelines.xhtml

JED, ME, and SY are supported by PERFORM, a consortium funded by the European Union's Horizon 2020 programme, under grant agreement No. 668303. RGN was supported by NIHR Academic clinical fellowship (CF- 2015-21-016) and lectureship (CL-2018-21-007) award programme. The funding bodies did not take part in the design of the study and data collection, and did not take part in the data analysis and interpretation of results. The funding bodies did not take part in the writing of the manuscript.

COMPETING INTERESTS

None declared

DATA SHARING

The datasets generated and/or analysed during the current study will be made available upon reasonable request through the London School of Hygiene and Tropical Medicine data depository (LSHTM Data COMPASS).

WORD COUNT: 3407

For peer review only

INTRODUCTION

Febrile illness is one of the most common reasons for children to present to hospital, and managing it can often be challenging.[1,2] The majority of febrile children will have self-limiting infections which can be safely managed at home, often without antibiotics.[3-5] However, a few will have potentially severe infections and identifying these children from the others can be difficult, as clinical symptoms and signs are often non-specific in children.[3] Consequently, they may be prescribed antibiotics that are not needed, subjected to invasive tests, and admitted for monitoring whilst awaiting microbiology results.[4,5] As well as the direct impact this may have on children and parents in terms of distress, costs, and inconvenience,[6] there may also be indirect impacts in terms of antimicrobial resistance (AMR).[7]

The World Health Organization advocates the use of rapid point-of-care tests (POCTs) to reduce the use of antibiotics.[8] POCTs have the potential to aid clinical decision-making, limit the use of other invasive tests, and improve the use of medical resources in general.[9] However, achieving these impacts depends on several factors, including whether POCTs are taken up by healthcare workers and how they influence clinical decisions.[10] This cannot be assumed. For example, when malaria POCTs were introduced, many clinicians continued to prescribe antimalarials despite patients testing negative.[11]

The types of POCTs that are currently available for the management of childhood infections include urine dipsticks; rapid tests for Group A Streptococcal throat infections ("rapid strep test"); rapid respiratory virus tests for Respiratory Syncytial Virus (RSV) or Influenza; and blood-based tests including C-reactive protein (CRP) and blood gas analysers.

To date research exploring the views of clinicians on the use of POCTs for managing patients with infections have predominantly focused on general practitioners (GPs) treating adult patients.[12-19] To the best of our knowledge, only one study pertains to the perceptions of hospital-based healthcare workers regarding the use of POCTs in children.[20]

For peer review only - http://bmjopen.bmj.com/site/about/guidelines.xhtml

104 The aim of this study was to explore the experiences and attitudes of hospital-based doctors and nurses regarding the use of POCTs in children with acute infections, and to identify factors that influence their use.

METHODS

Qualitative in-depth interviews with doctors and nurses were conducted to better understand their experiences and attitudes regarding POCT use in children with suspected infection.[21] Ontologically, our approach adhered to subtle realism, and epistemologically to interpretivism,[22,23] because we believed that although reality (i.e., the use of diagnostics) exists independently of our beliefs, the interpretation of participants' perceptions and how they make sense of their experiences were needed to ascertain what influences POCT usage.

The research team comprised of two public health professionals and five paediatricians with experience in qualitative methods. Two topic guides, one for doctors and one for nurses, were developed based on a review of the literature (Supplement 1). These were piloted with two paediatricians and two nurses at St Mary's Hospital in London. The pilot interviews were not included in the analysis. The topic guides were iteratively adapted during data collection after regular debriefings between EL, QL, and JED to discuss main findings, refine questions and explore new areas of inquiry.[24]

Eligible participants comprised of doctors and nurses with responsibility for making decisions about whether or not to use POCTs on febrile children in two university teaching hospitals: the Great North Children's Hospital in Newcastle, and St Mary's Hospital in London. We purposively chose not to interview other sub-specialty paediatricians such as paediatric infectious disease specialists as they are less often responsible for decisions on POCT use in the acute hospital setting. Purposive maximum variation sampling was used, focusing on participants with different clinical roles and varying years of experience. A sampling matrix was developed specifying the targeted number of participants of each

For peer review only - http://bmjopen.bmj.com/site/about/guidelines.xhtml

healthcare worker category (consultant paediatrician, paediatric trainee with three or more years training, paediatric trainees with less than three years training, and ED nurses) (Supplement 2). As members of staff of the two hospitals, SY, RN, IM, ME contacted available paediatric staff who fit the inclusion criteria to ascertain their interest in being interviewed for the study. Those who agreed, were then followed-up by email by the interviewers (EL and QL) who provided a detailed participant information sheet, obtained written informed consent, and arranged the interview date. The two interviewers did not know participants beforehand. The study aimed to include 22-30 participants, a sample size which is in line with other qualitative studies investigating healthcare workers' perceptions, and was expected to allow for data saturation.[12,13,18,25] Identifying the actual point of data saturation was important to make best use of the available time and resources and to minimise the disruption caused by diverting healthcare providers from their normal clinical activities. During the regular debriefings, EL, QL, and JED identified whether any new ideas had been raised by the participants. Once no new ideas had emerged from the last several interviews, the authors agreed that data saturation has been reached. One-on-one, audio-recorded interviews took place during normal work hours at the respondents' workplace between June and August 2018.

The interview records were transcribed verbatim by EL and QL and back-checked against the recording for accuracy. The transcripts were annonymised (only participants' job titles were documented) and then analysed thematically.[24] The analysis was both deductive (i.e., partly based on pre-existing knowledge) and inductive (i.e., additional concepts were identified from the dataset). EL and QL generated two initial lists of coding from the dataset through line-by-line coding (Supplement 3). EL, QL, and JED then agreed on an analytical framework comprising the most relevant codes and used the framework to re-code the entire dataset. Matrices were used to display data related to initial themes (Supplement 4). Iterative exploration of the data identified patterns within the dataset and continued until no new themes were identified. Further refinement was carried out with the rest of the research

For peer review only - http://bmjopen.bmj.com/site/about/guidelines.xhtml

team to ensure themes were internally coherent and distinctive to produce clear descriptions and explanations of each theme.

Ethical approval was obtained from the London School of Hygiene & Tropical Medicine Ethics Committee (Ref: 15040-15088). Health Research Authority approval (Ref: IRAS 248723) and administrative approval were obtained from the Imperial College Healthcare NHS Trusts (Ref: 18SM4662) and Newcastle Upon Tyne Hospital NHS Foundation (Ref: 248723).

Patient and public involvement

Patients and members of the public were not involved in the design of this study.

RESULTS

A total of 24 out of 27 invited healthcare workers were interviewed (Table 1); two nurses and one trainee doctor did not reply to the invitation. Interviews lasted for 60-75 minutes. Most participants fully engaged with the interviews and readily shared their views on the topics of interest with little direction from the interviewers. Participants' experience of using POCTs varied at the two hospitals. Although all participants reported routine use of urinary dipsticks, Respiratory Syncytial Virus (RSV) rapid tests, and blood gas tests, some participants from St Mary's Hospital recalled also having access to CRP and rapid strep tests in past studies. (Please see Supplement 5 and 6 for description of POCT availability and oversight at the two hospitals).

A range of reasons was given for and against using POCT. These have been grouped into eight themes (Table 2): five pertaining to factors encouraging the use of POCTs (Box 1) and three for factors discouraging the use of these tests (Box 2) in the management of febrile children.

Factors encouraging the use of rapid POCTs

For peer review only - http://bmjopen.bmj.com/site/about/guidelines.xhtml

Most participants were keen on using POCTs due to their inherent advantages (Box 1). One of the main cited advantages was their ease of use and speed with which they can provide results when compared to hospital laboratory-based diagnostics. This was seen to contribute to accelerating decision-making and improving patient flow in busy wards. However, a few participants questioned the magnitude of this impact and noted that these advantages were largely context dependent. In circumstances where laboratories were able to return results within one to two hours, some felt that the potential benefit in terms of the rapidity of POCTs result was outweighed by the perceived greater accuracy of laboratory-based tests, the possibility to monitor the patient's clinical evolution, and the flexibility to see other patients in parallel whilst awaiting laboratory results.

Urine dipsticks and CRP POCTs were reported to support quicker decision-making on whether or not to prescribe antibiotics or to perform additional diagnostic tests. Rapid RSV testing was valued for assisting with the timely cohorting of children with respiratory infections, thereby limiting the potential for nosocomial transmission. The ability to use rapid POCTs without the help of other doctors or nurses, and to get results independently from the laboratory, was important for many participants, because it allowed for easier repeat testing for monitoring purposes and provided participants with a greater sense of direct control over the care of their patients. Blood gas POCTs were a notable example of this. Additionally, obtaining results rapidly allowed clinicians to quickly communicate results to parents which helped them to justify clinical decisions and to reduce overall parental anxieties, which was felt to have contributed to improved caregivers' experience. Finally, a noted benefit of blood-based POCTs was that they could be performed on finger-prick blood rather than requiring venepuncture which was seen as more invasive and difficult to perform. This contributed to their acceptability not only to clinicians but also to children and their parents.Aside from the described advantages inherent to POCTs, there were also other factors which influenced the use of POCTs. For doctors, their level of experience appeared to influence their use of diagnostics. There was a prevailing perception that less experienced clinicians used

For peer review only - http://bmjopen.bmj.com/site/about/guidelines.xhtml

more POCTs as the tests served as learning tools, as well as a “safety net” if their clinical acumen proved inadequate. Moreover, participants also alluded that diagnostics use, including POCTs, varied according to their perceptions of the risk for severe infections, which itself was dependent on the clinician’s specialty training and the population seeking care. Some participants believed that general practitioners (GPs) used less diagnostics than emergency department paediatricians, who in turn use less diagnostics than paediatric infectious diseases specialists. This was thought to be because the patient population seen by the three types of clinicians differed, with GPs seeing proportionally fewer children with severe infections.

Finally, clinical guidelines were also seen as important. Participants reported that guidelines including recommendations on the use of the tests and the interpretation of results in children would be highly influential in their decision to use POCTs. This was because guidelines were perceived as both providing reassurance of best practices and protection against malpractice**.**

Factors discouraging the use of rapid POCTs

Several participants were concerned about the accuracy of the test results (Box 2). It was commonly perceived that there were discrepancies in relation to the patient’s clinical presentation and/or when compared to laboratory tests. These concerns undermined clinicians’ trust in POCTs, and often resulted in subsequent confirmatory laboratory tests to be performed prior to making a clinical decision. Many also cited the lack of strong evidence supporting the accuracy of some POCTs for paediatric use, some noting that existing POCTs such as CRP had predominantly only been validated in adults.

Another notable reason for hesitancy about some POCTs was their perceived inability to adequately address diagnostic uncertainty. Some participants mentioned that with current POCTs that provide numerical results (e.g. CRP levels) instead of binary results (“positive/negative”), the tests were unable to provide a definitive clinical answer when results were within an “intermediate” range. Other

For peer review only - http://bmjopen.bmj.com/site/about/guidelines.xhtml

participants felt that current POCTs were not able to reliably predict which initially well-appearing febrile children were at risk of clinical deterioration and therefore required hospitalisation. This was perceived to be an important feature of diagnostics for managing fever in children. It was also commonly noted that some POCTs were only designed to detect a single pathogen such as RSV, which was of limited utility compared to the range of different pathogen that were detectable from laboratory-based panels. Altogether, this failure to resolve diagnostic uncertainty was seen to limit the potential for POCTs to alter clinical decisions. In addition, even when POCTs were perceived as accurate, it was felt their results might not prove consequential enough to warrant a change in the patient's overall clinical management. An example of this was the rapid Group A strep test. While the test was acknowledged to be sensitive and specific, some participants felt that they would make a decision to prescribe antibiotics irrespective of whether a test confirmed the presence Group A streptococcus or not. However, the results of other POCTs such as urine dipsticks, greatly influenced clinical management for many participants.

Overall, in view of the perceived limitations of POCTs, participants expressed a preference for prioritising their clinical skills (history, examination, observation of patients) and acumen over the reliance on diagnostics in guiding their clinical management of febrile children.

Comparison of views between types of participants

The views expressed by different types of participants were broadly similar. However, there was a trend towards some differences emerging, mainly between trainees versus consultants and nurses. Specifically consultants stressed the importance of relying on their clinical acumen, more so than trainees. They also felt that trainees used more POCTs, a sentiment was also shared by nurses, but this was seldom reported by trainees. Trainees more often mentioned how CRP results can appear normal for a child at the beginning of a serious infection, whilst consultants seemed more concerned with the difficulty of

For peer review only - http://bmjopen.bmj.com/site/about/guidelines.xhtml

interpreting intermediate CRP results. The potential contribution of POCT usage in improving antibiotic stewardship was mentioned widely but generally with stronger emphasis by consultants and nurses than trainees. Finally, nurses mentioned their willingness to perform more POCTs independently of doctors in the future. Whilst consultants and trainees rarely commented on who should be performing the tests, when they did, they were not against nurses using POCTs independently.

DISCUSSION

This study provides insight into what hospital-based doctors and nurses in England think of POCTs in relation to managing children with suspected infection, a topic on which little has previously been published. Within the two hospitals, we interviewed a diverse range of healthcare providers who have responsibility for deciding on the use of POCTs including both consultants in general paediatrics and emergency paediatrics, paediatric trainees with different levels of experience, and nurses. A range of reasons for and against the use of POCTs was identified.

The advantages of POCTs over laboratory-based tests in terms of their ease of use and the rapid availability of results were widely recognised. Other reported potential benefits included faster clinical decision-making; improved monitoring of patients; more targeted use of antibiotics; enhanced patient flow; the rapid cohorting of inpatients with respiratory infection; and improved communication with parents. However, these advantages were less evident when laboratories were able to return results within a few hours. Being able to perform blood-based POCTs on finger-prick samples was also seen as less invasive and therefore more acceptable to clinicians, parents, and children compared to tests requiring venepuncture. Guidelines were also perceived to be important in guiding patient management, including the use of POCTs.

However, participants also mentioned a number of important limitations of POCTs. These include their perceived inaccuracy, their inability to predict risks of clinical deterioration, and the limited scope of diagnostic information from certain tests. Some POCTs were not seen as being able to solve diagnostic

For peer review only - http://bmjopen.bmj.com/site/about/guidelines.xhtml

uncertainty and others were perceived as having a limited role in guiding or changing overall clinical management. Altogether, these perceived shortcomings reinforced the overarching view that clinicians should trust their clinical acumen more than diagnostics.

The only other study that we were aware of which specifically explored the views of paediatricians and nurses about rapid POCTs usage in children, found similar results. Participants valued their ease and speed of use, which enabled early treatment, cohorting, decreasing antibiotic prescription, and better communication with parents.[20] Some participants were also concerned about the accuracy of the tests and about the limited changes in clinical management enabled by POCTs.

Some studies exploring the views of GPs reported similar advantages and concerns, especially regarding the accuracy of tests.[12,14,20,25,26,27] However, there were conflicting results in terms of the perceived invasiveness of POCTs: two studies found that GPs would not use blood-based POCTs because they felt finger pricking was too invasive in children,[12,25] while another study reported that GPs, perceived the pain to be short-lived and recounted that children were actually interested by the use of rapid tests during consultations.[28] Other GPs felt it was easy to persuade parents that antibiotics were not needed compared to adult patients, limiting the need to use POCTs for this purpose.[25] Some GPs reportedly also feared that the use of POCTs could actually increase antibiotic prescription. [27,28] Finally, in contrast to our finding that for some paediatricians the perceived limitations of POCTs reinforced their preference of relying on their clinical acumen over diagnostics, some of the studies with GPs reported that there were concerns that the perceived advantages of POCTs could lead to the tests being over adopted and used, which could potentially undermine clinical acumen in the long term. [13,14,27]

Of note, national and local paediatric guidelines were seen to be important in guiding patient management, including the use of diagnostics. At the time of the study, the use of urine dipstick and blood gas analysis were mentioned in relevant guidelines for managing children with suspected

For peer review only - http://bmjopen.bmj.com/site/about/guidelines.xhtml

infections. These guidelines were widely available in hospital acute care settings in England. However, to date, neither CRP POCT nor rapid strep tests have been recommended for the management of acute childhood infections by the National Institute for Health and Care Excellence (NICE), and neither were available in the two hospitals at the time of this study.

The study had some limitations, and also highlighted a number of important areas for further research. Firstly, as the study aimed to explore the role of POCTs used in febrile children rather than focusing on one or two specific tests, it was sometimes not possible to decipher whether the participants' comments were referring to specific POCTs or POCTs in general. Given the substantial differences in the attributes and roles of different POCTs, deeper exploration about specific POCTs would be helpful.

Secondly, several members of our research team are active paediatricians with varying experience of POCTs. However, we have trained and worked in wide variety of countries and settings, and hold a wide range of views about POCTs ourselves. Moreover, two public health specialists with limited experience in diagnostics or paediatrics research conducted the interviews, contributed to the data analysis, interpretation, and drafting of results. Therefore, although as in any qualitative research, there is an inherent risk of subjectivity in the interpretation and presentation of the results, we believe that the breadth of views and backgrounds in the team have helped to minimise this risk.

Thirdly, the scope of this study was restricted to two teaching hospitals in England and did not capture the views of paediatricians in district general hospitals, nor general practitioners. Further qualitative research with other key stakeholders, such as clinicians in other sub-specialties and those based in district general hospitals or in the community, and with parents and children themselves, would be very informative. As the study was limited to England, we could not make any comparisons with other countries where the culture of clinical decision-making and availability of POCTs may differ. As the use of POCTs seem to vary greatly across countries, it would be very interesting to carry out similar studies elsewhere. It would also be useful to elucidate in which patients and where within the care pathway,

For peer review only - http://bmjopen.bmj.com/site/about/guidelines.xhtml

specific POCTs would have the most impact. Finally, this study was carried out in the pre-COVID-19 era. Since then, the diagnostics landscape has changed dramatically; it would informative to carry out a similar study now, as the views about POCT use may likely have evolved since.

Conclusion

In this study conducted in two university teaching hospitals in England, participants expressed mixed opinions about the utility of current POCTs in the management of febrile children. Whilst the many potential advantages of POCTs were well recognised, concerns about their accuracy and failure to solve diagnostic uncertainty and alter clinical management, limited the perceived utility of current POCTs in this setting. Understanding the clinical decision-making process and the concerns and needs of clinicians in different settings, will be critical in the development of new tests and in evaluating the potential impact on clinical management and other outcomes. Further studies in different countries and health care settings in the post-COVID-19 era, should help to inform this understanding.

AUTHOR CONTRIBUTIONS

Juan Emmanuel Dewez (JED), Edmond C. Li (EL), and Queena F. Luu (QL) contributed equally to this paper and have been listed in alphabetical order. Shunmay Yeung (SY) conceived of the study and had overall responsibility. All authors input into the design of the study and the study materials. Marieke Emonts (ME), Ian Maconochie (IM), Ruud G Nijman (RGN) and SY identified participants. EL and QL conducted and transcribed the interviews. EL, JED, and QL analysed the data and wrote the manuscript. EL, JED, and SY made substantial revisions. All authors provided input into drafts of the manuscript and agree on the contents of the final version.

For peer review only - http://bmjopen.bmj.com/site/about/guidelines.xhtml

REFERENCES

1. Sands R, Shanmugavadivel D, Stephenson T, Wood D. Medical problems presenting to paediatric emergency departments: 10 years on. Emergency medicine journal : EMJ. 2012 May;29(5):379-82.

2. Alpern ER, Stanley RM, Gorelick MH, Donaldson A, Knight S, Teach SJ, et al. Epidemiology of a pediatric emergency medicine research network: the PECARN Core Data Project. Pediatric emergency care. 2006 Oct;22(10):689-99.

3. Van den Bruel A, Haj-Hassan T, Thompson M, Buntinx F, Mant D. Diagnostic value of clinical features at presentation to identify serious infection in children in developed countries: a systematic review. Lancet (London, England). 2010;375(9717):834-45.

4. De S, Williams GJ, Hayen A, Macaskill P, McCaskill M, Isaacs D, et al. Accuracy of the "traffic light" clinical decision rule for serious bacterial infections in young children with fever: a retrospective cohort study. BMJ (Clinical research ed). 2013;346:f866.

5. Nijman RG, Vergouwe Y, Thompson M, van Veen M, van Meurs AHJ, van der Lei J, et al. Clinical prediction model to aid emergency doctors managing febrile children at risk of serious bacterial infections: diagnostic study. BMJ : British Medical Journal. 2013;346.

6. Leigh S, Grant A, Murray N, Faragher B, Desai H, Dolan S, et al. The cost of diagnostic uncertainty: a prospective economic analysis of febrile children attending an NHS emergency department. BMC medicine. 2019;17(1):48.

7. European Centre for Disease Control, European Food Safety A, European Medicines A. ECDC/EFSA/EMA second joint report on the integrated analysis of the consumption of antimicrobial agents and occurrence of antimicrobial resistance in bacteria from humans and food-producing animals. EFSA Journal. 2017;15(7):e04872-n/a.

8. WHO. World Health Organization. Global action plan on antimicrobial resistance 2015. Available from http://www.who.int/antimicrobial-resistance/global-action-plan/en. Accessed 20/12/17.

9. Larsson A, Greig-Pylypczuk R, Huisman A. The state of point-of-care testing: a European perspective. Upsala journal of medical sciences. 2015;120(1):1-10.

10. Horvath AR, Lord SJ, StJohn A, Sandberg S, Cobbaert CM, Lorenz S, et al. From biomarkers to medical tests: the changing landscape of test evaluation. Clinica chimica acta; international journal of clinical chemistry. 2014;427:49-57.

11. Burchett HE, Leurent B, Baiden F, Baltzell K, Bjorkman A, Bruxvoort K, et al. Improving prescribing practices with rapid diagnostic tests (RDTs): synthesis of 10 studies to explore reasons for variation in malaria RDT uptake and adherence. BMJ Open. 2017;7(3):e012973.

12. Butler CC, Simpson S, Wood F. General practitioners' perceptions of introducing near-patient testing for common infections into routine primary care: a qualitative study. Scandinavian journal of primary health care. 2008;26(1):17-21.

For peer review only

For peer review only - http://bmjopen.bmj.com/site/about/guidelines.xhtml

13. Cals JW, Chappin FH, Hopstaken RM, van Leeuwen ME, Hood K, Butler CC, et al. C-reactive protein point-of-care testing for lower respiratory tract infections: a qualitative evaluation of experiences by GPs. Family practice. 2010;27(2):212-8.

14. Wood F, Brookes-Howell L, Hood K, Cooper L, Verheij T, Goossens H, et al. A multi-country qualitative study of clinicians' and patients' views on point of care tests for lower respiratory tract infection. Family practice. 2011;28(6):661-9.

15. Pulcini C, Pauvif L, Paraponaris A, Verger P, Ventelou B. Perceptions and attitudes of French general practitioners towards rapid antigen diagnostic tests in acute pharyngitis using a randomized case vignette study. Journal of Antimicrobial Chemotherapy. 2012;67(6):1540-6.

16. Leydon GM, McDermott L, Moore M, Williamson I, Hobbs FD, Lambton T, et al. A qualitative study of GP, NP and patient views about the use of rapid streptococcal antigen detection tests (RADTs) in primary care: 'swamped with sore throats?'. BMJ open. 2013;3(4).

17. Hardy V, Thompson M, Alto W, Keppel GA, Hornecker J, Linares A, et al. Exploring the barriers and facilitators to use of point of care tests in family medicine clinics in the United States. BMC family practice. 2016;17(1):149.

18. Hardy V, Thompson M, Keppel GA, Alto W, Dirac MA, Neher J, et al. Qualitative study of primary care clinicians' views on point-of-care testing for C-reactive protein for acute respiratory tract infections in family medicine. BMJ open. 2017;7(1):e012503.

19. Eley CV, Sharma A, Lecky DM, Lee H, McNulty CAM. Qualitative study to explore the views of general practice staff on the use of point-of-care C reactive protein testing for the management of lower respiratory tract infections in routine general practice in England. BMJ open. 2018;8(10):e023925.

20. Bustinduy AL, Jeyaratnam D, Douthwaite S, Tonkin-Crine S, Shaw R, Hyrapetian L, et al. Point-of-care tests for infectious diseases: barriers to implementation across three London teaching hospitals. Acta paediatrica (Oslo, Norway : 1992). 2017.

21. Flick U. An introduction to qualitative research. 5th ed. SAGE Publications; 2014.

22. Ritchie J, Lewis J, Nicholls CMN, Ormston R. Qualitative Research Practice: A Guide for Social Science Students and Researchers: SAGE Publications; 2013.

23. Durrand MAC, Tracy. (2014) Principles of social research . 2nd ed. Maidenhead: Open University Press.

24. Braun V, Clarke V. Using thematic analysis in psychology. Qualitative Research in Psychology. 2006;3(2):77-101.

25. Schot MJ, Broekhuizen BD, Cals JW, Brussee E, de Wit NJ, Verheij TJ, et al. C-reactive protein point-of-care testing in children with cough: qualitative study of GPs' perceptions. BJGP Open. 2018;1(4):bjgpopen17X101193.

For peer review only

26. Jones CH, Glogowska M, Locock L, Lasserson DS. Embedding new technologies in practice - a normalization process theory study of point of care testing. BMC health services research. 2016;16(1):591.

27. Turner PJ, Van den Bruel A, Jones CHD, Plüddemann A, Heneghan C, Thompson MJ, et al. Point-of-care testing in UK primary care: a survey to establish clinical needs. Family practice. 2016;33(4):388-94.

28. Van den Bruel A, Jones C, Thompson M, Mant D. C-reactive protein point-of-care testing in acutely ill children: a mixed methods study in primary care. Archives of disease in childhood. 2016 Apr;101(4):382-5.

For peer review only

For peer review only - http://bmjopen.bmj.com/site/about/guidelines.xhtml

TABLES

Table 1. Number of different types of health care workers interviewed

**Healthcare workers**	**Hospital Site**		**Total**
	SMH, London	GNCH, Newcastle	
Nurses – Paediatric Emergency Department	1	3	4
Consultants – Paediatric Emergency Department	3	2	5
Consultants – General Paediatrics	2	1	3
Trainee doctors – Senior House Officers	4	3	7
Trainee doctors – Registrars	2	3	5
**Total**	12	12	**24**

SMH: St Mary's Hospital London
GNCH: Great North Children's Hospital Newcastle

For peer review only

Table 2. Summary of themes

**FACTORS ENCOURAGING USE OF POCTS**<br>*Advantages*<br>• Ease of use and rapid availability of test results (leading to faster decision making and enhanced patient flow)<br>• Improved clinical care (resulting from targeted antibiotic treatment, facilitated patient cohorting, closer monitoring, and improved communication with caregivers)<br>• Minimal test invasiveness<br><br>*Other factors*<br>• Being less experienced<br>• Being a paediatric Infectious Disease specialist > general paediatrician> GP<br>• Guidelines recommending their use
**FACTORS DISCOURAGING USE OF POCTS**<br>• Concerns over test accuracy<br>• Failure to resolve diagnostic uncertainty and alter clinical management

For peer review only - http://bmjopen.bmj.com/site/about/guidelines.xhtml

- Preferential reliance on clinical skills and acumen

339

Box 1. Direct quotes about the factors that encourage the use of rapid POCTs

ADVANTAGES

Ease of use and rapid availability of test results

"If it's something the nurses could do really easily and quickly then it would help again, the flow and performance through the department. So let's say it takes 20 minutes to wee [make urinate] a child and it takes a specialist, a nurse, and the doctor...the nurse holding and etc, and you've got a really busy queue, and if you've got a really easy finger prick test that a nurse can do." [ED Paeds Consultant 1]

"Because it [POCTs in general] would just make things a lot quicker. Decision making a lot quicker. Being seen by doctors a lot quicker." [Nurse 3]

"So, you still might sit on the fence and want to admit and observe but it helps you just maybe make that decision a little bit quicker." [ED Paeds Consultant 1]

"So speed is probably the main advantage really and by virtue of being less people involved, it's a bit more accessible. You can also do them yourself, so you don't necessarily have to wait for other people to do the test." [Trainee 8]

Improved clinical management of febrile children

a) More targeted antibiotic treatment

"So that [urine dipstick] might change your choice of...not only whether you would give them antibiotics, but also what antibiotics you would give." [Trainee 9]

"I think that [POCTS in general] would help us with our overprescribing but also help reassure us that we are finding the ones that do need antibiotics and getting them to them [the children]." [ED Paeds Consultant 2]

"...I think bloods is probably one of the bigger ones...it just gives a much better indication as to whether or not we would treat with antibiotics or what...like...what kind of route you would go down..." [Nurse 3]

b) Deciding cohorting and disposition of patient

"The RSV test, we primarily..., are used for children who are being admitted. So we don't use it as a diagnostic test per se for children in the ED. But they use it for cohorting children if they are admitted to the ward." [ED Paeds Consultant 5]

"You know that kid where you are a little bit unsure, then the CRP come back a 5. And you were kinda thinking that it was probably ok, then it would influence a bit. Whereas the CRP

For peer review only - http://bmjopen.bmj.com/site/about/guidelines.xhtml

came back at 60 or 100, then you be like, right they definitely need to come in (be hospitalised) and that sort of stuff.” [ED Paeds Consultant 1]

“With the urine [dipstick], I think we're determining whether they need other tests really and whether we need to screen further or look for other sources of infection.” [ED Paeds Consultant 2]

c) Closer monitoring for clinical changes

“It's the repeat tests that I think that's the bigger advantage that they allow more easy repeat so you can assess the response to your treatment […] I think that is the single biggest advantage ‘cause it gives you the quantification of the effect of your treatment.” [ED Paeds Consultant 4]

“CRP (…), I think it is useful for monitoring if you've got a patient that is not following the usual course. Like if you got a drop in CRP and a raise again. I think it's very useful” [ED Paeds Consultant 2]

d) *Improved communication with caregivers*

“Parents find it quite stressful on how long like something bloods can take. They often come in and ask like how long it's going to be until my bloods are back. At least with the urine and the blood gas, you can kind of tell them fairly instantly. Like put their mind at rest and things.” [Nurse 1]

“I think parents put great faith in technology. If you have a CRP that is negative, then it's negative. And if it's positive, then it's positive. So I think it's much more black and white way. And so, I think...it can be very convincing for them. So I think it can be helpful in that way.” [Trainee 9]

“I think if somebody [parents] wanted antibiotics and I was sure they didn't [need] and the [rapid] CRP was there, I would be tempted, even though I thought know that it's not necessary evidence based to do a POCT, and say your CRP is only 6, you don't need antibiotics. [ED Paeds Consultant 5]

Minimal test invasiveness

“I might use that ‘cause it’s easy to do whereas I wouldn’t want to do a proper venous blood test on a child because it’s a painful procedure. A child would tolerate a little finger-prick, not like it, but they will tolerate it. Whereas a proper blood test involves lots of screaming, time, big needle, waiting around for hours.” [Gen Paeds Consultant 2]

“Just thinking about, managing a sweaty toddler whose heart can be tachycardic who I think is probably going to be ok, and I'm struggling to get bloods, but if I could do a finger prick and I can get results in ten minutes, if it was a CRP of 100, I could take my time that we've got access and reassure the parents of why that was whereas if it was ten, I'd say well alright, we don't have to do bloods, we're just going to keep you here for a few hours and see how you

For peer review only - http://bmjopen.bmj.com/site/about/guidelines.xhtml

For peer review only

are...so I think that would be helpful." [ED Paeds Consultant1]

OTHER FACTORS

Training, experience, and specialty

"When you are a junior doctor you do a bit of more investigations and then you build a bit more of a profile and you get a feel for whether children are more unwell or not. [As a senior,] you probably tend to use less investigations." [ED Paeds Consultant 1]

"We would have a tendency in the A&E to do more investigations than a GP would...it's all about what you are used...what population that you see. So obviously, if you are an ID [infectious diseases] consultant, then you're seeing all the weird and wonderful so all the other stuff is filtered out by parents and GP and then by A&E and so the population that they see is different." [ED Paeds Consultant 1]

"If I had a point of care available for other things, I would use it. I would use a CRP, an FBC, I mean I would use everything if I can get it instantly from a finger-prick size sample. I know there is a feeling that probably when you get more senior, that you would sometimes you don't want the information from a blood test but at my level, I would..." [Trainee_5]

Guidelines

"And so, I think we are very influenced by guidelines because we feel securities with guidelines. And so, to do a POCT, I would do it [if it is in the guidelines] because if I don't do it and it goes wrong, how will I defend myself. And essentially in fever, I already feel vulnerable." [Trainee 9]

"So I think with a POCT around fever, then you have to have a guideline about how you are gonna use the results. Because especially things like CRP, everyone is going to interpret CRP differently. (…) And so it would have to be a part of a guideline on how to interpret the results." [Trainee 9]

"If the guideline says to do...if there was a well-respected guideline in place for a particularly clinical scenario that says you should use this test, then of course, I would expect we would have to have a very good reason of why we aren't doing that test." [ED Paeds Consultant 4]

For peer review only - http://bmjopen.bmj.com/site/about/guidelines.xhtml

Box 2. Factors discouraging the use of rapid POCT

Concerns over test accuracy

"It may still be a viral infection and you have a borderline CRP and it may mean you have to go on and do further tests." [ED Paeds Consultant 2]

"There is a perception that they [POCTs] are less reliable, so often, there's a follow up with the formal lab equivalent…if you are shown a strong evidence base for accuracy of each POCT test, that would be critical for adoption." [Trainee 11]

"… if there was one test that could tell me if it wasn't bacterial infection, then yup. Fantastic. Something better than CRP. Yeah, it'd definitely be useful for that. If there was a viral diagnostic test that could tell me every single virus. Absolutely. Yeah. Definitely. I think if there were better, more evidence-based tests than yes I think we will definitely use them more.." [ED Paeds Consultant 5]

"I guess you'll have to do some research into that in terms of how to validate that…because sometimes I feel like a lot of the research…are actually done on…adults rather than children." [Trainee 7]

Failure to resolve diagnostic uncertainty and alter clinical management

"I don't think any of the POCT necessarily give you a diagnosis unless you've got urine dipstick that is positive for urine infection. Umm, so I don't think they really change your diagnosis as such." [Trainee 2]

"So I think that if I'm at the point where I want more information and I want to know what the CRP, I automatically want to observe them for a few hours, so it doesn't actually make that much of a difference to my working practice because I put less emphasis on the test than I do on the observations. ." [Trainee 9]

"I want something that's a binary. Cause I think what you might find with a lot of tests, it is a lot of grey. And it will give you an answer that, it could be a virus, it could be a bacteria; we're not really that sure. In which case it's useless as another blood test. Quicker, but otherwise no better. Cause that's what our CRP, our white cells show, and we are still left in the situation.." [Gen Paeds Consultant 2]

"I would be far more in favour of a test that could predict the likelihood of significant deterioration rather than one that could tell me whether something is viral or bacterial.." [ED Paeds Consultant 4]

"So, if you take an NPA [nasopharyngeal aspirate], which is how we test for viral respiratory panels here, you testing for 10 viruses. If you only take the RSV rapid test, you are only testing for RSV. And you still need to get a sample from the child. If you are getting a sample then why not just get the whole thing?" [Trainee 5]

"I guess the thing that I would want to know is that: are they likely to change my management here and how?…in a way that is going to be clinically significant. So, for example, a throat swab, if I'm going to prescribe antibiotics and the throat swab confirmed that then that is

For peer review only - http://bmjopen.bmj.com/site/about/guidelines.xhtml

great, but that hasn't really changed anything." [ED Paeds Consultant 4]

Preferential reliance on clinical acumen

"Just the basic clinical skills that doctors...I think POCTs supplement what we do as doctors rather than replace it. I don't think they can be used in isolation." [ED Paeds Consultant 2]

"If I wasn't worried, I would trust my judgement because sometimes if you do further test to reassure a parent and it's not needed, you are putting a child through invasive tests, but you are potentially going to find something that might not be helpful in the bigger picture…Where I can, I like to manage on my clinical judgement in looking at the child and my observational skills." [ED Paeds Consultant 2]

"Like I said, I think basically a lot of what I do is based on history and examination...the essential skills that doctors need to know. I trust my history and examination quite a bit." [Trainee 10]

For peer review only - http://bmjopen.bmj.com/site/about/guidelines.xhtml

Supplementary materials

Supplement 1: Doctor’s topic guide

Supplement 2: Sampling matrix

Supplement 3: Initial coding lists

Supplement 4: Example of summary matrix

Supplement 5: POCTs available at project site hospitals

Supplement 6: Measures in place to ensure safe and efficient use of POCTs in the two hospitals

For peer review only

For peer review only - http://bmjopen.bmj.com/site/about/guidelines.xhtml

Document version: 15.0
Document date 02/07/18
IRAS ID: 248723

Topic Guide (Clinicians): Perceptions of Hospital-based Healthcare Providers in England on the Use of Rapid Diagnostic Tests (RDTs)/Point of Care Tests (POCTs) in Febrile Infants, A Qualitative Study

LONDON SCHOOL of HYGIENE &TROPICAL MEDICINE

Participant ID Number:	Gender: Male / Female
Date (DD/MM/YY):	

Patient Information Sheet & Informed Consent:

- Overview and Purpose of Study
- Aims of Interview and Expected Duration
- Who is Involved
- Why Participant's Involvement is Important, Advantages and Disadvantages
- What Will Happen to the Results of This Study
- Questions?
- Consent Form

Before I ask you further questions, here is what we will be covering in today's interview

- To explore the medical decision-making process of healthcare providers
- Focus on the availability and roles of RDTs/POCTs in the medical decision making process
- To explore advantages/disadvantages of RDTs/POCTs vs laboratory-based versions of the tests
- Implications of using RDTs/POCTs aside from the medical decision making process
- Facilitators/barriers to the wider adoption of POCTs in assessing febrile children
- To identify characteristics in future diagnostics in managing febrile children

For peer review only - http://bmjopen.bmj.com/site/about/guidelines.xhtml

Document version: 15.0
Document date 02/07/18
IRAS ID: 248723

Warm-up and Demographic Information: May I ask you some details about you and your background as a healthcare provider?

Job Title	
For how long have you been working in the hospital?	
Year of graduation from medical/nursing school	

Now I would like to ask you some questions about your experiences and perceptions managing febrile children

Aim	Topics & Prompts	Examples/Clarifications
To explore the medical decision-making process of healthcare providers	**How would you manage this patient?**<br>A 4-month-old infant presents with fever, asymptomatic otherwise, clinical examination unremarkable. The infant was inconsolable all morning, but currently settles with mum and feeding well, 3 non-bilious, milky vomits, vitals are within normal limits. Mum says he never vomits after feeds.<br><br><u>Prompts:</u><br>• What factors influence your decision to:<br>○ Discharge the patient home or admit into the hospital?<br>○ Use/not use antibiotics?<br>○ Use diagnostic tests? If yes, which tests? And why?	• *RDTs/POCTs are defined as diagnostic tests that can be completed at the patient's bedside and results are typically available within 15-20 minutes.*<br>• *We are focusing specifically on RDTs/POCTs used to investigate infections in children.*

For peer review only

Document version: 15.0
Document date 02/07/18
IRAS ID: 248723

Focusing down on the availability and role of RDTs/POCTs in the medical decision- making process	*I understand that in this hospital the RDTs/POCTs that are available are* (SHOW CARD)<br><br>**Are these RDTs/POCTs available in your A&E department for assessing febrile children?**<br><br>**Do you use any of these tests?**<br>**(if yes)**<br>• **In what cases/presentations would you use RDTs/POCTs?**<br>• **What exact purpose do they serve?**<br><br>• **Do you think these tests should be used for these purposes? Please elaborate.**<br><br>**How do RDTs/POCTs impact your initial working diagnosis?**<br>• **Do they change your initial working diagnosis?**<br>• **Are there circumstances where do they do not change your initial working diagnosis?**<br><br>**(if no)**<br>• **Why don't you use any RDTs/POCTs?**<br><br>**In some places where CRP RDTs/POCTs have been introduced, providers sometimes still prescribe antibiotics despite testing negative, what could be the reasons for this?**<br><br>**How would you rate yourself: are you a frequent user of diagnostics in general? Of RDTs/POCTs? Why is that?**<br><br>**Are there other RDTs/POCTs you would like to have available?**	• *For example: Are the test used to identify children who:*<br>○ *Need immediate care (triage)*<br>○ *Need additional confirmatory tests (screening)*<br>○ *Should be gathered (cohorted)*<br><br>• *For example, referring to the initial case scenario with a 4 month old febrile child and a rapid CRP test yielding 5 mg/L.*

For peer review only

Document version: 15.0
Document date 02/07/18
IRAS ID: 248723

To explore the perceived advantages and disadvantages of RDTs/POCTs and lab-based versions of the tests	**What do you consider to be the advantages and disadvantages about current RDTs/POCTs compared to their laboratory-based equivalents?**<br><br>**Are the tests used instead of equivalent lab tests (to decide, for example, on hospital admission or prescription of antibiotics?)**	• *Examples of RDTs/POCTs and lab equivalent:*<br>○ *Urine dipstick vs. microscopy*<br>• *What about:*<br>○ ***Need for training (is this factor a disadvantage?)***<br>○ ***Parental acceptance***<br>○ ***Route of obtaining sample***<br>○ *Time to get results*<br>○ *Costs*<br>○ *The fact that some clinicians find useful to have a delay between ordering the test and getting results, to allow watchful observation of the clinical evolution*

For peer review only

Document version: 15.0
Document date 02/07/18
IRAS ID: 248723

To explore wider implications of using RDTs/POCTs, aside from the medical decision-making process	**What are the expectations of parents when they present to hospital with a febrile child?**<br><br>Prompts<br>• With regards to:<br>○ the use of diagnostic tests? (e.g. do parents ask you for these tests?)<br>○ prescription of antibiotic?<br><br>• **How do these expectations impact on your decision to use RDTs/POCTs?**<br><br>Prompts<br>(ST. MARY'S ONLY – If participant says no)<br>• **If CRP RDTs/POCTs were available to you how would parental expectations affect your decision to use the tests?**<br><br>**Do you think there is a high risk of litigation in the UK? How does it affect your use of diagnostics and RDTs/POCTs?**<br><br>**Does the use of RDTs/POCTs have an impact on your relationship with parents and children?**<br><br>Prompts<br>• If CRP RDT/POCT were available to you, would it have an impact on your relationship with parents and children? If yes, please describe it to me.	

For peer review only

Document version: 15.0
Document date 02/07/18
IRAS ID: 248723

	**What other factors influence you on deciding whether to use RDTs/POCTs in children?**<br><br>**How do RDTs/POCTs fit within the consultation process?**<br><br>**What is the influence of other colleagues using the tests; are there influential colleagues? Who are they?**	• ***What about the role of guidelines?***<br>• ***What about previous adverse/memorable cases/difficult cases?***<br><br>*For example:*<br>• **Is the use of RDTs/POCTs disruptive in your routine clinical practice?**<br>• *What is the impact on patient flow?*<br>• *What happens whilst waiting for results?*<br>• *Who decides to request and administer the tests (e.g. nurses, doctors)?*<br>• *Who is responsible for acting on tests results?*

For peer review only

For peer review only - http://bmjopen.bmj.com/site/about/guidelines.xhtml

Document version: 15.0
Document date 02/07/18
IRAS ID: 248723

To identify facilitator(s) and barrier(s) to the wider adoption of POCTs for use in febrile children	**In your workplace, who decides on the introduction of new diagnostic tests?**<br><br>**What is or could be your role in deciding whether or not to introduce a new test?**<br><br>**What helps or hinders the adoption of additional POCTs in your work place?**<br><br>Prompts:<br>• Do you think barriers, if any, should be overcome?<br>• If yes, how could they be overcome?	• For example:<br>• A facilitator would be to have several devices so people do not need to queue up to use the POCT.<br>• A barrier would be if patients need to pay out of pocket for the test to be used<br>• What about accessibility, time pressure, accuracy of the POCTs? Staff factors? Cost factors? Hospital factors? Patient factors? Training? Physical Space? Other implications of introducing RDTs/POCTs in your workplace?
	**Some RCTs/POCTs are used more widely in other countries. Why are these used less in the UK despite being approved for use?**<br><br>**In your department, what is your perception of the clinical culture towards RDTs/POCT technology? Can you elaborate? By whom?**<br><br>**If you were given more autonomy in your practice at the A&E, do you think you would use more/less RDTs/POCTs? (Nurses)**<br><br>**Does your department have some autonomy from the hospital to decide on the introduction of RDTs/POCTS?**	• For example, CRP POCT is used more in the Netherlands.<br><br>• For example: open vs. conservative<br><br>• Autonomy: Orders for certain tests do not require a clinician to sign off on or administer.

For peer review only - http://bmjopen.bmj.com/site/about/guidelines.xhtml

Document version: 15.0
Document date 02/07/18
IRAS ID: 248723

To identify characteristics of future diagnostics for the management of febrile children	**Is there a need for novel diagnostic tests (POCTs or non-POCTs) that would help you in the management of febrile children?**<br><br>**For future RDTs/POCTs, what characteristics need to be improved to make you want to use them?**	• *What about:*<br>○ *Distinguishing between viral and bacterial infections*<br>○ *Predicting risk of developing severe disease*<br>○ *Differentiate between gram negative and gram positive infections*<br>○ *Identify bacterial species*<br>○ ***What is the maximum turnaround time for results you would find acceptable?***<br>○ ***What type of sample (blood, saliva, urine)?***

Closing: Is there anything else that you think is important about using diagnostic tests in the management of febrile children that we have not talked about?

- Summarize Covered Domains
- Any Other Questions/Concerns?
- Ensure participant has copies of patient information sheet and consent forms; provide any additional information as needed
- Thank interviewee for their input and participation.

For peer review only

Supplement 2: Sampling Matrix

**Healthcare workers**	**Hospital Site**		**Total**
	SMH, London	GNCH, Newcastle	
Nurses - Paediatric Emergency Department	2-3	2-3	4-6
Consultants - Paediatric Emergency Department	2-3	2-3	4-6
Consultants – General Paediatrics	2-3	2-3	4-6
Trainee doctors - Paediatrics*	5-6	5-6	10-12
**Total**	11-15	11-15	**22-30**

SMH: St Mary's Hospital London
GNCH: Great North Children's Hospital Newcastle

For peer review only

Supplement 3: Initial coding lists

**Emerging theme**	**Codes**	
	**Researcher: QL**	**Researcher: EL**
1. Trust in Clinical Judgement Over Diagnostics	RDTs not in isolation of acumen	Results to justify preliminary clinical judgements
	clinical acumen valued	Clinical examination/impression still more trusted
	assessment-dependent decision	Severity of clinical symptoms & signs
	Trust in clinical judgement	Decision to treat still dependent on clinical presentation
	Importance of examination and presentation	
	Tests and observations as clinical tools	
2. Perceived limitations of current POCTs	Worry for "grey" results	Clinical examination/impression still more trusted
	Concerns for interpreting results	CRP lag in elevation
	Time-consuming to obtain sample (i.e., urine dip)	Decision to treat still dependent on clinical presentation
	Machine need calibration	Results ambiguous/contradictory to initial impression
	Repeating test for ambiguous results	Difficult to interpret/use
	Cycle of repeat testing	"Grey areas", intermediate values
	Machine breaks down	Does not give overall diagnostic assessment of patient's condition
	Waiting for results	Forms "part of the picture" only
	Losing sample	
	Forgetting to input results	
	Concerns for falsely reassuring parents	
	'Treat the person, not the number'	
	Need for clinical picture (whole picture)	
3. Implementation of POCTs		
3a. Facilitators to introduction	Need clear guidelines for patient management	Increase acceptability for parents/child
	Need for evidence	
	Staff Buy-In	
	Need for staff education	
	Need for staff awareness	
	Need for training	

For peer review only

	Need training for test interpretation	
	Expanding nurses' roles	
	Nurse autonomy for RDTs	
	Need for nurse-led RDTs	
	RDTs for first point of patient contact	
	Need training to run test	
3b. Barriers to introduction	Fear of overreliance	Not actually that much faster than labs
	Overuse due to ease	Not as much information as labs
	False Positives	Results not as reliable/accurate as labs
	Fear of Overtreating	Over-reliance/use of POCTs
	Over-reassurance	False reassurances for clinicians and parents
	Fear use will undermine clinical judgement	Deterioration of clinical skills and judgement
	Concern for test quality control	Potential for overtreatment of patients
	High Staff turn-over	
	Difficult to coordinate training	
4. Current Roles of POCTs		
4a. Clinical management/Diagnostic Roles	Quick Rule-Out Tool (urine dip)	Used to monitor progress/response to treatment
	Aid antibiotic decision	Results clearly indicate potential diagnosis
	Prevent antibiotic misuse	Results to justify preliminary clinical judgements
	Reassurance for holding off antibiotics	Results support clinician's judgement
	Aid Clinicians’ Stewardship Role	
	Prevent rapid deterioration	
	Aid Justification of antibiotics (urine dip)	
4b. Hospital Logistics/Practical Roles	Speediness (in general)	Reduce patient waiting times
	Speediness of results	Allows faster clinical decision making
	Speediness of decision-making	Avoids sampling inconveniences of lab-tests
	Quicker admission and discharge time	Patient cohorting to appropriate beds/cubicles
	Alleviate A&E pressure	
	Less wait time for parents	
	Reduce observation period	
	Quick status check in wards (blood gas, potential for CRP)	
	Quick bed allocation (cohorting) (RSV)	
4c. Parent-clinician communication role	Reassurance for Parents	Improves communication between parents and doctors
	Parental expectations of diagnosis and treatment	Parents want evidence from "a test"
	Aid in explaining decision-making	Results reduce parental anxiety

For peer review only - http://bmjopen.bmj.com/site/about/guidelines.xhtml

	Parental anxiety may influence RDT usage	When don't parents trust a clinician's judgement
	RDTs role to decrease parental anxiety	Parents do not care what test is used
	Address parents' "fever-phobia"	
	RDT as reassurance	
	RDT use decision: a 'middle ground'	
	Decisions based on collaboration	
5. Determinants of Current POCT Usage	Parental expectations of diagnosis and treatment	Clinical decisions not influenced by fear of litigation
	Parental anxiety may influence RDT usage	Clinical decisions influenced by fear of litigation
	Decisions based on collaboration	Clinical decisions not influenced by parental expectations
	RDT use decision: a 'middle ground'	Clinical decisions influenced by parental expectations
	Colleague's Influence	Want evidence from "a test"
	Junior doctors Use More RDTs	Parental Concerns
	Decrease in frequency with seniority (doctors)	Young age of patient
	Tools for less experienced (doctors)	Unclear infectious foci
	Increase in frequency with autonomy (nurse)	
6. Characteristics of Future POCTs		
6a. Performance properties	Need to know reliability and specificity	More information from one POCT
	Importance of PPV and NPV	Results should make a difference in clinical course/management
	Need for test to change management	Should match gold-standard of lab-tests
	Need for decreasing ambiguity	Provides additional/different information from lab tests
6b. Practical use properties	Easy to act on binary results	Easy to interpret results
		Positive or negative results
		Numeric thresholds are ambiguous
		Need for analysers
		Minimal amounts of sample needed
		Painless/non-invasive for child
		Not time consuming to obtain
		Easier to get than lab-tests

For peer review only

Supplement 4: Example of summary matrix for the emergent theme "Trust in clinical acumen"

Participant	Initial coding	Sub-theme	Quote
ED_C2N	Assessment based decision	Patient management is based more on clinical findings than on diagnostic tests	To be honest with you, ED we really aren't going to get any diagnostic tests back. If the child is unwell, the child is going to be admitted. I might send some baseline inflammatory markers, so full blood count, CRP, Basic electrolytes, obviously blood cultures if their child is very febrile or if I'm concerned that they are shocked, urine sample. But beyond that, most of my management is going to be clinical grounds rather than treating the test result.
ED_N3N	Assessment based decision	Patient management is based more on clinical findings than on diagnostic tests	Even if the CRP was 5, that wouldn't mean you wouldn't then go on and do a septic screen. So it very much depends on the baby and how he looks and how he changes because...they change very very quickly.
ED_C1L	Assessment-based decision, RDT not in isolation	Diagnostic tests are just a complement of clinical assessment, they should not be used in isolation	So just the importance of making sure that it's still all about clinical assessment, and test are just an additional thing to that, that are really valuable and helpful.
ED_C1L	clinical acumen valued	Diagnostic tests are just a complement of clinical assessment, they should not be used in isolation	And inflammatory markers are a guide, but you know, we can get caught out where inflammatory markers can be low, and the child can still be very unwell. So it depends on the whole clinical acumen thing.
ED_C2L	Clinical acumen valued	?	I think you kind of go off clinically on what the case looks like.
ED_J2L	Clinical acumen valued	Patient management is based more on clinical findings than on diagnostic tests	...I'd prefer to be clinically led cuz if I was saying there was a number, CRP or the white count, which I may worry about sepsis. Then it could be argued that shouldn't you be worried even if those numbers are normal given that we know that the sensitive is particularly early in serious infection.
ED_N3N	Clinical acumen valued	Patient management is based more on clinical findings than on diagnostic tests	To obviously get the cultures and some things like that, and make sure we start them on sensitive to the particular infection they had. But, it would be a clinical determination on how unwell the child was and whether or not we were worried about them. It might put us a little direction on what the focus of the infection.
ED_J1N	Importance of examination and presentation	Patient management is based more on clinical findings than on diagnostic tests	I suppose there is always what you are seeing...you shouldn't be treating the numbers...you should be treating the patient so there is always a discrepancy between what you are seeing in your patient and your results. [J1N]
ED_C1L	Importance of examination and presentation	Patient management is based more on clinical findings than on diagnostic tests	Yeah so, first of all, it would be on the history and examination and what he looks like in front of me.
ED_N1L	Importance of examination and presentation	Sicker children can be identified clinically	And, it's usually, you're more aware of those children at triage, because usually the sicker children are having very abnormal obs. You can their respiratory efforts are a lot more increased. So you can usually differentiate between those children, roughly know that child...you know...this baby may need watching for 24 hours in our CDU, or it may need admission to the ward. You can usually differentiate between those two.
ED_J1N	Importance of examination and presentation	Patient management is based more on clinical findings than on diagnostic tests	Then I'd concentrate on my history and examination more than any other tests at that point.

ED_J1N	Importance of examination and presentation	Patient management is based more on clinical findings than on diagnostic tests	I think I always base the majority of my differential diagnosis on the history and examination really in a child like that.
ED_J1N	Importance of examination and presentation	Patient management is based more on clinical findings than on diagnostic tests	like I said, I think basically a lot of what I do is based on history and examination...the essential skills that doctors need to know. I trust my history and examination quite a bit.
ED_C1L	RDT not used in Isolation	Diagnostic tests are just a complement of clinical assessment, they should not be used in isolation	So I think clinical picture is still the most valuable tool and we have to make sure that people are trained in recognition of the unwell child, and use tests to augment them.
ED_C2L	RDT not used in Isolation	Diagnostic tests are just a complement of clinical assessment, they should not be used in isolation	people try to see CRP so they would use it as a kind of tracking rather than a stand alone. So, to be honest, I've not seen that many people treating anybody based on the CRP without some kind of clinical reasoning for doing so. If the patient looks unwell, you'd treat them.
ED_J1N	RDT not used in Isolation	Diagnostic tests are just a complement of clinical assessment, they should not be used in isolation	But I think if we do bloods, it is always together with history and examination just to aid your decision making. I don't think it makes your decision.
ED_C1N	RDT not used in Isolation	Diagnostic tests are just a complement of clinical assessment, they should not be used in isolation	Umm...yeah, I think that they can't be taken in isolation and I think that as long as they are used along with clinical judgement and after their assessment of the patient, taking into account all of the other factors such as their observations, their examination, the history that you've got, the parental concern, I think they are all very useful to make a clinical decision.
ED_C1N	RDT not used in Isolation	Diagnostic tests are just a complement of clinical assessment, they should not be used in isolation	Just the basic clinical skills that doctors...I think POCTs supplement what we do as doctors rather than replace it. I don't think they can be used in isolation.
ED_C1L	RDTs not used in Isolation	Diagnostic tests are just a complement of clinical assessment, they should not be used in isolation	I think that they can't be taken in isolation. I think that as long as they are used along with clinical judgement. And after their assessment of the patient. And taking into account all of the other factors such as their observations, their examination, the history that you've got, the parental concern. I think they are all very useful to make a clinical decision.
ED_C1L	RDTs not used in Isolation	Diagnostic tests are an alternative or complement of clinical assessment	So I think, you know, that's great that they are easy to do and that we get results fairly quickly but to think of the use of them in the same context where we happen to actually bleed a child...so we can use our clinical acumen as well.
ED_N2N	RDTs not used in Isolation	Diagnostic tests are just a complement of clinical assessment, they should not be used in isolation	A clinical reason to use it or if you are concerned about the patient and how they look. Then, just do the tests that are necessary so that you know you've made the right decision.
ED_C1N	RDTs not used in Isolation	Diagnostic tests are just a complement of clinical assessment, they should not be used in isolation	I don't think disruptive. Umm...as long as it's taken into context and that it's not taken in isolation.
ED_C1L	Tests and Observations (paired) as clinical tools	Clinical observation is used as a diagnostic tool	But we use that a lot. Any child with a fever without a very good focus. And even sometimes if they've got a respiratory tract focus, you might still want to make sure that we are...and if its clear. Often it's good to wait for them to wait for them to wee anyway. If there's a story of...umm....reduced fluid intake.

ED_C1L	Tests and Observations (paired) as clinical tools	Clinical observation is used as a diagnostic tool	what the point of doing the test is, explain what the results are, and say it's kind of here or there. And then, if the...again, if you are clinically worried, you would keep them for observation but maybe not treat.
ED_C1L	Tests and Observations (paired) as clinical tools	Clinical observation is used as a diagnostic tool	I can't remember exactly but maybe the CRP was 20-30. And then something else. But they were persistently tachycardic, but the urine was clear. And we do a chest x-ray and that was clear. And there was no meningitis or anything like that. And, the child didn't look septic or unwell. Just tachycardic. And in the end, we just observed them without treating them, and the tachycardic settled and then they went home. So, sometimes with pediatrics particularly, think, you just need a bit more time and such things.
ED_C1L	Tests and Observations (paired) as clinical tools	Clinical observation is used as a diagnostic tool	Regardless of how obvious the actual diagnosis is. So I think to help augment it, that would be really valuable though cuz it would help you maybe filter out some of those ambic [ambulatory] kids that you would admit but could actually could go home. You know, but then, actually, period observation is a good active treatment I think in pediatrics. I
ED_C2L	Tests and Observations (paired) as clinical tools	Clinical observation is used as a diagnostic tool	RDTs get people moving through quicker. Yeah. But I think in peds, we accept that our flow is often a bit disruptive. Cuz in pediatrics especially, there is quite a big role for a period of observation especially in febrile children.
ED_J1L	Tests and Observations (paired) as clinical tools	Clinical assessment and observation are more valuable than CRP	So I think that if I'm at the point where I want more information and I want to know what the CRP, I automatically want to observe them for a few hours, so it doesn't actually make that much of a difference to my working practice because I put less emphasis on the test than I do on the observations.
ED_J1N	Tests and Observations (paired) as clinical tools	Clinical observation is used as a diagnostic tool	So the child is usually just waiting with the parents. Sometimes it's useful because we can just observe them and see how they go for their fever or if we are waiting for anything else which is the urine sample or a fluid challenge. So sometimes, it doesn't affect the length of stay in the hospital cuz we were observing them anyway.
ED_C1N	Tests and Observations (paired) as clinical tools	Diagnostic tests are an alternative or complement of clinical assessment	So I think having useful tests and then documentation of how they have presented to you is essential. And whether that's the POCTs or observations and clinically document them in the notes the findings and what you've discussed.
ED_C2L	Trust in clinical judgement	Patient management is based more on clinical findings than on diagnostic tests	if they didn't, it would go more on how well they looked clinically.
ED_C1N	Trust in clinical judgement	Patient management is based more on clinical findings than on diagnostic tests	Where I can, I like to manage on my clinical judgement in looking at the child and my observational skills.
ED_C2L	Trust in clinical judgement	Patient management is based more on clinical findings than on diagnostic tests	But again, I don't find CRP that useful in that way, cuz I found that most people would go clinically on how the patient is like.
ED_J1N	Trust in clinical judgement	Patient management is based more on clinical findings than on diagnostic tests	I think you should base it on your clinical skills rather than investigating.

For peer review only

ED_C1N	Trust in clinical judgement	Patient management is based more on clinical findings than on diagnostic tests	I think it's good to use the test, but I think it doesn't replace clinical judgement and thorough clinical assessment.
ED_C1N	Trust in clinical judgement	Patient management is based more on clinical findings than on diagnostic tests	I think RDTs if you're concerned as a clinician, if even a test negative you should act on that concern.
ED_C1L	Trust in clinical judgement, RDT not used in isolation	Diagnostic tests are just a complement of clinical assessment, they should not be used in isolation	I guess, I try to base my differential diagnosis on clinical...on history and examination first. And if it's really clear, the POCTs augment what your management plan is going to do anyway. Cuz you're just going to treat based on that anyway.
ED_C1L	Trust in clinical judgement, RDT not used in isolation	Patient management is based more on clinical findings than on diagnostic tests	I guess anyway you go with your clinical picture,
CON1N	Clinical examination/impression still more trusted	Patient management is based more on clinical findings than on diagnostic tests	I think if we came back to that one, even if the CRP was low on POCT, I think I would still act because I know that, I know all the time that a lab CRP can be done and they can be low and we still get signs of an infection
SHO1L	Clinical examination/impression still more trusted	Clinical assessment and observation are more valuable than a POCT	And actually, I think the feeling is that having two hours with the patient is often more valuable than the result that you get back because you just get the time to see things for a bit longer, and just assess that baby or that child a few more times. […] I don't want to be able to see a child and send them home in 10 minutes, because I'll probably miss something. I'd much prefer if I saw a child and saw them for two hours, and pop my head in a few times and then have some blood results back that reassures me to let the child go home.
REG2N	Clinical examination/impression still more trusted	Clinical assessment and observation are more valuable than a POCT	I don't think so. Ummm, I think probably because on what I've touched on before. So I think the febrile children that you see fall into a few categories. So I think you get the children who are febrile and there is, you find a focus to their infection and based on that it's, it's normally, you're normally able to say if it's going to be more viral and kind of self-limiting, or this is more likely to be bacterial and you'd give the appropriate antibiotics depending on where the infection was. Then you get children who you can't find a focus but they look well. And if you've got a child who exams well with normal obs, then I would tend to do a period of observations, or if they look completely well, home with clear safety net advice for the parents to bring back if they became more unwell. Or I think you get the children who look sick and look septic, and in those children, you are going to treat with antibiotics. I was thinking, I couldn't think of like specific groups of children where a POCT would like really help you, help me decide on whether to give antibiotics or not.

Supplement 5: POCTs availability at the study hospitals

Hospital	Test	Vendor
St. Mary's Hospital, London	Blood gas analyser	GEM 4000
	Blood glucose/ketones	Freestyle Precision Pro
	Urinalysis	Siemens Multistix 8 SG
	CRP*	Alere Afinion AS100
Great North Children's Hospital, Newcastle	Urine dipstick	Combur7 test
	Urine ketones	Ketodiastix Ascensia
	Urine microscopy	
	Blood glucose	Accucheck inform II
	Blood gas analyser	GEM premier 5000
	Influenza & RSV	Cepheid GeneXpert

*CRP POCTs were not available at St. Mary's Hospital at the time of study, however they previously had been available as part of a pilot

For peer review only

Supplement 6: Measures in place to ensure safe and efficient use of POCTs in the two hospitals

All point of care testing is overseen by a POCT committee that supervises point of care testing across the NHS trusts to which the two hospitals included in the study belong: Newcastle upon Tyne Hospitals NHS Foundation Trust with regards Great North Children Hospital in Newcastle, and Imperial College Healthcare NHS trust with regard St Mary's Hospital in London.

Within the two trusts, point of care testing is subject to strict governance and must be performed to the same quality standards as all testing undertaken within the United Kingdom Accreditation Service (UKAS) accredited laboratory departments, and to the same standards of the MHRA (Medicines and Healthcare products Regulatory Agency), the NHS Litigation Authority (NHSLA), the International Organisation for Standardisation (ISO), the Royal College of Pathologists (RCPath), and the Clinical Pathology Accreditation (CPA) standards.

Chaired by the POCT Manager, the POCT committee:

- Leads and coordinates POCT activities for the Trust in conjunction with the Trust's key objectives
- Maintains training records of all POCT operators throughout the Trust and coordinates staff education and facilitates training for all POCT devices. All POCT operators must be accredited by the POCT committee by undertaking POCT recognised training prior to patient testing. Competence in using POCT devices is achieved via Initial Training – this constitutes face-to-face training by either attending a timetabled training session or with a recognised 'Cascade Trainer'. Refresher Training is required every subsequent two years to maintain that competency
- Coordinates the provision of External Quality Assurance (EQA) schemes where possible, review EQA performance and offer advice on the improvement of poor performance
- Provides advice and help with documentation to Managers of areas on policies, operating procedures, training and competencies
- Undertakes Risk assessments for POCT devices where appropriate
- Leads and coordinates the validation of new POCT devices before implementation
- Provides evaluation of the performance of POCT equipment
- Coordinates audits and inspections to provide Trust assurance of quality patient care and value for money

For peer review only

BMJ Open

The role of Point-of-Care-Tests in the management of febrile children: A qualitative study of hospital-based doctors and nurses in England

Journal:	*BMJ Open*
Manuscript ID	bmjopen-2020-044510.R2
Article Type:	Original research
Date Submitted by the Author:	30-Mar-2021
Complete List of Authors:	Li, Edmond; Imperial College London Faculty of Medicine, Surgery & Cancer; London School of Hygiene and Tropical Medicine Faculty of Infectious and Tropical Diseases, Clinical Research Department<br>Dewez, Juan ; London School of Hygiene and Tropical Medicine, Clinical Research Department<br>Luu, Queena; London School of Hygiene and Tropical Medicine Faculty of Infectious and Tropical Diseases, Clinical Research Department<br>Emonts, Marieke; Great North Children's Hospital, Paediatric Immunology, Infectious Diseases & Allergy<br>Maconochie, Ian; Imperial College Healthcare NHS Trust, St Mary's Hospital, Paediatric Emergency Department<br>Nijman, R; Imperial College Healthcare NHS Trust, Paediatric Emergency Medicine; Imperial College London Faculty of Medicine, Department of Infectious Disease<br>Yeung, Shunmay; London School of Hygiene and Tropical Medicine Faculty of Infectious and Tropical Diseases, Clinical Research Department; Imperial College Healthcare NHS Trust, St Mary's Hospital, Department of Paediatrics
**Primary Subject Heading**:	Paediatrics
Secondary Subject Heading:	Emergency medicine, Infectious diseases, Diagnostics
Keywords:	QUALITATIVE RESEARCH, INFECTIOUS DISEASES, Paediatric A&E and ambulatory care < PAEDIATRICS, PAEDIATRICS, Paediatric infectious disease & immunisation < PAEDIATRICS, Diagnostic microbiology < INFECTIOUS DISEASES

SCHOLARONE™
Manuscripts

I, the Submitting Author has the right to grant and does grant on behalf of all authors of the Work (as defined in the below author licence), an exclusive licence and/or a non-exclusive licence for contributions from authors who are: i) UK Crown employees; ii) where BMJ has agreed a CC-BY licence shall apply, and/or iii) in accordance with the terms applicable for US Federal Government officers or employees acting as part of their official duties; on a worldwide, perpetual, irrevocable, royalty-free basis to BMJ Publishing Group Ltd ("BMJ") its licensees and where the relevant Journal is co-owned by BMJ to the co-owners of the Journal, to publish the Work in this journal and any other BMJ products and to exploit all rights, as set out in our licence.

The Submitting Author accepts and understands that any supply made under these terms is made by BMJ to the Submitting Author unless you are acting as an employee on behalf of your employer or a postgraduate student of an affiliated institution which is paying any applicable article publishing charge ("APC") for Open Access articles. Where the Submitting Author wishes to make the Work available on an Open Access basis (and intends to pay the relevant APC), the terms of reuse of such Open Access shall be governed by a Creative Commons licence – details of these licences and which Creative Commons licence will apply to this Work are set out in our licence referred to above.

Other than as permitted in any relevant BMJ Author's Self Archiving Policies, I confirm this Work has not been accepted for publication elsewhere, is not being considered for publication elsewhere and does not duplicate material already published. I confirm all authors consent to publication of this Work and authorise the granting of this licence.

For peer review only

The role of Point-of-Care-Tests in the management of febrile children: A qualitative study of hospital-based doctors and nurses in England

AUTHORS

Edmond C. Li,[1,2*] Juan E. Dewez,[1*] Queena F. Luu,[1*] Marieke Emonts,[3,4] Ian Maconochie,[5] Ruud Nijman,[5] Shunmay Yeung[1,6]

[*]Contributed equally

AFFILIATION

1: Clinical Research Department, Faculty of Infectious and Tropical Diseases, London School of Hygiene & Tropical Medicine, London, United Kingdom

2: Patient Safety Translational Research Centre, Department of Surgery and Cancer, Faculty of Medicine, Imperial College London, London, United Kingdom

3: Great North Children's Hospital, Paediatric Immunology, Infectious Diseases & Allergy, Newcastle upon Tyne Hospitals NHS Foundation Trust, Newcastle upon Tyne, United Kingdom

4: Translational and Clinical Research Institute, Newcastle University, Newcastle upon Tyne, United Kingdom

5: Section of Paediatric Infectious Diseases, Faculty of Medicine, Imperial College, London, United Kingdom

6: Department of Paediatric Infectious Disease, St Mary's Imperial College Hospital London, United Kingdom

For peer review only - http://bmjopen.bmj.com/site/about/guidelines.xhtml

For peer review only

CORRESPONDING AUTHOR

Shunmay Yeung, Keppel Street, London WC1E 7HT, United Kingdom

Email: shunmay.yeung@lshtm.ac.uk

Phone: +44 2079272657

ABSTRACT

Objectives

The use of rapid point-of-care tests (POCTs) has been advocated for improving patient management and outcomes and for optimising antibiotic prescribing. However, few studies have explored healthcare workers' views about their use in febrile children. The aim of this study was to explore the perceptions of hospital-based doctors and nurses regarding the use of POCTs in England.

Study design

Qualitative in-depth interviews with purposively selected hospital doctors and nurses. Data were analysed thematically.

Setting

Two university teaching hospitals in London and Newcastle.

Participants

24 participants (paediatricians, emergency-department doctors, trainee paediatricians, and nurses).

Results

There were diverse views about the use of POCTs in febrile children. The reported advantages included their ease of use and the rapid availability of results. They were seen to contribute to faster clinical decision-making; the targeting of antibiotic use; improvements in patient care, flow and monitoring; cohorting (ie the physical clustering of hospitalised patients with the same infection to limit spread); and enhancing communication with parents. These advantages were less evident when the turnaround for

For peer review only

results of laboratory tests was 1-2 hours. Factors such as clinical experience and specialty, as well as the availability of guidelines recommending POCT use, were also perceived as influential. However, in addition to their perceived inaccuracy, participants were concerned about POCTs not resolving diagnostic uncertainty or altering clinical management, leading to a commonly expressed preference for relying on clinical skills rather than test results solely.

Conclusion

In this study conducted in two university teaching hospitals in England, participants expressed mixed opinions about the utility of current POCTs in the management of febrile children. Understanding the current clinical decision-making process, and the specific needs and preferences of clinicians in different settings will be critical in ensuring the optimal design and deployment of current and future tests.

Strengths & limitations of this study

Strengths

- A specific focus on the paediatric population.
- The inclusion of two hospitals in two different settings.
- A multi-disciplinary and diverse group of participants including nurses and doctors with different levels of clinical experience and training.

Limitations

- The study took place in university teaching hospitals and did not explore the views of clinicians in other settings.
- The study explored the views of participants on several POCTs used in febrile children, limiting the amount of detail obtained about specific POCTs.

FUNDING DECLARATION

For peer review only - http://bmjopen.bmj.com/site/about/guidelines.xhtml

JED, ME, and SY are supported by PERFORM, a consortium funded by the European Union's Horizon 2020 programme, under grant agreement No. 668303. RGN was supported by NIHR Academic clinical fellowship (CF- 2015-21-016) and lectureship (CL-2018-21-007) award programme. The funding bodies did not take part in the design of the study and data collection, and did not take part in the data analysis and interpretation of results. The funding bodies did not take part in the writing of the manuscript.

COMPETING INTERESTS

None declared

DATA SHARING

The datasets generated and/or analysed during the current study will be made available upon reasonable request through the London School of Hygiene and Tropical Medicine data repository (LSHTM Data COMPASS).

WORD COUNT: 3407

For peer review only

For peer review only - http://bmjopen.bmj.com/site/about/guidelines.xhtml

INTRODUCTION

Febrile illness is one of the most common reasons for children to present to hospital, and managing it can often be challenging.[1,2] The majority of febrile children will have self-limiting infections which can be safely managed at home, often without antibiotics.[3-5] However, a few will have potentially severe infections and identifying these children from the others can be difficult, as clinical symptoms and signs are often non-specific in children.[3] Consequently, they may be prescribed antibiotics that are not needed, subjected to invasive tests, and admitted for monitoring whilst awaiting microbiology results.[4,5] As well as the direct impact this may have on children and parents in terms of distress, costs, and inconvenience,[6] there may also be indirect impacts in terms of antimicrobial resistance (AMR).[7]

The World Health Organization advocates the use of rapid point-of-care tests (POCTs) to reduce the use of antibiotics.[8] POCTs have the potential to aid clinical decision-making, limit the use of other invasive tests, and improve the use of medical resources in general.[9] However, achieving these impacts depends on several factors, including whether POCTs are taken up by healthcare workers and how they influence clinical decisions.[10] This cannot be assumed. For example, when malaria POCTs were introduced, many clinicians continued to prescribe antimalarials despite patients testing negative.[11]

The types of POCTs that are currently available for the management of childhood infections include urine dipsticks; rapid tests for Group A Streptococcal throat infections ("rapid strep test"); rapid respiratory virus tests for Respiratory Syncytial Virus (RSV) or Influenza; and blood-based tests including C-reactive protein (CRP) and blood gas analysers.

To date research exploring the views of clinicians, on the use of POCTs for managing patients with infections, have predominantly focused on general practitioners (GPs) treating adult patients.[12-19] To the best of our knowledge, only one study pertains to the perceptions of hospital-based healthcare workers regarding the use of POCTs in children.[20]

For peer review only - http://bmjopen.bmj.com/site/about/guidelines.xhtml

The aim of this study was to explore the experiences and attitudes of hospital-based doctors and nurses regarding the use of POCTs in children with acute infections, and to identify factors that influence their use.

METHODS

Qualitative in-depth interviews with doctors and nurses were conducted to better understand their experiences and attitudes regarding POCT use in children with suspected infection.[21] Ontologically, our approach adhered to subtle realism, and epistemologically to interpretivism,[22,23] because we believed that although reality (i.e., the use of diagnostics) exists independently of our beliefs, the interpretation of participants' perceptions and how they make sense of their experiences were needed to ascertain what influences POCT usage.

The research team comprised of two public health professionals and five paediatricians with experience in qualitative methods. Two topic guides, one for doctors and one for nurses, were developed based on a review of the literature (Supplement 1). These were piloted with two paediatricians and two nurses at St Mary's Hospital in London. The pilot interviews were not included in the analysis. The topic guides were iteratively adapted during data collection after regular debriefings between EL, QL, and JED to discuss main findings, refine questions and explore new areas of inquiry.[24]

Eligible participants comprised of doctors and nurses with responsibility for making decisions about whether or not to use POCTs on febrile children in two university teaching hospitals: the Great North Children's Hospital in Newcastle, and St Mary's Hospital in London. We purposively chose not to interview other sub-specialty paediatricians such as paediatric infectious disease specialists as they are less often responsible for decisions on POCT use in the acute hospital setting. Purposive maximum variation sampling was used, focusing on participants with different clinical roles and varying years of experience. A sampling matrix was developed specifying the targeted number of participants of each

For peer review only - http://bmjopen.bmj.com/site/about/guidelines.xhtml

healthcare worker category (consultant paediatrician, paediatric trainee with three or more years training, paediatric trainees with less than three years training, and ED nurses) (Supplement 2). As members of staff of the two hospitals, SY, RN, IM, ME contacted available paediatric staff who fit the inclusion criteria to ascertain their interest in being interviewed for the study. Those who agreed, were then followed-up by the two interviewers (EL, male) and (QL, female) who were not known to the participants, and who provided the detailed participant information sheet, obtained written informed consent, and arranged the interview date. The study aimed to include 22-30 participants, a sample size which is in line with other qualitative studies investigating healthcare workers' perceptions, and was expected to allow for data saturation.[12,13,18,25] Identifying the actual point of data saturation was important to make best use of the available time and resources and to minimise the disruption caused by diverting healthcare providers from their normal clinical activities. During the regular debriefings, EL, QL, and JED identified whether any new ideas had been raised. Once no new ideas had emerged from the last several interviews, the authors agreed that data saturation had been reached. One-on-one audio-recorded interviews took place between June and August 2018 during normal work hours at the respondents' workplace. Either EL or QL conducted the interview while the other took field notes.

The interview records were transcribed verbatim by EL and QL and back-checked against the recording and field notes for accuracy. The transcripts were annonymised (only participants' job titles were documented) and then analysed thematically.[24] The analysis was both deductive (i.e., partly based on pre-existing knowledge) and inductive (i.e., additional concepts were identified from the dataset). EL and QL generated two initial lists of coding from the dataset through line-by-line coding (Supplement 3). EL, QL, and JED then agreed on an analytical framework comprising the most relevant codes and used the framework to re-code the entire dataset. Matrices were used to display data related to initial themes (Supplement 4). Iterative exploration of the data identified patterns within the dataset and continued until no new themes were identified. Further refinement was carried out with the rest of the

For peer review only - http://bmjopen.bmj.com/site/about/guidelines.xhtml

research team to ensure themes were internally coherent and distinctive to produce clear descriptions and explanations of each theme.

Ethical approval was obtained from the London School of Hygiene & Tropical Medicine Ethics Committee (Ref: 15040-15088). Health Research Authority approval (Ref: IRAS 248723) and administrative approval were obtained from the Imperial College Healthcare NHS Trusts (Ref: 18SM4662) and Newcastle Upon Tyne Hospital NHS Foundation (Ref: 248723). The COREQ checklist has been completed (Supplement 5)

Patient and public involvement

Patients and members of the public were not involved in the design of this study.

RESULTS

A total of 24 out of 27 invited healthcare workers were interviewed (Table 1); two nurses and one trainee doctor did not reply to the invitation. Interviews lasted for 60-75 minutes. Most participants fully engaged with the interviews and readily shared their views on the topics of interest with little direction from the interviewers. Participants' experience of using POCTs varied at the two hospitals. Although all participants reported routine use of urinary dipsticks, Respiratory Syncytial Virus (RSV) rapid tests, and blood gas tests, some participants from St Mary's Hospital recalled also having access to CRP and rapid strep tests in past studies. (Please see Supplement 6 and 7 for description of POCT availability and oversight at the two hospitals).

A range of reasons was given for and against using POCT. These have been grouped into eight themes (Table 2): five pertaining to factors encouraging the use of POCTs (Box 1) and three for factors discouraging the use of these tests (Box 2) in the management of febrile children.

Factors encouraging the use of rapid POCTs

For peer review only - http://bmjopen.bmj.com/site/about/guidelines.xhtml

Most participants were keen on using POCTs due to their inherent advantages (Box 1). One of the main cited advantages was their ease of use and speed with which they can provide results when compared to hospital laboratory-based diagnostics. This was seen to contribute to accelerating decision-making and improving patient flow in busy wards. However, a few participants questioned the magnitude of this impact and noted that these advantages were largely context dependent. In circumstances where laboratories were able to return results within one to two hours, some felt that the potential benefit in terms of the rapidity of POCTs result was outweighed by the perceived greater accuracy of laboratory-based tests, the possibility to monitor the patient's clinical evolution, and the flexibility to see other patients in parallel whilst awaiting laboratory results.

Urine dipsticks and CRP POCTs were reported to support quicker decision-making on whether or not to prescribe antibiotics or to perform additional diagnostic tests. Rapid RSV testing was valued for assisting with the timely cohorting of children with respiratory infections, thereby limiting the potential for nosocomial transmission. The ability to use rapid POCTs without the help of other doctors or nurses, and to get results independently from the laboratory, was important for many participants, because it allowed for easier repeat testing for monitoring purposes and provided participants with a greater sense of direct control over the care of their patients. Blood gas POCTs were a notable example of this. Additionally, obtaining results rapidly allowed clinicians to quickly communicate results to parents which helped them to justify clinical decisions and to reduce overall parental anxieties, which was felt to have contributed to improved caregivers' experience. Finally, a noted benefit of blood-based POCTs was that they could be performed on finger-prick blood rather than requiring venepuncture which was seen as more invasive and difficult to perform. This contributed to their acceptability not only to clinicians but also to children and their parents.Aside from the described advantages inherent to POCTs, there were also other factors that influenced the use of POCTs. For doctors, their level of experience appeared to influence their use of diagnostics. There was a prevailing perception that less experienced clinicians used

For peer review only - http://bmjopen.bmj.com/site/about/guidelines.xhtml

more POCTs as the tests served as learning tools, as well as a "safety net" if their clinical acumen proved inadequate. Moreover, participants also alluded that diagnostics use, including POCTs, varied according to their perceptions of the risk for severe infections, which itself was dependent on the clinician's specialty training and the population seeking care. Some participants believed that general practitioners (GPs) used less diagnostics than emergency department paediatricians, who in turn use less diagnostics than paediatric infectious diseases specialists. This was thought to be because the patient population seen by the three types of clinicians differed, with GPs seeing proportionally fewer children with severe infections.

Finally, clinical guidelines were also seen as important. Participants reported that guidelines including recommendations on the use of the tests and the interpretation of results in children would be highly influential in their decision to use POCTs. This was because guidelines were perceived as both providing reassurance of best practices and protection against malpractice.

Factors discouraging the use of rapid POCTs

Several participants were concerned about the accuracy of the test results (Box 2). It was commonly perceived that there were discrepancies in relation to the patient's clinical presentation and/or when compared to laboratory tests. These concerns undermined clinicians' trust in POCTs, and often resulted in subsequent confirmatory laboratory tests to be performed prior to making a clinical decision. Many also cited the lack of strong evidence supporting the accuracy of some POCTs for paediatric use, some noting that existing POCTs such as CRP had predominantly only been validated in adults.

Another notable reason for hesitancy about some POCTs was their perceived inability to adequately address diagnostic uncertainty. Some participants mentioned that with current POCTs that provide numerical results (e.g. CRP levels) instead of binary results ("positive/negative"), the tests were unable to provide a definitive clinical answer when results were within an "intermediate" range. Other

For peer review only - http://bmjopen.bmj.com/site/about/guidelines.xhtml

participants felt that current POCTs were not able to reliably predict which initially well-appearing febrile children were at risk of clinical deterioration and therefore required hospitalisation. This was perceived to be an important feature of diagnostics for managing fever in children. It was also commonly noted that some POCTs were only designed to detect a single pathogen such as RSV, which was of limited utility compared to the range of different pathogen that were detectable from laboratory-based panels. Altogether, this failure to resolve diagnostic uncertainty was seen to limit the potential for POCTs to alter clinical decisions. In addition, even when POCTs were perceived as accurate, it was felt their results might not prove consequential enough to warrant a change in the patient's overall clinical management. An example of this was the rapid Group A strep test. While the test was acknowledged to be sensitive and specific, some participants felt that they would make a decision to prescribe antibiotics irrespective of whether a test confirmed the presence Group A streptococcus or not. However, the results of other POCTs such as urine dipsticks, greatly influenced clinical management for many participants.

Overall, in view of the perceived limitations of POCTs, participants expressed a preference for prioritising their clinical skills (history, examination, observation of patients) and acumen over the reliance on diagnostics in guiding their clinical management of febrile children.

Comparison of views between types of participants

The views expressed by different types of participants were broadly similar. However, there was a trend towards some differences emerging, mainly between trainees versus consultants and nurses. Specifically consultants stressed the importance of relying on their clinical acumen, more so than trainees. They also felt that trainees used more POCTs, a sentiment was also shared by nurses, but this was seldom reported by trainees. Trainees more often mentioned how CRP results can appear normal for a child at the beginning of a serious infection, whilst consultants seemed more concerned with the difficulty of

For peer review only

For peer review only - http://bmjopen.bmj.com/site/about/guidelines.xhtml

interpreting intermediate CRP results. The potential contribution of POCT usage in improving antibiotic stewardship was mentioned widely but generally with stronger emphasis by consultants and nurses than trainees. Finally, nurses mentioned their willingness to perform more POCTs independently of doctors in the future. Whilst consultants and trainees rarely commented on who should be performing the tests, when they did, they were not against nurses using POCTs independently.

DISCUSSION

This study provides insight into what hospital-based doctors and nurses in England think of POCTs in relation to managing children with suspected infection, a topic on which little has previously been published. Within the two hospitals, we interviewed a diverse range of healthcare providers who have responsibility for deciding on the use of POCTs including both consultants in general paediatrics and emergency paediatrics, paediatric trainees with different levels of experience, and nurses. A range of reasons for and against the use of POCTs was identified.

The advantages of POCTs over laboratory-based tests in terms of their ease of use and the rapid availability of results were widely recognised. Other reported potential benefits included faster clinical decision-making; improved monitoring of patients; more targeted use of antibiotics; enhanced patient flow; the rapid cohorting of inpatients with respiratory infection; and improved communication with parents. However, these advantages were less evident when laboratories were able to return results within a few hours. Being able to perform blood-based POCTs on finger-prick samples was also seen as less invasive and therefore more acceptable to clinicians, parents, and children compared to tests requiring venepuncture. Guidelines were also perceived to be important in guiding patient management, including the use of POCTs.

However, participants also mentioned a number of important limitations of POCTs. These include their perceived inaccuracy, their inability to predict risks of clinical deterioration, and the limited scope of diagnostic information from certain tests. Some POCTs were not seen as being able to solve diagnostic

For peer review only - http://bmjopen.bmj.com/site/about/guidelines.xhtml

For peer review only

uncertainty and others were perceived as having a limited role in guiding or changing overall clinical management. Altogether, these perceived shortcomings reinforced the overarching view that clinicians should trust their clinical acumen more than diagnostics.

The only other study that we were aware of which specifically explored the views of paediatricians and nurses about rapid POCTs usage in children, found similar results. Participants valued their ease and speed of use, which enabled early treatment, cohorting, decreasing antibiotic prescription, and better communication with parents.[20] Some participants were also concerned about the accuracy of the tests and about the limited changes in clinical management enabled by POCTs.

Some studies exploring the views of GPs reported similar advantages and concerns, especially regarding the accuracy of tests.[12,14,20,25,26,27] However, there were conflicting results in terms of the perceived invasiveness of POCTs: two studies found that GPs would not use blood-based POCTs because they felt finger pricking was too invasive in children,[12,25] while another study reported that GPs, perceived the pain to be short-lived and recounted that children were actually interested by the use of rapid tests during consultations.[28] Other GPs felt it was easy to persuade parents that antibiotics were not needed compared to adult patients, limiting the need to use POCTs for this purpose.[25] Some GPs reportedly also feared that the use of POCTs could actually increase antibiotic prescription. [27,28] Finally, in contrast to our finding that for some paediatricians the perceived limitations of POCTs reinforced their preference of relying on their clinical acumen over diagnostics, some of the studies with GPs reported that there were concerns that the perceived advantages of POCTs could lead to the tests being over adopted and used, which could potentially undermine clinical acumen in the long term. [13,14,27]

Of note, national and local paediatric guidelines were seen to be important in guiding patient management, including the use of diagnostics. At the time of the study, the use of urine dipstick and blood gas analysis were mentioned in relevant guidelines for managing children with suspected

For peer review only - http://bmjopen.bmj.com/site/about/guidelines.xhtml

infections. These guidelines were widely available in hospital acute care settings in England. However, to date, neither CRP POCT nor rapid strep tests have been recommended for the management of acute childhood infections by the National Institute for Health and Care Excellence (NICE), and neither were available in the two hospitals at the time of this study.

The study had some limitations, and also highlighted a number of important areas for further research. Firstly, as the study aimed to explore the role of POCTs used in febrile children rather than focusing on one or two specific tests, it was sometimes not possible to decipher whether the participants' comments were referring to specific POCTs or POCTs in general. Given the substantial differences in the attributes and roles of different POCTs, deeper exploration about specific POCTs would be helpful.

Secondly, several members of our research team are active paediatricians with varying experience of POCTs. However, we have trained and worked in wide variety of countries and settings, and hold a wide range of views about POCTs ourselves. Moreover, two public health specialists with limited experience in diagnostics or paediatrics research conducted the interviews, contributed to the data analysis, interpretation, and drafting of results. Therefore, although as in any qualitative research, there is an inherent risk of subjectivity in the interpretation and presentation of the results, we believe that the breadth of views and backgrounds in the team have helped to minimise this risk.

Thirdly, the scope of this study was restricted to two teaching hospitals in England and did not capture the views of paediatricians in district general hospitals, nor general practitioners. Further qualitative research with other key stakeholders, such as clinicians in other sub-specialties and those based in district general hospitals or in the community, and with parents and children themselves, would be very informative. As the study was limited to England, we could not make any comparisons with other countries where the culture of clinical decision-making and availability of diagnostic testing may differ. As the use of POCTs seem to vary greatly across countries, it would be very interesting to carry out similar studies elsewhere. It would also be useful to elucidate in which patients and where within the

For peer review only

For peer review only - http://bmjopen.bmj.com/site/about/guidelines.xhtml

care pathway, specific POCTs would have the most impact. Finally, this study was carried out in the pre-COVID-19 era. Since then, the diagnostics landscape has changed dramatically; it would informative to carry out a similar study now, as the views about POCT use may likely have evolved since.

Conclusion

In this study conducted in two university teaching hospitals in England, participants expressed mixed opinions about the utility of current POCTs in the management of febrile children. Whilst the many potential advantages of POCTs were well recognised, concerns about their accuracy and failure to solve diagnostic uncertainty and alter clinical management, limited the perceived utility of current POCTs in this setting. Understanding the clinical decision-making process and the concerns and needs of clinicians in different settings, will be critical in the development of new tests and in evaluating the potential impact on clinical management and other outcomes. Further studies in different countries and health care settings in the post-COVID-19 era, should help to inform this understanding.

AUTHOR CONTRIBUTIONS

Juan Emmanuel Dewez (JED), Edmond C. Li (EL), and Queena F. Luu (QL) contributed equally to this paper. JED and Shunmay Yeung (SY) conceived of the study. SY had overall responsibility. All authors input into the design of the study and the study materials. Marieke Emonts (ME), Ian Maconochie (IM), Ruud G Nijman (RGN) and SY identified participants. EL and QL conducted and transcribed the interviews. EL, and QL analysed the data and drafted the manuscript under JED’s supervision. EL, JED, and SY made substantial revisions. All authors provided input into drafts of the manuscript and agree on the contents of the final version.

ACKNOWLEDGEMENTS

For peer review only

The authors wish to thank Emma Lim for her support, and for all the participants who enthusiastically contributed their valuable time and thoughts to this research project.

For peer review only

For peer review only - http://bmjopen.bmj.com/site/about/guidelines.xhtml

340 **REFERENCES**

1. Sands R, Shanmugavadivel D, Stephenson T, Wood D. Medical problems presenting to paediatric emergency departments: 10 years on. Emergency medicine journal : EMJ. 2012 May;29(5):379-82.
2. Alpern ER, Stanley RM, Gorelick MH, Donaldson A, Knight S, Teach SJ, et al. Epidemiology of a pediatric emergency medicine research network: the PECARN Core Data Project. Pediatric emergency care. 2006 Oct;22(10):689-99.
3. Van den Bruel A, Haj-Hassan T, Thompson M, Buntinx F, Mant D. Diagnostic value of clinical features at presentation to identify serious infection in children in developed countries: a systematic review. Lancet (London, England). 2010;375(9717):834-45.
4. De S, Williams GJ, Hayen A, Macaskill P, McCaskill M, Isaacs D, et al. Accuracy of the "traffic light" clinical decision rule for serious bacterial infections in young children with fever: a retrospective cohort study. BMJ (Clinical research ed). 2013;346:f866.
5. Nijman RG, Vergouwe Y, Thompson M, van Veen M, van Meurs AHJ, van der Lei J, et al. Clinical prediction model to aid emergency doctors managing febrile children at risk of serious bacterial infections: diagnostic study. BMJ : British Medical Journal. 2013;346.
6. Leigh S, Grant A, Murray N, Faragher B, Desai H, Dolan S, et al. The cost of diagnostic uncertainty: a prospective economic analysis of febrile children attending an NHS emergency department. BMC medicine. 2019;17(1):48.
7. European Centre for Disease Control, European Food Safety A, European Medicines A. ECDC/EFSA/EMA second joint report on the integrated analysis of the consumption of antimicrobial agents and occurrence of antimicrobial resistance in bacteria from humans and food-producing animals. EFSA Journal. 2017;15(7):e04872-n/a.
8. WHO. World Health Organization. Global action plan on antimicrobial resistance 2015. Available from http://www.who.int/antimicrobial-resistance/global-action-plan/en. Accessed 20/12/17.
9. Larsson A, Greig-Pylypczuk R, Huisman A. The state of point-of-care testing: a European perspective. Upsala journal of medical sciences. 2015;120(1):1-10.
10. Horvath AR, Lord SJ, StJohn A, Sandberg S, Cobbaert CM, Lorenz S, et al. From biomarkers to medical tests: the changing landscape of test evaluation. Clinica chimica acta; international journal of clinical chemistry. 2014;427:49-57.
11. Burchett HE, Leurent B, Baiden F, Baltzell K, Bjorkman A, Bruxvoort K, et al. Improving prescribing practices with rapid diagnostic tests (RDTs): synthesis of 10 studies to explore reasons for variation in malaria RDT uptake and adherence. BMJ Open. 2017;7(3):e012973.
12. Butler CC, Simpson S, Wood F. General practitioners' perceptions of introducing near-patient testing for common infections into routine primary care: a qualitative study. Scandinavian journal of primary health care. 2008;26(1):17-21.

For peer review only - http://bmjopen.bmj.com/site/about/guidelines.xhtml

13. Cals JW, Chappin FH, Hopstaken RM, van Leeuwen ME, Hood K, Butler CC, et al. C-reactive protein point-of-care testing for lower respiratory tract infections: a qualitative evaluation of experiences by GPs. Family practice. 2010;27(2):212-8.

14. Wood F, Brookes-Howell L, Hood K, Cooper L, Verheij T, Goossens H, et al. A multi-country qualitative study of clinicians' and patients' views on point of care tests for lower respiratory tract infection. Family practice. 2011;28(6):661-9.

15. Pulcini C, Pauvif L, Paraponaris A, Verger P, Ventelou B. Perceptions and attitudes of French general practitioners towards rapid antigen diagnostic tests in acute pharyngitis using a randomized case vignette study. Journal of Antimicrobial Chemotherapy. 2012;67(6):1540-6.

16. Leydon GM, McDermott L, Moore M, Williamson I, Hobbs FD, Lambton T, et al. A qualitative study of GP, NP and patient views about the use of rapid streptococcal antigen detection tests (RADTs) in primary care: 'swamped with sore throats?'. BMJ open. 2013;3(4).

17. Hardy V, Thompson M, Alto W, Keppel GA, Hornecker J, Linares A, et al. Exploring the barriers and facilitators to use of point of care tests in family medicine clinics in the United States. BMC family practice. 2016;17(1):149.

18. Hardy V, Thompson M, Keppel GA, Alto W, Dirac MA, Neher J, et al. Qualitative study of primary care clinicians' views on point-of-care testing for C-reactive protein for acute respiratory tract infections in family medicine. BMJ open. 2017;7(1):e012503.

19. Eley CV, Sharma A, Lecky DM, Lee H, McNulty CAM. Qualitative study to explore the views of general practice staff on the use of point-of-care C reactive protein testing for the management of lower respiratory tract infections in routine general practice in England. BMJ open. 2018;8(10):e023925.

20. Bustinduy AL, Jeyaratnam D, Douthwaite S, Tonkin-Crine S, Shaw R, Hyrapetian L, et al. Point-of-care tests for infectious diseases: barriers to implementation across three London teaching hospitals. Acta paediatrica (Oslo, Norway : 1992). 2017.

21. Flick U. An introduction to qualitative research. 5th ed. SAGE Publications; 2014.

22. Ritchie J, Lewis J, Nicholls CMN, Ormston R. Qualitative Research Practice: A Guide for Social Science Students and Researchers: SAGE Publications; 2013.

23. Durrand MAC, Tracy. (2014) Principles of social research . 2nd ed. Maidenhead: Open University Press.

24. Braun V, Clarke V. Using thematic analysis in psychology. Qualitative Research in Psychology. 2006;3(2):77-101.

25. Schot MJ, Broekhuizen BD, Cals JW, Brussee E, de Wit NJ, Verheij TJ, et al. C-reactive protein point-of-care testing in children with cough: qualitative study of GPs' perceptions. BJGP Open. 2018;1(4):bjgpopen17X101193.

For peer review only

For peer review only - http://bmjopen.bmj.com/site/about/guidelines.xhtml

26. Jones CH, Glogowska M, Locock L, Lasserson DS. Embedding new technologies in practice - a normalization process theory study of point of care testing. BMC health services research. 2016;16(1):591.

27. Turner PJ, Van den Bruel A, Jones CHD, Plüddemann A, Heneghan C, Thompson MJ, et al. Point-of-care testing in UK primary care: a survey to establish clinical needs. Family practice. 2016;33(4):388-94.

28. Van den Bruel A, Jones C, Thompson M, Mant D. C-reactive protein point-of-care testing in acutely ill children: a mixed methods study in primary care. Archives of disease in childhood. 2016 Apr;101(4):382-5.

341

For peer review only

TABLES

Table 1. Number of different types of health care workers interviewed

Healthcare workers	Hospital Site		Total
	SMH, London	GNCH, Newcastle	
Nurses – Paediatric Emergency Department	1	3	4
Consultants – Paediatric Emergency Department	3	2	5
Consultants – General Paediatrics	2	1	3
Trainee doctors – Senior House Officers	4	3	7
Trainee doctors – Registrars	2	3	5
**Total**	12	12	**24**

SMH: St Mary's Hospital London
GNCH: Great North Children's Hospital Newcastle

For peer review only

Table 2. Summary of themes

FACTORS ENCOURAGING USE OF POCTS

Advantages

- Ease of use and rapid availability of test results (leading to faster decision making and enhanced patient flow)
- Improved clinical care (resulting from targeted antibiotic treatment, facilitated patient cohorting, closer monitoring, and improved communication with caregivers)
- Minimal test invasiveness

Other factors

- Being less experienced
- Being a paediatric Infectious Disease specialist > general paediatrician> GP
- Guidelines recommending their use

FACTORS DISCOURAGING USE OF POCTS

- Concerns over test accuracy
- Failure to resolve diagnostic uncertainty and alter clinical management

For peer review only - http://bmjopen.bmj.com/site/about/guidelines.xhtml

- Preferential reliance on clinical skills and acumen

344

Box 1. Direct quotes about the factors that encourage the use of rapid POCTs

ADVANTAGES

Ease of use and rapid availability of test results

"If it's something the nurses could do really easily and quickly then it would help again, the flow and performance through the department. So let's say it takes 20 minutes to wee [make urinate] a child and it takes a specialist, a nurse, and the doctor...the nurse holding and etc, and you've got a really busy queue, and if you've got a really easy finger prick test that a nurse can do." [ED Paeds Consultant 1]

"Because it [POCTs in general] would just make things a lot quicker. Decision making a lot quicker. Being seen by doctors a lot quicker." [Nurse 3]

"So, you still might sit on the fence and want to admit and observe but it helps you just maybe make that decision a little bit quicker." [ED Paeds Consultant 1]

"So speed is probably the main advantage really and by virtue of being less people involved, it's a bit more accessible. You can also do them yourself, so you don't necessarily have to wait for other people to do the test." [Trainee 8]

Improved clinical management of febrile children

a) More targeted antibiotic treatment

"So that [urine dipstick] might change your choice of...not only whether you would give them antibiotics, but also what antibiotics you would give." [Trainee 9]

"I think that [POCTS in general] would help us with our overprescribing but also help reassure us that we are finding the ones that do need antibiotics and getting them to them [the children]." [ED Paeds Consultant 2]

"...I think bloods is probably one of the bigger ones...it just gives a much better indication as to whether or not we would treat with antibiotics or what...like...what kind of route you would go down..." [Nurse 3]

b) Deciding cohorting and disposition of patient

"The RSV test, we primarily..., are used for children who are being admitted. So we don't use it as a diagnostic test per se for children in the ED. But they use it for cohorting children if they are admitted to the ward." [ED Paeds Consultant 5]

"You know that kid where you are a little bit unsure, then the CRP come back a 5. And you were kinda thinking that it was probably ok, then it would influence a bit. Whereas the CRP

For peer review only - http://bmjopen.bmj.com/site/about/guidelines.xhtml

came back at 60 or 100, then you be like, right they definitely need to come in (be hospitalised) and that sort of stuff." [ED Paeds Consultant 1]

"With the urine [dipstick], I think we're determining whether they need other tests really and whether we need to screen further or look for other sources of infection." [ED Paeds Consultant 2]

c) *Closer monitoring for clinical changes*

"It's the repeat tests that I think that's the bigger advantage that they allow more easy repeat so you can assess the response to your treatment [...] I think that is the single biggest advantage 'cause it gives you the quantification of the effect of your treatment." [ED Paeds Consultant 4]

"CRP (...), I think it is useful for monitoring if you've got a patient that is not following the usual course. Like if you got a drop in CRP and a raise again. I think it's very useful" [ED Paeds Consultant 2]

d) *Improved communication with caregivers*

"Parents find it quite stressful on how long like something bloods can take. They often come in and ask like how long it's going to be until my bloods are back. At least with the urine and the blood gas, you can kind of tell them fairly instantly. Like put their mind at rest and things." [Nurse 1]

"I think parents put great faith in technology. If you have a CRP that is negative, then it's negative. And if it's positive, then it's positive. So I think it's much more black and white way. And so, I think...it can be very convincing for them. So I think it can be helpful in that way." [Trainee 9]

"I think if somebody [parents] wanted antibiotics and I was sure they didn't [need] and the [rapid] CRP was there, I would be tempted, even though I thought know that it's not necessary evidence based to do a POCT, and say your CRP is only 6, you don't need antibiotics. [ED Paeds Consultant 5]

Minimal test invasiveness

"I might use that 'cause it's easy to do whereas I wouldn't want to do a proper venous blood test on a child because it's a painful procedure. A child would tolerate a little finger-prick, not like it, but they will tolerate it. Whereas a proper blood test involves lots of screaming, time, big needle, waiting around for hours." [Gen Paeds Consultant 2]

"Just thinking about, managing a sweaty toddler whose heart can be tachycardic who I think is probably going to be ok, and I'm struggling to get bloods, but if I could do a finger prick and I can get results in ten minutes, if it was a CRP of 100, I could take my time that we've got access and reassure the parents of why that was whereas if it was ten, I'd say well alright, we don't have to do bloods, we're just going to keep you here for a few hours and see how you

For peer review only - http://bmjopen.bmj.com/site/about/guidelines.xhtml

are...so I think that would be helpful." [ED Paeds Consultant1]

OTHER FACTORS

Training, experience, and specialty

"When you are a junior doctor you do a bit of more investigations and then you build a bit more of a profile and you get a feel for whether children are more unwell or not. [As a senior,] you probably tend to use less investigations." [ED Paeds Consultant 1]

"We would have a tendency in the A&E to do more investigations than a GP would...it's all about what you are used...what population that you see. So obviously, if you are an ID [infectious diseases] consultant, then you're seeing all the weird and wonderful so all the other stuff is filtered out by parents and GP and then by A&E and so the population that they see is different." [ED Paeds Consultant 1]

"If I had a point of care available for other things, I would use it. I would use a CRP, an FBC, I mean I would use everything if I can get it instantly from a finger-prick size sample. I know there is a feeling that probably when you get more senior, that you would sometimes you don't want the information from a blood test but at my level, I would..." [Trainee_5]

Guidelines

"And so, I think we are very influenced by guidelines because we feel securities with guidelines. And so, to do a POCT, I would do it [if it is in the guidelines] because if I don't do it and it goes wrong, how will I defend myself. And essentially in fever, I already feel vulnerable." [Trainee 9]

"So I think with a POCT around fever, then you have to have a guideline about how you are gonna use the results. Because especially things like CRP, everyone is going to interpret CRP differently. (...) And so it would have to be a part of a guideline on how to interpret the results." [Trainee 9]

"If the guideline says to do...if there was a well-respected guideline in place for a particularly clinical scenario that says you should use this test, then of course, I would expect we would have to have a very good reason of why we aren't doing that test." [ED Paeds Consultant 4]

345

346

For peer review only - http://bmjopen.bmj.com/site/about/guidelines.xhtml

Box 2. Factors discouraging the use of rapid POCT

Concerns over test accuracy

"It may still be a viral infection and you have a borderline CRP and it may mean you have to go on and do further tests." [ED Paeds Consultant 2]

"There is a perception that they [POCTs] are less reliable, so often, there's a follow up with the formal lab equivalent…if you are shown a strong evidence base for accuracy of each POCT test, that would be critical for adoption." [Trainee 11]

"… if there was one test that could tell me if it wasn't bacterial infection, then yup. Fantastic. Something better than CRP. Yeah, it'd definitely be useful for that. If there was a viral diagnostic test that could tell me every single virus. Absolutely. Yeah. Definitely. I think if there were better, more evidence-based tests than yes I think we will definitely use them more.." [ED Paeds Consultant 5]

"I guess you'll have to do some research into that in terms of how to validate that…because sometimes I feel like a lot of the research…are actually done on…adults rather than children." [Trainee 7]

Failure to resolve diagnostic uncertainty and alter clinical management

"I don't think any of the POCT necessarily give you a diagnosis unless you've got urine dipstick that is positive for urine infection. Umm, so I don't think they really change your diagnosis as such." [Trainee 2]

"So I think that if I'm at the point where I want more information and I want to know what the CRP, I automatically want to observe them for a few hours, so it doesn't actually make that much of a difference to my working practice because I put less emphasis on the test than I do on the observations. ." [Trainee 9]

"I want something that's a binary. Cause I think what you might find with a lot of tests, it is a lot of grey. And it will give you an answer that, it could be a virus, it could be a bacteria; we're not really that sure. In which case it's useless as another blood test. Quicker, but otherwise no better. Cause that's what our CRP, our white cells show, and we are still left in the situation.." [Gen Paeds Consultant 2]

"I would be far more in favour of a test that could predict the likelihood of significant deterioration rather than one that could tell me whether something is viral or bacterial.." [ED Paeds Consultant 4]

"So, if you take an NPA [nasopharyngeal aspirate], which is how we test for viral respiratory panels here, you testing for 10 viruses. If you only take the RSV rapid test, you are only testing for RSV. And you still need to get a sample from the child. If you are getting a sample then why not just get the whole thing?" [Trainee 5]

"I guess the thing that I would want to know is that: are they likely to change my management here and how?…in a way that is going to be clinically significant. So, for example, a throat swab, if I'm going to prescribe antibiotics and the throat swab confirmed that then that is

For peer review only - http://bmjopen.bmj.com/site/about/guidelines.xhtml

great, but that hasn't really changed anything." [ED Paeds Consultant 4]

Preferential reliance on clinical acumen

"Just the basic clinical skills that doctors...I think POCTs supplement what we do as doctors rather than replace it. I don't think they can be used in isolation." [ED Paeds Consultant 2]

"If I wasn't worried, I would trust my judgement because sometimes if you do further test to reassure a parent and it's not needed, you are putting a child through invasive tests, but you are potentially going to find something that might not be helpful in the bigger picture...Where I can, I like to manage on my clinical judgement in looking at the child and my observational skills." [ED Paeds Consultant 2]

"Like I said, I think basically a lot of what I do is based on history and examination...the essential skills that doctors need to know. I trust my history and examination quite a bit." [Trainee 10]

For peer review only - http://bmjopen.bmj.com/site/about/guidelines.xhtml

Supplementary materials

Supplement 1: Doctor’s topic guide

Supplement 2: Sampling matrix

Supplement 3: Initial coding lists

Supplement 4: Example of summary matrix

Supplement 5: COREQ checklist

Supplement 6: Measures in place to ensure safe and efficient use of POCTs in the two hospitals

Supplement 7: POCTs available at project site hospitals

For peer review only

For peer review only - http://bmjopen.bmj.com/site/about/guidelines.xhtml

Document version: 15.0
Document date 02/07/18
IRAS ID: 248723

Topic Guide (Clinicians): Perceptions of Hospital-based Healthcare Providers in England on the Use of Rapid Diagnostic Tests (RDTs)/Point of Care Tests (POCTs) in Febrile Infants, A Qualitative Study

Participant ID Number:	Gender: Male / Female
Date (DD/MM/YY):	

Patient Information Sheet & Informed Consent:

- Overview and Purpose of Study
- Aims of Interview and Expected Duration
- Who is Involved
- Why Participant's Involvement is Important, Advantages and Disadvantages
- What Will Happen to the Results of This Study
- Questions?
- Consent Form

Before I ask you further questions, here is what we will be covering in today's interview

- To explore the medical decision-making process of healthcare providers
- Focus on the availability and roles of RDTs/POCTs in the medical decision making process
- To explore advantages/disadvantages of RDTs/POCTs vs laboratory-based versions of the tests
- Implications of using RDTs/POCTs aside from the medical decision making process
- Facilitators/barriers to the wider adoption of POCTs in assessing febrile children
- To identify characteristics in future diagnostics in managing febrile children

For peer review only

Document version: 15.0
Document date 02/07/18
IRAS ID: 248723

Warm-up and Demographic Information: May I ask you some details about you and your background as a healthcare provider?

Job Title	
For how long have you been working in the hospital?	
Year of graduation from medical/nursing school	

Now I would like to ask you some questions about your experiences and perceptions managing febrile children

**Aim**	**Topics & Prompts**	**Examples/Clarifications**
To explore the medical decision-making process of healthcare providers	**How would you manage this patient?**<br>A 4-month-old infant presents with fever, asymptomatic otherwise, clinical examination unremarkable. The infant was inconsolable all morning, but currently settles with mum and feeding well, 3 non-bilious, milky vomits, vitals are within normal limits. Mum says he never vomits after feeds.<br><br><u>Prompts:</u><br>• What factors influence your decision to:<br>○ Discharge the patient home or admit into the hospital?<br>○ Use/not use antibiotics?<br>○ Use diagnostic tests? If yes, which tests? And why?	• *RDTs/POCTs are defined as diagnostic tests that can be completed at the patient's bedside and results are typically available within 15-20 minutes.*<br>• *We are focusing specifically on RDTs/POCTs used to investigate infections in children.*

For peer review only

Document version: 15.0
Document date 02/07/18
IRAS ID: 248723

Focusing down on the availability and role of RDTs/POCTs in the medical decision- making process	*I understand that in this hospital the RDTs/POCTs that are available are* (SHOW CARD)<br><br>**Are these RDTs/POCTs available in your A&E department for assessing febrile children?**<br><br>**Do you use any of these tests?**<br>**(if yes)**<br>• **In what cases/presentations would you use RDTs/POCTs?**<br>• **What exact purpose do they serve?**<br><br>• **Do you think these tests should be used for these purposes? Please elaborate.**<br><br>**How do RDTs/POCTs impact your initial working diagnosis?**<br>• **Do they change your initial working diagnosis?**<br>• **Are there circumstances where do they do not change your initial working diagnosis?**<br><br>**(if no)**<br>• **Why don't you use any RDTs/POCTs?**<br><br>**In some places where CRP RDTs/POCTs have been introduced, providers sometimes still prescribe antibiotics despite testing negative, what could be the reasons for this?**<br><br>**How would you rate yourself: are you a frequent user of diagnostics in general? Of RDTs/POCTs? Why is that?**<br><br>**Are there other RDTs/POCTs you would like to have available?**	• *For example: Are the test used to identify children who:*<br>○ *Need immediate care (triage)*<br>○ *Need additional confirmatory tests (screening)*<br>○ *Should be gathered (cohorted)*<br><br>• *For example, referring to the initial case scenario with a 4 month old febrile child and a rapid CRP test yielding 5 mg/L.*

For peer review only

For peer review only - http://bmjopen.bmj.com/site/about/guidelines.xhtml

Document version: 15.0
Document date 02/07/18
IRAS ID: 248723

To explore the perceived advantages and disadvantages of RDTs/POCTs and lab-based versions of the tests	**What do you consider to be the advantages and disadvantages about current RDTs/POCTs compared to their laboratory-based equivalents?**<br><br>**Are the tests used instead of equivalent lab tests (to decide, for example, on hospital admission or prescription of antibiotics?)**	• *Examples of RDTs/POCTs and lab equivalent:*<br>○ *Urine dipstick vs. microscopy*<br>• *What about:*<br>○ ***Need for training (is this factor a disadvantage?)***<br>○ ***Parental acceptance***<br>○ ***Route of obtaining sample***<br>○ *Time to get results*<br>○ *Costs*<br>○ *The fact that some clinicians find useful to have a delay between ordering the test and getting results, to allow watchful observation of the clinical evolution*

For peer review only

Document version: 15.0
Document date 02/07/18
IRAS ID: 248723

To explore wider implications of using RDTs/POCTs, aside from the medical decision-making process	**What are the expectations of parents when they present to hospital with a febrile child?**<br><br>Prompts<br>• With regards to:<br>○ the use of diagnostic tests? (e.g. do parents ask you for these tests?)<br>○ prescription of antibiotic?<br><br>• **How do these expectations impact on your decision to use RDTs/POCTs?**<br><br>Prompts<br>(ST. MARY'S ONLY – If participant says no)<br>• **If CRP RDTs/POCTs were available to you how would parental expectations affect your decision to use the tests?**<br><br>**Do you think there is a high risk of litigation in the UK? How does it affect your use of diagnostics and RDTs/POCTs?**<br><br>**Does the use of RDTs/POCTs have an impact on your relationship with parents and children?**<br><br>Prompts<br>• If CRP RDT/POCT were available to you, would it have an impact on your relationship with parents and children? If yes, please describe it to me.	

For peer review only - http://bmjopen.bmj.com/site/about/guidelines.xhtml

Document version: 15.0
Document date 02/07/18
IRAS ID: 248723

	**What other factors influence you on deciding whether to use RDTs/POCTs in children?**	• ***What about the role of guidelines?***<br>• ***What about previous adverse/memorable cases/difficult cases?***
	**How do RDTs/POCTs fit within the consultation process?**	*For example:*<br>• **Is the use of RDTs/POCTs disruptive in your routine clinical practice?**<br>• *What is the impact on patient flow?*<br>• *What happens whilst waiting for results?*<br>• *Who decides to request and administer the tests (e.g. nurses, doctors)?*<br>• *Who is responsible for acting on tests results?*
	**What is the influence of other colleagues using the tests; are there influential colleagues? Who are they?**	

For peer review only

For peer review only - http://bmjopen.bmj.com/site/about/guidelines.xhtml

Document version: 15.0
Document date 02/07/18
IRAS ID: 248723

To identify facilitator(s) and barrier(s) to the wider adoption of POCTs for use in febrile children	**In your workplace, who decides on the introduction of new diagnostic tests?**<br><br>**What is or could be your role in deciding whether or not to introduce a new test?**<br><br>**What helps or hinders the adoption of additional POCTs in your work place?**<br><br>Prompts:<br>• Do you think barriers, if any, should be overcome?<br>• If yes, how could they be overcome?	• For example:<br>• A facilitator would be to have several devices so people do not need to queue up to use the POCT.<br>• A barrier would be if patients need to pay out of pocket for the test to be used<br>• What about accessibility, time pressure, accuracy of the POCTs? Staff factors? Cost factors? Hospital factors? Patient factors? Training? Physical Space? Other implications of introducing RDTs/POCTs in your workplace?
	**Some RCTs/POCTs are used more widely in other countries. Why are these used less in the UK despite being approved for use?**<br><br>**In your department, what is your perception of the clinical culture towards RDTs/POCT technology? Can you elaborate? By whom?**<br><br>**If you were given more autonomy in your practice at the A&E, do you think you would use more/less RDTs/POCTs? (Nurses)**<br><br>**Does your department have some autonomy from the hospital to decide on the introduction of RDTs/POCTS?**	• For example, CRP POCT is used more in the Netherlands.<br><br>• For example: open vs. conservative<br><br>• Autonomy: Orders for certain tests do not require a clinician to sign off on or administer.

For peer review only - http://bmjopen.bmj.com/site/about/guidelines.xhtml

Document version: 15.0
Document date 02/07/18
IRAS ID: 248723

To identify characteristics of future diagnostics for the management of febrile children	**Is there a need for novel diagnostic tests (POCTs or non-POCTs) that would help you in the management of febrile children?**<br><br>**For future RDTs/POCTs, what characteristics need to be improved to make you want to use them?**	• *What about:*<br>○ *Distinguishing between viral and bacterial infections*<br>○ *Predicting risk of developing severe disease*<br>○ *Differentiate between gram negative and gram positive infections*<br>○ *Identify bacterial species*<br>○ ***What is the maximum turnaround time for results you would find acceptable?***<br>○ ***What type of sample (blood, saliva, urine)?***

Closing: Is there anything else that you think is important about using diagnostic tests in the management of febrile children that we have not talked about?

- Summarize Covered Domains
- Any Other Questions/Concerns?
- Ensure participant has copies of patient information sheet and consent forms; provide any additional information as needed
- Thank interviewee for their input and participation.

For peer review only

Supplement 2: Sampling Matrix

**Healthcare workers**	**Hospital Site**		**Total**
	SMH, London	GNCH, Newcastle	
Nurses - Paediatric Emergency Department	2-3	2-3	4-6
Consultants - Paediatric Emergency Department	2-3	2-3	4-6
Consultants – General Paediatrics	2-3	2-3	4-6
Trainee doctors - Paediatrics*	5-6	5-6	10-12
**Total**	11-15	11-15	**22-30**

SMH: St Mary's Hospital London
GNCH: Great North Children's Hospital Newcastle

For peer review only

Supplement 3: Initial coding lists

Emerging theme	Codes	
	**Researcher: QL**	**Researcher: EL**
1. Trust in Clinical Judgement Over Diagnostics	RDTs not in isolation of acumen	Results to justify preliminary clinical judgements
	clinical acumen valued	Clinical examination/impression still more trusted
	assessment-dependent decision	Severity of clinical symptoms & signs
	Trust in clinical judgement	Decision to treat still dependent on clinical presentation
	Importance of examination and presentation	
	Tests and observations as clinical tools	
2. Perceived limitations of current POCTs	Worry for "grey" results	Clinical examination/impression still more trusted
	Concerns for interpreting results	CRP lag in elevation
	Time-consuming to obtain sample (i.e., urine dip)	Decision to treat still dependent on clinical presentation
	Machine need calibration	Results ambiguous/contradictory to initial impression
	Repeating test for ambiguous results	Difficult to interpret/use
	Cycle of repeat testing	"Grey areas", intermediate values
	Machine breaks down	Does not give overall diagnostic assessment of patient's condition
	Waiting for results	Forms "part of the picture" only
	Losing sample	
	Forgetting to input results	
	Concerns for falsely reassuring parents	
	'Treat the person, not the number'	
	Need for clinical picture (whole picture)	
3. Implementation of POCTs		
3a. Facilitators to introduction	Need clear guidelines for patient management	Increase acceptability for parents/child
	Need for evidence	
	Staff Buy-In	
	Need for staff education	
	Need for staff awareness	
	Need for training	

For peer review only

	Need training for test interpretation	
	Expanding nurses' roles	
	Nurse autonomy for RDTs	
	Need for nurse-led RDTs	
	RDTs for first point of patient contact	
	Need training to run test	
3b. Barriers to introduction	Fear of overreliance	Not actually that much faster than labs
	Overuse due to ease	Not as much information as labs
	False Positives	Results not as reliable/accurate as labs
	Fear of Overtreating	Over-reliance/use of POCTs
	Over-reassurance	False reassurances for clinicians and parents
	Fear use will undermine clinical judgement	Deterioration of clinical skills and judgement
	Concern for test quality control	Potential for overtreatment of patients
	High Staff turn-over	
	Difficult to coordinate training	
4. Current Roles of POCTs		
4a. Clinical management/Diagnostic Roles	Quick Rule-Out Tool (urine dip)	Used to monitor progress/response to treatment
	Aid antibiotic decision	Results clearly indicate potential diagnosis
	Prevent antibiotic misuse	Results to justify preliminary clinical judgements
	Reassurance for holding off antibiotics	Results support clinician's judgement
	Aid Clinicians' Stewardship Role	
	Prevent rapid deterioration	
	Aid Justification of antibiotics (urine dip)	
4b. Hospital Logistics/Practical Roles	Speediness (in general)	Reduce patient waiting times
	Speediness of results	Allows faster clinical decision making
	Speediness of decision-making	Avoids sampling inconveniences of lab-tests
	Quicker admission and discharge time	Patient cohorting to appropriate beds/cubicles
	Alleviate A&E pressure	
	Less wait time for parents	
	Reduce observation period	
	Quick status check in wards (blood gas, potential for CRP)	
	Quick bed allocation (cohorting) (RSV)	
4c. Parent-clinician communication role	Reassurance for Parents	Improves communication between parents and doctors
	Parental expectations of diagnosis and treatment	Parents want evidence from "a test"
	Aid in explaining decision-making	Results reduce parental anxiety

For peer review only - http://bmjopen.bmj.com/site/about/guidelines.xhtml

	Parental anxiety may influence RDT usage	When don't parents trust a clinician's judgement
	RDTs role to decrease parental anxiety	Parents do not care what test is used
	Address parents' "fever-phobia"	
	RDT as reassurance	
	RDT use decision: a 'middle ground'	
	Decisions based on collaboration	
5. Determinants of Current POCT Usage	Parental expectations of diagnosis and treatment	Clinical decisions not influenced by fear of litigation
	Parental anxiety may influence RDT usage	Clinical decisions influenced by fear of litigation
	Decisions based on collaboration	Clinical decisions not influenced by parental expectations
	RDT use decision: a 'middle ground'	Clinical decisions influenced by parental expectations
	Colleague's Influence	Want evidence from "a test"
	Junior doctors Use More RDTs	Parental Concerns
	Decrease in frequency with seniority (doctors)	Young age of patient
	Tools for less experienced (doctors)	Unclear infectious foci
	Increase in frequency with autonomy (nurse)	
6. Characteristics of Future POCTs		
6a. Performance properties	Need to know reliability and specificity	More information from one POCT
	Importance of PPV and NPV	Results should make a difference in clinical course/management
	Need for test to change management	Should match gold-standard of lab-tests
	Need for decreasing ambiguity	Provides additional/different information from lab tests
6b. Practical use properties	Easy to act on binary results	Easy to interpret results
		Positive or negative results
		Numeric thresholds are ambiguous
		Need for analysers
		Minimal amounts of sample needed
		Painless/non-invasive for child
		Not time consuming to obtain
		Easier to get than lab-tests

For peer review only

Supplement 4: Example of summary matrix for the emergent theme "Trust in clinical acumen"

Participant	Initial coding	Sub-theme	Quote
ED_C2N	Assessment based decision	Patient management is based more on clinical findings than on diagnostic tests	To be honest with you, ED we really aren't going to get any diagnostic tests back. If the child is unwell, the child is going to be admitted. I might send some baseline inflammatory markers, so full blood count, CRP, Basic electrolytes, obviously blood cultures if their child is very febrile or if I'm concerned that they are shocked, urine sample. But beyond that, most of my management is going to be clinical grounds rather than treating the test result.
ED_N3N	Assessment based decision	Patient management is based more on clinical findings than on diagnostic tests	Even if the CRP was 5, that wouldn't mean you wouldn't then go on and do a septic screen. So it very much depends on the baby and how he looks and how he changes because...they change very very quickly.
ED_C1L	Assessment-based decision, RDT not in isolation	Diagnostic tests are just a complement of clinical assessment, they should not be used in isolation	So just the importance of making sure that it's still all about clinical assessment, and test are just an additional thing to that, that are really valuable and helpful.
ED_C1L	clinical acumen valued	Diagnostic tests are just a complement of clinical assessment, they should not be used in isolation	And inflammatory markers are a guide, but you know, we can get caught out where inflammatory markers can be low, and the child can still be very unwell. So it depends on the whole clinical acumen thing.
ED_C2L	Clinical acumen valued	?	I think you kind of go off clinically on what the case looks like.
ED_J2L	Clinical acumen valued	Patient management is based more on clinical findings than on diagnostic tests	...I'd prefer to be clinically led cuz if I was saying there was a number, CRP or the white count, which I may worry about sepsis. Then it could be argued that shouldn't you be worried even if those numbers are normal given that we know that the sensitive is particularly early in serious infection.
ED_N3N	Clinical acumen valued	Patient management is based more on clinical findings than on diagnostic tests	To obviously get the cultures and some things like that, and make sure we start them on sensitive to the particular infection they had. But, it would be a clinical determination on how unwell the child was and whether or not we were worried about them. It might put us a little direction on what the focus of the infection.
ED_J1N	Importance of examination and presentation	Patient management is based more on clinical findings than on diagnostic tests	I suppose there is always what you are seeing...you shouldn't be treating the numbers...you should be treating the patient so there is always a discrepancy between what you are seeing in your patient and your results. [J1N]
ED_C1L	Importance of examination and presentation	Patient management is based more on clinical findings than on diagnostic tests	Yeah so, first of all, it would be on the history and examination and what he looks like in front of me.
ED_N1L	Importance of examination and presentation	Sicker children can be identified clinically	And, it's usually, you're more aware of those children at triage, because usually the sicker children are having very abnormal obs. You can their respiratory efforts are a lot more increased. So you can usually differentiate between those children, roughly know that child...you know...this baby may need watching for 24 hours in our CDU, or it may need admission to the ward. You can usually differentiate between those two.
ED_J1N	Importance of examination and presentation	Patient management is based more on clinical findings than on diagnostic tests	Then I'd concentrate on my history and examination more than any other tests at that point.

For peer review only

ED_J1N	Importance of examination and presentation	Patient management is based more on clinical findings than on diagnostic tests	I think I always base the majority of my differential diagnosis on the history and examination really in a child like that.
ED_J1N	Importance of examination and presentation	Patient management is based more on clinical findings than on diagnostic tests	like I said, I think basically a lot of what I do is based on history and examination...the essential skills that doctors need to know. I trust my history and examination quite a bit.
ED_C1L	RDT not used in Isolation	Diagnostic tests are just a complement of clinical assessment, they should not be used in isolation	So I think clinical picture is still the most valuable tool and we have to make sure that people are trained in recognition of the unwell child, and use tests to augment them.
ED_C2L	RDT not used in Isolation	Diagnostic tests are just a complement of clinical assessment, they should not be used in isolation	people try to see CRP so they would use it as a kind of tracking rather than a stand alone. So, to be honest, I've not seen that many people treating anybody based on the CRP without some kind of clinical reasoning for doing so. If the patient looks unwell, you'd treat them.
ED_J1N	RDT not used in Isolation	Diagnostic tests are just a complement of clinical assessment, they should not be used in isolation	But I think if we do bloods, it is always together with history and examination just to aid your decision making. I don't think it makes your decision.
ED_C1N	RDT not used in Isolation	Diagnostic tests are just a complement of clinical assessment, they should not be used in isolation	Umm...yeah, I think that they can't be taken in isolation and I think that as long as they are used along with clinical judgement and after their assessment of the patient, taking into account all of the other factors such as their observations, their examination, the history that you've got, the parental concern, I think they are all very useful to make a clinical decision.
ED_C1N	RDT not used in Isolation	Diagnostic tests are just a complement of clinical assessment, they should not be used in isolation	Just the basic clinical skills that doctors...I think POCTs supplement what we do as doctors rather than replace it. I don't think they can be used in isolation.
ED_C1L	RDTs not used in Isolation	Diagnostic tests are just a complement of clinical assessment, they should not be used in isolation	I think that they can't be taken in isolation. I think that as long as they are used along with clinical judgement. And after their assessment of the patient. And taking into account all of the other factors such as their observations, their examination, the history that you've got, the parental concern. I think they are all very useful to make a clinical decision.
ED_C1L	RDTs not used in Isolation	Diagnostic tests are an alternative or complement of clinical assessment	So I think, you know, that's great that they are easy to do and that we get results fairly quickly but to think of the use of them in the same context where we happen to actually bleed a child...so we can use our clinical acumen as well.
ED_N2N	RDTs not used in Isolation	Diagnostic tests are just a complement of clinical assessment, they should not be used in isolation	A clinical reason to use it or if you are concerned about the patient and how they look. Then, just do the tests that are necessary so that you know you've made the right decision.
ED_C1N	RDTs not used in Isolation	Diagnostic tests are just a complement of clinical assessment, they should not be used in isolation	I don't think disruptive. Umm...as long as it's taken into context and that it's not taken in isolation.
ED_C1L	Tests and Observations (paired) as clinical tools	Clinical observation is used as a diagnostic tool	But we use that a lot. Any child with a fever without a very good focus. And even sometimes if they've got a respiratory tract focus, you might still want to make sure that we are...and if its clear. Often it's good to wait for them to wait for them to wee anyway. If there's a story of...umm....reduced fluid intake.

For peer review only

ED_C1L	Tests and Observations (paired) as clinical tools	Clinical observation is used as a diagnostic tool	what the point of doing the test is, explain what the results are, and say it's kind of here or there. And then, if the...again, if you are clinically worried, you would keep them for observation but maybe not treat.
ED_C1L	Tests and Observations (paired) as clinical tools	Clinical observation is used as a diagnostic tool	I can't remember exactly but maybe the CRP was 20-30. And then something else. But they were persistently tachycardic, but the urine was clear. And we do a chest x-ray and that was clear. And there was no meningitis or anything like that. And, the child didn't look septic or unwell. Just tachycardic. And in the end, we just observed them without treating them, and the tachycardic settled and then they went home. So, sometimes with pediatrics particularly, think, you just need a bit more time and such things.
ED_C1L	Tests and Observations (paired) as clinical tools	Clinical observation is used as a diagnostic tool	Regardless of how obvious the actual diagnosis is. So I think to help augment it, that would be really valuable though cuz it would help you maybe filter out some of those ambic [ambulatory] kids that you would admit but could actually could go home. You know, but then, actually, period observation is a good active treatment I think in pediatrics. I
ED_C2L	Tests and Observations (paired) as clinical tools	Clinical observation is used as a diagnostic tool	RDTs get people moving through quicker. Yeah. But I think in peds, we accept that our flow is often a bit disruptive. Cuz in pediatrics especially, there is quite a big role for a period of observation especially in febrile children.
ED_J1L	Tests and Observations (paired) as clinical tools	Clinical assessment and observation are more valuable than CRP	So I think that if I'm at the point where I want more information and I want to know what the CRP, I automatically want to observe them for a few hours, so it doesn't actually make that much of a difference to my working practice because I put less emphasis on the test than I do on the observations.
ED_J1N	Tests and Observations (paired) as clinical tools	Clinical observation is used as a diagnostic tool	So the child is usually just waiting with the parents. Sometimes it's useful because we can just observe them and see how they go for their fever or if we are waiting for anything else which is the urine sample or a fluid challenge. So sometimes, it doesn't affect the length of stay in the hospital cuz we were observing them anyway.
ED_C1N	Tests and Observations (paired) as clinical tools	Diagnostic tests are an alternative or complement of clinical assessment	So I think having useful tests and then documentation of how they have presented to you is essential. And whether that's the POCTs or observations and clinically document them in the notes the findings and what you've discussed.
ED_C2L	Trust in clinical judgement	Patient management is based more on clinical findings than on diagnostic tests	if they didn't, it would go more on how well they looked clinically.
ED_C1N	Trust in clinical judgement	Patient management is based more on clinical findings than on diagnostic tests	Where I can, I like to manage on my clinical judgement in looking at the child and my observational skills.
ED_C2L	Trust in clinical judgement	Patient management is based more on clinical findings than on diagnostic tests	But again, I don't find CRP that useful in that way, cuz I found that most people would go clinically on how the patient is like.
ED_J1N	Trust in clinical judgement	Patient management is based more on clinical findings than on diagnostic tests	I think you should base it on your clinical skills rather than investigating.

For peer review only

ED_C1N	Trust in clinical judgement	Patient management is based more on clinical findings than on diagnostic tests	I think it's good to use the test, but I think it doesn't replace clinical judgement and thorough clinical assessment.
ED_C1N	Trust in clinical judgement	Patient management is based more on clinical findings than on diagnostic tests	I think RDTs if you're concerned as a clinician, if even a test negative you should act on that concern.
ED_C1L	Trust in clinical judgement, RDT not used in isolation	Diagnostic tests are just a complement of clinical assessment, they should not be used in isolation	I guess, I try to base my differential diagnosis on clinical...on history and examination first. And if it's really clear, the POCTs augment what your management plan is going to do anyway. Cuz you're just going to treat based on that anyway.
ED_C1L	Trust in clinical judgement, RDT not used in isolation	Patient management is based more on clinical findings than on diagnostic tests	I guess anyway you go with your clinical picture,
CON1N	Clinical examination/impression still more trusted	Patient management is based more on clinical findings than on diagnostic tests	I think if we came back to that one, even if the CRP was low on POCT, I think I would still act because I know that, I know all the time that a lab CRP can be done and they can be low and we still get signs of an infection
SHO1L	Clinical examination/impression still more trusted	Clinical assessment and observation are more valuable than a POCT	And actually, I think the feeling is that having two hours with the patient is often more valuable than the result that you get back because you just get the time to see things for a bit longer, and just assess that baby or that child a few more times. […] I don't want to be able to see a child and send them home in 10 minutes, because I'll probably miss something. I'd much prefer if I saw a child and saw them for two hours, and pop my head in a few times and then have some blood results back that reassures me to let the child go home.
REG2N	Clinical examination/impression still more trusted	Clinical assessment and observation are more valuable than a POCT	I don't think so. Ummm, I think probably because on what I've touched on before. So I think the febrile children that you see fall into a few categories. So I think you get the children who are febrile and there is, you find a focus to their infection and based on that it's, it's normally, you're normally able to say if it's going to be more viral and kind of self-limiting, or this is more likely to be bacterial and you'd give the appropriate antibiotics depending on where the infection was. Then you get children who you can't find a focus but they look well. And if you've got a child who exams well with normal obs, then I would tend to do a period of observations, or if they look completely well, home with clear safety net advice for the parents to bring back if they became more unwell. Or I think you get the children who look sick and look septic, and in those children, you are going to treat with antibiotics. I was thinking, I couldn't think of like specific groups of children where a POCT would like really help you, help me decide on whether to give antibiotics or not.

COREQ (COnsolidated criteria for REporting Qualitative research) Checklist

A checklist of items that should be included in reports of qualitative research. You must report the page number in your manuscript where you consider each of the items listed in this checklist. If you have not included this information, either revise your manuscript accordingly before submitting or note N/A.

Topic	Item No.	Guide Questions/Description	Reported on Page No.
**Domain 1: Research team and reflexivity**			
Personal characteristics			
Interviewer/facilitator	1	Which author/s conducted the interview or focus group?	
Credentials	2	What were the researcher's credentials? E.g. PhD, MD	
Occupation	3	What was their occupation at the time of the study?	
Gender	4	Was the researcher male or female?	
Experience and training	5	What experience or training did the researcher have?	
Relationship with participants			
Relationship established	6	Was a relationship established prior to study commencement?	
Participant knowledge of the interviewer	7	What did the participants know about the researcher? e.g. personal goals, reasons for doing the research	
Interviewer characteristics	8	What characteristics were reported about the inter viewer/facilitator? e.g. Bias, assumptions, reasons and interests in the research topic	
**Domain 2: Study design**			
Theoretical framework			
Methodological orientation and Theory	9	What methodological orientation was stated to underpin the study? e.g. grounded theory, discourse analysis, ethnography, phenomenology, content analysis	
Participant selection			
Sampling	10	How were participants selected? e.g. purposive, convenience, consecutive, snowball	
Method of approach	11	How were participants approached? e.g. face-to-face, telephone, mail, email	
Sample size	12	How many participants were in the study?	
Non-participation	13	How many people refused to participate or dropped out? Reasons?	
Setting			
Setting of data collection	14	Where was the data collected? e.g. home, clinic, workplace	
Presence of non-participants	15	Was anyone else present besides the participants and researchers?	
Description of sample	16	What are the important characteristics of the sample? e.g. demographic data, date	
Data collection			
Interview guide	17	Were questions, prompts, guides provided by the authors? Was it pilot tested?	
Repeat interviews	18	Were repeat inter views carried out? If yes, how many?	
Audio/visual recording	19	Did the research use audio or visual recording to collect the data?	
Field notes	20	Were field notes made during and/or after the inter view or focus group?	
Duration	21	What was the duration of the inter views or focus group?	
Data saturation	22	Was data saturation discussed?	
Transcripts returned	23	Were transcripts returned to participants for comment and/or	

For peer review only

Topic	Item No.	Guide Questions/Description	Reported on Page No.
		correction?	
**Domain 3: analysis and findings**			
Data analysis			
Number of data coders	24	How many data coders coded the data?	
Description of the coding tree	25	Did authors provide a description of the coding tree?	
Derivation of themes	26	Were themes identified in advance or derived from the data?	
Software	27	What software, if applicable, was used to manage the data?	
Participant checking	28	Did participants provide feedback on the findings?	
Reporting			
Quotations presented	29	Were participant quotations presented to illustrate the themes/findings? Was each quotation identified? e.g. participant number	
Data and findings consistent	30	Was there consistency between the data presented and the findings?	
Clarity of major themes	31	Were major themes clearly presented in the findings?	
Clarity of minor themes	32	Is there a description of diverse cases or discussion of minor themes?	

Developed from: Tong A, Sainsbury P, Craig J. Consolidated criteria for reporting qualitative research (COREQ): a 32-item checklist for interviews and focus groups. *International Journal for Quality in Health Care*. 2007. Volume 19, Number 6: pp. 349 – 357

Once you have completed this checklist, please save a copy and upload it as part of your submission. DO NOT include this checklist as part of the main manuscript document. It must be uploaded as a separate file.

For peer review only

Supplement 6: Measures in place to ensure safe and efficient use of POCTs in the two hospitals

All point of care testing is overseen by a POCT committee that supervises point of care testing across the NHS trusts to which the two hospitals included in the study belong: Newcastle upon Tyne Hospitals NHS Foundation Trust with regards Great North Children Hospital in Newcastle, and Imperial College Healthcare NHS trust with regard St Mary's Hospital in London.

Within the two trusts, point of care testing is subject to strict governance and must be performed to the same quality standards as all testing undertaken within the United Kingdom Accreditation Service (UKAS) accredited laboratory departments, and to the same standards of the MHRA (Medicines and Healthcare products Regulatory Agency), the NHS Litigation Authority (NHSLA), the International Organisation for Standardisation (ISO), the Royal College of Pathologists (RCPath), and the Clinical Pathology Accreditation (CPA) standards.

Chaired by the POCT Manager, the POCT committee:

- Leads and coordinates POCT activities for the Trust in conjunction with the Trust's key objectives
- Maintains training records of all POCT operators throughout the Trust and coordinates staff education and facilitates training for all POCT devices. All POCT operators must be accredited by the POCT committee by undertaking POCT recognised training prior to patient testing. Competence in using POCT devices is achieved via Initial Training – this constitutes face-to-face training by either attending a timetabled training session or with a recognised 'Cascade Trainer'. Refresher Training is required every subsequent two years to maintain that competency
- Coordinates the provision of External Quality Assurance (EQA) schemes where possible, review EQA performance and offer advice on the improvement of poor performance
- Provides advice and help with documentation to Managers of areas on policies, operating procedures, training and competencies
- Undertakes Risk assessments for POCT devices where appropriate
- Leads and coordinates the validation of new POCT devices before implementation
- Provides evaluation of the performance of POCT equipment
- Coordinates audits and inspections to provide Trust assurance of quality patient care and value for money

For peer review only

Supplement 7: POCTs availability at the study hospitals

**Hospital**	**Test**	**Vendor**
St. Mary's Hospital, London	Blood gas analyser	GEM 4000
	Blood glucose/ketones	Freestyle Precision Pro
	Urinalysis	Siemens Multistix 8 SG
	CRP*	Alere Afinion AS100
Great North Children's Hospital, Newcastle	Urine dipstick	Combur7 test
	Urine ketones	Ketodiastix Ascensia
	Urine microscopy	
	Blood glucose	Accucheck inform II
	Blood gas analyser	GEM premier 5000
	Influenza & RSV	Cepheid GeneXpert

*CRP POCTs were not available at St. Mary's Hospital at the time of study, however they previously had been available as part of a pilot

For peer review only
